# Supplementary material for: Reasons for loss to follow-up (LTFU) of pulmonary TB (PTB) patients: A qualitative study among Saharia, a particularly vulnerable tribal group of Madhya Pradesh, India
Source: PLoS One. 2021 Dec 23;16(12):e0261152. doi: 10.1371/journal.pone.0261152 (PMC8699669; doi:10.1371/journal.pone.0261152)
Supplement: S1 Text — (DOC) [file pone.0261152.s001.doc]

**Fully confidential for research**

**“Identification of the factors leading to Loss to follow-up (LTFU) among saharia TB patients”**

**ICMR-National Institute for Research in Tribal Health, (ICMR) Jabalpur, (M.P).**

**LTFU-** **मरीज साक्षात्कार मार्गदर्शक**

**LTFU- Patient Interview guide**

**जिला………………………..** ……..**विकासखंड……………………………………….गांव……………………………………………….**

**District……………………………Block………………………………………… Village………………………………………**

1. **मरीज की जानकारी**
2. **Patient information:**
3. **नाम**  ……………………………………………….

Name: ……………………………………………….

1. **पिता/पति का नाम ………………………………………………………………………………..**

F/H Name: ……………………………………………….

1. उम्र ……………………………………………………………………………..

Age: ……………………………………………….

1. लिंग

Sex: पुरुष M…....1 महिला F………2 अन्य O……………3

1. घरेलु जानकारी

**Household Information:**

**(मुझे अपने परिवार के बारे में कुछ बताओ)**

**(Tell me something about your family)**

1. **आपके घर का मुखिया कौन है? उनका आपसे क्या रिश्ता है?**

Who is the head of your house? What is their relationship with you?

1. घर का प्रकार? (साक्षात्कारकर्ता स्वयं देख सकता है और भर सकता है)

House Type? **(Interviewer can see itself and fill it)**

1. आपके घर में ज्यादातर खाना पकाने का तरीका क्या है?

What is the mode of cooking in your house mostly?

1. आप घर में खाना कहाँ बनाते हैं?

Where do you make food at home?

1. घर में वेंटिलेशन (साक्षात्कारकर्ता स्वयं देख सकता है और भर सकता है)

Ventilation in the Household **(Interviewer can see itself and fill it)**

1. जीवन शैली के पात्र

**Life style characters**

1. **आपने कितनी पढ़ाई की है? आपकी शिक्षा क्या है?**

How much have you studied? What is your education?

1. क्या आप अभी कुछ काम करते हैं?

Do you do some work now?

1. पहले क्या काम करते थे?

What work did you do earlier?

1. एक महीने या उससे पहले कितनी कमाई होती है?

How much is earned in a month or earlier?

1. क्या आप अभी या पहले किसी भी तरह का नशा करते हैं?

Do you do any kind of addiction currently or earlier?

(व्यसन प्रकार- शराब, धूम्रपान और अन्य। कृपया विवरण में पूछें - प्रकार, आवृत्ति और कब से)

**(Addiction type- Alcohol, smoking and others. Please ask in details – type’s, frequency and since when).**

1. **नैदानिक ​​लक्षण (कृपया उपचार कार्ड देखें)**

**Clinical Characteristics (Please see treatment card)**

**Date of diagnosis: ………………………**

**Medicine starts date: ……………………………**

**RBS: ……………………………**

**HIV: ……………………………..**

**Height (CMS): …………………………..**

**Weight (Kg): ……………………………**

1. **टीबी का प्रकार?**

Type of TB?

ए. पल्मोनरी……………………………….1

1. Pulmonary………………..1

बी- अतिरिक्त पल्मोनरी ………2

1. Extra Pulmonary…………2
2. उपचार (कृपया उपचार कार्ड देखें)

**Treatments (Please see treatment card)**

**1- श्रेणी शुरू हुई ……………**

Category Started…………

1. दवा प्रतिरोधी स्थिति:

Drug resistant status:

ए. हाँ……….१ Yes………..1

बी. नहीं………2No………..2

1. प्रतिरोध के प्रकार

Types of Resistance

1. RR MDR………1
2. H Mono/Poly……..2
3. RR MDR + any FQ/SLID…3
4. XDR……….4
5. उपचार का चरण

Phase of Treatment

1. IP…………..1
2. CP………….2
3. दवा इतिहास

**Medication History**

1. **क्या आपने पहले टीबी की दवा ली है या आपको पहले भी यह बीमारी हो चुकी है? (यदि हाँ तो पूछें, कब)**

Have you taken TB medicine before or have you had this disease before? **(If yes ask, when).**

1. आपने इलाज कहां करवाया? (निजी या सरकारी)

Where did you get treatment? **(Private or Government).**

1. क्या आपने वह इलाज पूरा किया? (यदि नहीं, तो कारण पूछें)।

Did you complete that treatment? **(If no, ask reasons).**

1. आपको कैसे पता चला कि इलाज पूरा हो गया है?

How did you know the treatment was completed?

1. क्या आपके घर में या रिश्ते में किसी को पहले कभी यह बीमारी हुई है? (यदि हाँ, तो पूरी जानकारी पूछें)।

Has anyone in your house or in relationship, ever had this disease before? **(If yes, ask full detail).**

1. आपको क्या लगता है, यह बीमारी आपके घर कैसे पहुंची?

What do you think, how this disease reached your home?

1. वर्तमान में आपको और क्या कोई समस्या या बीमारी है?

What else do you have any other problems or illness currently?

कृपया मुझे अपने शुरुआती दिनों के बारे में विस्तार से बताएं?

**Please tell me about your early days in details?**

1. **शुरुआती दिनों में आपकी तबीयत कैसी थी?**

How was your health condition in early days?

1. टीबी के लक्षण कब आए?

When did the TB symptoms come?

1. जांच या पहचान कब और किसने की?

When and who did the investigated or identified?

1. आपको कब पता चला कि टीबी है?

When did you know that is TB?

1. क्या आपकी दवा शुरू हो रही है ?

Is your medicine being started?

1. दवा कब शुरू हुई थी?

When was the medicine started?

1. आपकी दवा कहाँ से और किसके द्वारा शुरू की गई है? (सरकारी या निजी)। (यदि निजी VIII पर जाएँ अन्यथा X पर जाएँ)

From where, and by whom, your medicine been started? **(Government or Private). (If private go to VIII otherwise go to X)**

1. आपने कितना खर्च किया? (यदि इसकी लागत है तो IX पर जाएं)

How much did you spend? **(If it costs then go to IX)**

1. जब सरकार में यह दवा मुफ्त में उपलब्ध है तो आप पैसे से दवा क्यों ले रहे हैं?

Why are you taking medicines with money, when this medicine available for free in the government?

1. कितने दिन दवा लेते हो?

How many days you take medicine?

1. आपका डॉट प्रदाता कौन है?

Who is your DOTs provider?

1. क्या डॉट्स प्रदाता आपके साथ अच्छा व्यवहार करता है और समय पर दवा उपलब्ध कराता है?

Is Dots provider treat you well and provide medicine on time?

1. क्या आप नियमित रूप से दवा ले रहे हैं? (यदि नहीं, तो तर्क अनुभाग पर जाएँ)

Are you taking medicine regularly? **(If No, go to reason section)**

1. फॉलो-अप खोने के कारण

Reasons for Lost to follow-up

1. कार्य संबंधी कारण:

Work related reasons:

1. क्या आपने इलाज शुरू करने से मना कर दिया है या छोड़ दिया है? (यदि हाँ, तो कारण पूछें)।

Are you quit or refused to start treatment? (If yes, ask Reason’s).

1. आपने कब तक इलाज शुरू किया या दवा लेना छोड़ दिया?

How long you left the treatments start or taking medicine?

1. आपको क्या लगता है; आपको टीबी नहीं है और बीमारी के कोई लक्षण नहीं हैं। क्या ये है दवा छोड़ने की वजह?

What do you think; you do not have TB and have no symptoms of the disease. Is this the reason for quit medicines? (कारण हो सकते हैं- काम का दबाव, समय की कमी, प्रवासन, कोई घरेलू कारण और अन्य कारण हो सकते हैं कृपया निर्दिष्ट करें)।

**(Reasons may be- Work pressure, Shortage of time, Migration, any domestic reasons and may be other reason please specify).**

1. **ड्रग एब्यूज**

**Drug abuse reason:**

1. **क्या दवा लेते समय आपको किसी तरह का कोई साइड इफेक्ट हुआ है? (यदि हाँ, तो इसके दुष्परिणामों का पता लगाएँ)।**

Have you had any kind of side effect, while taking medicine? (if Yes, find out side effects).

1. आपको क्या लगता है, इस बीमारी का लंबा कोर्स है, इसलिए आपने दवा छोड़ दी? (विवरण में पूछें)।

What do you think, is the long course of this disease, so you quit medicines? (Ask in details).

1. आपको क्या लगता है; आपको और दवा खानी है, तो आपने दवाई छोड़ दी?

What do you think; you have to eat more medicine, so you quit medicines?

1. व्यक्तिगत कारण

**Personal reasons**

1. **आपका टीबी का इलाज चल रहा है तो आप किसी को बताने में झिझकते हैं? (यदि हां, तो कारण पूछें)**

You are undergoing TB treatment then, you hesitate to tell anyone? **(If yes, Ask reason’s)**

1. क्या गांव वाले आपको शादी के किसी समारोह में या किसी सामुदायिक सभा में आमंत्रित करते हैं? (यदि नहीं तो कारण पूछें)

Do villagers invite you to any function of marriage or in any community gathering? **(if not, ask reason’s)**

1. क्या आपने इन दोनों वजहों से दवा छोड़ दी थी? (प्रश्न-1-2)

Did you quit medicine for both of these reasons? **(Que-1-2)**

1. क्या कोई आकर पूछता है कि आपने दवा क्यों छोड़ दी या दवा शुरू करने से मना कर दिया?

Is anyone came and ask, why you are quit medicine or refused to start medicine?

1. यदि हां, तो कितनी बार आए हैं ? और उन्होंने क्या सुझाव दिया?

If yes, how many times they are came? And what they suggested?

1. क्या वे लोग आपके साथ अच्छा व्यवहार कर रहे थे? (यदि नहीं, तो पूछें कि वे कैसा व्यवहार कर रहे थे)।

Were those people, treating you well? **(If not, ask how they were behaving).**

1. तुमने उनकी बात क्यों नहीं मानी और दवा छोड़ दी?

Why didn’t you listen to them and quit medicine?

1. क्या आप अभी कहीं और से दवा ले रहे हैं? (यदि हाँ, तो पूछें कहाँ से और क्यों?)

Are you taking medicine from elsewhere currently? **(If yes, ask from where and why?).**

1. क्या वह, दवा से ज्यादा फायदा हो रहा है? (यदि हाँ, तो पूछें कैसे?)

Is that, medicine benefiting more? **(If Yes, Ask how?).**

1. **क्या आप फिर से दवा शुरू करना चाहेंगे, या नियमित दवा लेना चाहेंगे, जो पूरी तरह से मुफ्त है? (यदि नहीं, तो कारण पूछें) (यदि हां, तो 11 पर जाएं)**

Would you like to start the medicine again, or taking regular medicine, which is completely free? **(if No, ask reasons) (If yes, go to 11)**

1. आपके बलगम के नमूने का फिर से परीक्षण किया जाएगा और दवा शुरू की जाएगी। (पूछें सहमत हैं या नहीं) (यदि नहीं तो कारण पूछें)।

Your sputum sample will be tested again and medicine will be started**. (Ask agree or not) (If not ask reasons).**

1. अगर आप दवा के सेवन के बारे में अधिक कहना चाहते हैं या नहीं, तो आप कह सकते हैं।

If you want to say more about intake medicine or not, then you can say.

**साक्षात्कारकर्ता का ना हस्ताक्षर तिथि**

**Interviewer Name Signature Date**

**……………………… …………………….. ……........**
