# Supplementary material for: Reasons for loss to follow-up (LTFU) of pulmonary TB (PTB) patients: A qualitative study among Saharia, a particularly vulnerable tribal group of Madhya Pradesh, India
Source: PLoS One. 2021 Dec 23;16(12):e0261152. doi: 10.1371/journal.pone.0261152 (PMC8699669; doi:10.1371/journal.pone.0261152)
Supplement: S2 Text — (DOC) [file pone.0261152.s002.doc]

**Fully confidential for research**

**“Identification of the factors leading to Loss to follow-up (LTFU) among saharia TB patients”**

**ICMR-National Institute for Research in Tribal Health, (ICMR) Jabalpur, (M.P).**

**LTFU रोगी परिवार के सदस्य साक्षात्कार गाइड**

**LTFU patient family member interview guide**

**जिला ………………………….. ब्लॉक ………………………गांव……………………………………………………….**

**District……………………………….. Block………………………………………… Village………………………………………………**

1. **परिवार के सदस्य की जानकारी:**

**Family member information:**

1. **नाम**  ……………………………………………….

Name: ……………………………………………….

1. **पिता/पति का नाम ………………………………………………………………………………..**

F/H Name: ……………………………………………….

1. उम्र ……………………………………………………………………………..

Age: ……………………………………………….

1. लिंग

Sex: पुरुष M…....1 महिला F………2 अन्य O……………3

1. शिक्षा (स्कूली शिक्षा के वर्ष):………………………………………………

Education (years of schooling):………………………………………………

1. मरीजों की जानकारी:

(कृपया मुझे रोगियों के बारे में कुछ बताएं)

**Patient’s information:**

**(Please tell me something about patients)**

1. **क्या आपके घर में पिछले 2-3 महीनों में कोई टीबी का मरीज आया है?**

Have you had any TB patient in your house in last 2-3 months?

1. क्या उनका इलाज चल रहा है? (यदि हां, तो कृपया उन्हें बताएं कि, हमारे रिकॉर्ड के अनुसार उनका इलाज नहीं किया जा रहा है)

Are they undergoing treatment? **(If yes, Please tell them that, according to our records they are not being treated)**

1. क्या वे नियमित रूप से दवा ले रहे हैं? (यदि हां, तो कृपया उन्हें बताएं कि, हमारे रिकॉर्ड के अनुसार वे दवा नहीं ले रहे हैं)

Are they taking medicine regularly?**(If yes, Please tell them that, according to our records they are not taking medicine)**

1. **मरीज के साथ आपका क्या रिश्ता है?**

What is your relationship with the patient?

1. क्या आप जानते हैं कि उन्हें कौन सी बीमारी है और वह कितनी खतरनाक है?

Do you know what disease they have and how dangerous is that?

1. क्या आपके घर में या रिश्ते में किसी को पहले कभी यह बीमारी हुई है? (यदि हाँ, तो पूरी जानकारी पूछें)

Has anyone in your house or in relationship, ever had this disease before? **(If yes, ask full detail).**

1. आपको क्या लगता है, यह बीमारी आपके घर कैसे पहुंची?

What do you think, how this disease reached your home?

**कृपया उन्हें उनके शुरुआती दिनों के बारे में विस्तार से बताएं?**

**Please tell them about their early days in details?**

1. **शुरुआती दिनों में उनकी तबीयत कैसी थी?**

How was their health condition in early days?

1. शुरुआती दिनों में उनकी तबीयत कैसी थी?

When did the TB symptoms come?

1. कब और किसने जांच या पहचान की?

When and who did the investigated or identified?

1. आपको कब पता चला कि टीबी है?

When did you know that is TB?

1. दवा कब शुरू हुई थी?

When was the medicine started?

1. उसने कितने दिन दवाई ली?

How many days did he take medicine?

1. क्या आपके मरीज नियमित रूप से दवा ले रहे हैं? या उन्होंने दवा शुरू की? (यदि नहीं, तो पूरी जानकारी पूछें)।

Are your patient, taking medicine regularly? Or did they start medicine? (**If no, ask full details).**

1. **वे नियमित रूप से दवा क्यों नहीं ले रहे हैं और उन्हें क्या परेशानी है? (कृपया मुझे विस्तार से बताएं)**

Why they are not taking medicine regularly and what trouble do they have? **(Please tell me in detail)**

1. **जब मरीज ने दवा लेने या लेने से मना कर दिया तो आपने क्या कार्रवाई की?**

What action did you take, when the patient refused to take or intake medicine?

1. **क्या आपने उन्हें उचित इलाज और दवा लेने के लिए प्रोत्साहित किया?**

Did you, encourage them to take proper treatment and medicine?

1. आप कितनी बार प्रोत्साहित करते हैं?

How many times you encourage?

1. आपको क्या लगता है, उसने दवा छोड़ दी?

What do you think, he left the medicine?

1. क्या कोई स्वास्थ्यकर्मी या कोई अन्य लोग आकर मरीज की काउंसलिंग कर रहे हैं?

Are any health worker or any other people came and counseling the patient?

1. **क्या रोगी कहीं और दवा ले रहा है? (यदि हाँ, कहाँ से? और क्यों?)**

Is patient, taking medicine elsewhere? **(If Yes, from where? And why?)**

1. आप एक मरीज के साथ कैसा व्यवहार करते हैं?

How do you treat a patient?

1. क्या आप रोगी के साथ रहते हैं, या रोगी को अलग रखते हैं?

Do you live with the patient, or Keep the patient separate?

1. क्या कारण हो सकता है, आपका रोगी किसी की नहीं सुनता?

What could be the reason, your patient not listening to anyone?

1. क्या आप मुझे कुछ और बताना चाहेंगे, रोगी ने दवा क्यों छोड़ी?

Would you like to tell me more about, something else, why the patient left the medicine?

**साक्षात्कारकर्ता का ना हस्ताक्षर तिथि**

**Interviewer Name Signature Date**

**……………………… …………………….. ……........**
