# Supplementary material for: Reasons for loss to follow-up (LTFU) of pulmonary TB (PTB) patients: A qualitative study among Saharia, a particularly vulnerable tribal group of Madhya Pradesh, India
Source: PLoS One. 2021 Dec 23;16(12):e0261152. doi: 10.1371/journal.pone.0261152 (PMC8699669; doi:10.1371/journal.pone.0261152)
Supplement: S3 Text — (DOC) [file pone.0261152.s003.doc]

**Fully confidential for research**

**“Identification of the factors leading to Loss to follow-up (LTFU) among saharia TB patients”**

**ICMR-National Institute for Research in Tribal Health, (ICMR) Jabalpur, (M.P).**

**उपचार समर्थक (डॉट्स प्रदाता) साक्षात्कार गाइड**

**Treatment supporter (DOTs provider) Interview guide**

**जिला ………………………….. ब्लॉक ………………………गांव……………………………………………………….**

**District……………………………….. Block………………………………………… Village………………………………………………**

1. **डॉट्स प्रदाता जानकारी:**

**Dots provider information:**

1. **नाम**  ……………………………………………….

Name: ……………………………………………….

1. **पिता/पति का नाम ………………………………………………………………………………..**

F/H Name: ……………………………………………….

1. उम्र ……………………………………………………………………………..

Age: ……………………………………………….

1. लिंग

Sex: पुरुष M…....1 महिला F………2 अन्य O……………3

1. शिक्षा (स्कूली शिक्षा के वर्ष):………………………………………………

Education (years of schooling):………………………………………………

I. मरीजों की जानकारी:

(कृपया मुझे रोगियों के बारे में कुछ बताएं)

**Patient’s information:**

**(Please tell me something about patients)**

1. **आप कैसे हैं? क्या हो रहा है?**

How are you? What is going on?

1. आप क्या काम करते हैं?

What work do you do?

1. आप कितने समय से डॉट्स प्रदाता के रूप में काम कर रहे हैं?

How long have you been working as DOTs Provider?

1. मुझे अपने काम (डॉट्स प्रदाता) के बारे में कुछ बताएं और आप एक मरीज के साथ कैसा व्यवहार करते हैं?

Tell me something about your work (DOTs provider) and how do you treat a patient?

1. कितने लोगों को, आपने आज तक दवाई दी है?

How many people, have you given medicine till date?

1. उनमें से कितने ठीक हुए और कितने लोगों ने दवा छोड़ दी?

How many of them got cured and how many people had given up the medicine?

1. अभी यहां कितने मरीज हैं?

How many patients are here right now?

1. क्या आप सभी मरीजों को दवा खिलाते हैं?

Do you feed medicine to all patients?

1. क्या सभी मरीज नियमित रूप से दवा ले रहे हैं?

Are all patients taking medicine regularly?

1. वर्तमान में कितने लोग, जो नियमित रूप से दवा नहीं ले रहे हैं?

How many people at present, who are not taking medicine regularly?

1. क्या कोई मरीज है, जिसने इलाज शुरू करने से मना कर दिया हो या लंबे समय से दवा नहीं मिल रही हो?

Is there any patient, who has refused to start the treatment or is not getting the medicine for a long time?

1. आप इन रोगियों से कहाँ मिले, जो दवाएँ नहीं ले रहे हैं?

Where did you meet these patients, who are not taking medicines?

कृपया उन्हें उनके शुरुआती दिनों के बारे में विस्तार से बताएं?

**Please tell them about their early days in details?**

1. **शुरुआती दिनों में उनकी तबीयत कैसी थी?**

How was their health condition in early days?

1. टीबी के लक्षण कब आए?

When did the TB symptoms come?

1. कब और किसने जांच या पहचान की?

When and who did the investigated or identified?

1. आपको कब पता चला कि टीबी है?

When did you know that is TB?

1. दवा कब शुरू हुई थी?

When was the medicine started?

1. उसने कितने दिन दवाई ली?

How many days did he take medicine?

1. मरीजों ने दवा क्यों छोड़ दी या दवा खाने से मना कर दिया? (विस्तार से बताएं)

Why patients are quit medicine or refused to eat medicine? **(Tell me in detail)**

1. जब मरीज ने दवा लेने या लेने से मना कर दिया तो आपने क्या कार्रवाई की?

What action did you take, when the patient refused to take or intake medicine?

1. आपके अलावा किसी और ने मरीज को दवा लेने के लिए राजी किया? (यदि हाँ, तो कौन? और वह कहाँ से आया है)।

Someone other than, you persuaded the patient to take medicine? **(If yes, who? And from where he came).**

1. क्या कोई अस्पताल या किसी अन्य स्वास्थ्यकर्मी से मरीजों की काउंसलिंग करने आया था?

Did anyone come from hospital or any other health worker to counsel the patients?

1. वह कितनी बार आए और उन्होंने रोगी को क्या सुझाव दिया?

How many times did he came and what they suggested to patient?

1. क्या मरीज, अभी कहीं और दवा ले रहा है? (यदि हाँ तो कहाँ से ?)

Is patient, currently taking medicine elsewhere? **(If Yes, from where?)**

1. आपको क्या लगता है, मरीज ने दवा छोड़ दी और दवा शुरू क्यों नहीं की?

What do you think, the patient left the medicine and why not started the medicine?

1. क्या आप इस बारे में और जोड़ना चाहेंगे कि मरीज ने दवा क्यों छोड़ी?

Would you like to add more about, why the patient left the medicine?

**साक्षात्कारकर्ता का ना हस्ताक्षर तिथि**

**Interviewer Name Signature Date**

**……………………… …………………….. ……........**
