# Supplementary material for: Reasons for loss to follow-up (LTFU) of pulmonary TB (PTB) patients: A qualitative study among Saharia, a particularly vulnerable tribal group of Madhya Pradesh, India
Source: PLoS One. 2021 Dec 23;16(12):e0261152. doi: 10.1371/journal.pone.0261152 (PMC8699669; doi:10.1371/journal.pone.0261152)

PLOS ONE

Factors leading to loss to follow-up (LTFU) of pulmonary TB (PTB) patients: A Study among Saharia a particularly vulnerable tribal group of Madhya Pradesh, India

--Manuscript Draft--

**Manuscript Number:**	PONE-D-20-21794
**Article Type:**	Research Article
**Full Title:**	Factors leading to loss to follow-up (LTFU) of pulmonary TB (PTB) patients: A Study among Saharia a particularly vulnerable tribal group of Madhya Pradesh, India
**Short Title:**	Factors leading to Loss to follow up cases of Tuberculosis
**Corresponding Author:**	Jyothi Bhat<br>National Institute for Research in Tribal Health<br>Jabalpur, Madhya Pradesh INDIA
**Keywords:**	Lost to follow up, adherence, treatment, Tuberculosis, Saharia
**Abstract:**	<p><div class="abstract"> <div><h1 class="heading">Background</h1> <p>Loss to follow up (LTFU) of pulmonary tuberculosis (PTB) patients is a major challenge for TB control. However, there is dearth of information about the factors leading to LTFU among marginalized communities. This study highlights the factors associated with LTFU in Saharia, a tribe of Madhya Pradesh having high tuberculosis (TB) prevalence.</p> <h1 class="heading">Methods</h1> <p>A qualitative study was carried out during January-April 2020 among twenty-two pulmonary TB patients, recorded as LTFU in NIKSHAY, along with 10 treatment supporters and 10 patient’s family members. Semi structured personal interview tools were used to collect the information and interviews were transcribed and inductive content analysis was conducted to originate promising themes.</p> <h1 class="heading">Results</h1> <p>The study explored various social, behavioral factors leadings to loss to follow-up among <i>Saharia</i> PTB patients. Drug side effects, alcoholism, social stigma, lack of awareness to seriousness of disease and poor counseling are the main barricades to treatment adherence in this community.</p> <h1 class="heading">Conclusions</h1> <p>The study highlights the need of addressing the issues related to LTFU during the course of TB treatment. The enhanced efforts of treatment supporter, health staff and Family & community persons are required to motivate and support the patients.</p> </div> </div></p>
**Order of Authors:**	Prashant Mishra
	Ravendra K Sharma
	Rajiv Yadav
	VG Rao
	Samridhi Nigam
	Mercy Aparna Lingala
	Jyothi Bhat
**Additional Information:**	
**Question**	**Response**
**Financial Disclosure**<br><br>Enter a financial disclosure statement that describes the sources of funding for the work included in this submission. Review the submission guidelines for detailed requirements. View published research articles from *PLOS ONE* for specific	The study is financially supported by the Government of Madhya Pradesh (Budget 2210/2017-18/877 dated_27/01/18). However, the funding agency had no role in the study design, data collection, analyses, interpretation of results, reports & manuscript writing and submission to the journal.

Powered by Editorial Manager® and ProduXion Manager® from Aries Systems Corporation

examples.<br><br>This statement is required for submission and **will appear in the published article** if the submission is accepted. Please make sure it is accurate.<br><br>**Unfunded studies**<br>Enter: *The author(s) received no specific funding for this work.*<br><br>**Funded studies**<br>Enter a statement with the following details:<br>• Initials of the authors who received each award<br>• Grant numbers awarded to each author<br>• The full name of each funder<br>• URL of each funder website<br>• Did the sponsors or funders play any role in the study design, data collection and analysis, decision to publish, or preparation of the manuscript?<br>• **NO** - Include this sentence at the end of your statement: *The funders had no role in study design, data collection and analysis, decision to publish, or preparation of the manuscript.*<br>• **YES** - Specify the role(s) played.<br><br>* typeset	
**Competing Interests**<br><br>Use the instructions below to enter a competing interest statement for this submission. On behalf of all authors, disclose any competing interests that could be perceived to bias this work—acknowledging all financial support and any other relevant financial or non-financial competing interests.<br><br>This statement **will appear in the published article** if the submission is accepted. Please make sure it is accurate. View published research articles from *PLOS ONE* for specific examples.	The authors have declared that no competing interests exist

**NO authors have competing interests**<br><br>Enter: *The authors have declared that no competing interests exist.*<br><br>**Authors with competing interests**<br><br>Enter competing interest details beginning with this statement:<br><br>*I have read the journal's policy and the authors of this manuscript have the following competing interests: [insert competing interests here]*<br><br>* typeset	
**Ethics Statement**<br><br>Enter an ethics statement for this submission. This statement is required if the study involved:<br><br>• Human participants<br>• Human specimens or tissue<br>• Vertebrate animals or cephalopods<br>• Vertebrate embryos or tissues<br>• Field research<br><br>Write "N/A" if the submission does not require an ethics statement.<br><br>General guidance is provided below. Consult the submission guidelines for detailed instructions. **Make sure that all information entered here is included in the Methods section of the manuscript.**	This study was carried out as a part of the main ongoing study "Intensified Tuberculosis Control Project among Saharia of Madhya Pradesh". The ongoing TB control project is approved by Institutional Ethics Committee (IEC) with a reference no. NIRTH/IEC/2273/2016. Participants who were willing to participate and provided written informed consents were enrolled as study participants. The manuscript is also approved by institute publication committee (ICMR-NIRTH/PSC/46/2020).

Powered by Editorial Manager® and ProduXion Manager® from Aries Systems Corporation

**Format for specific study types**<br><br>**Human Subject Research (involving human participants and/or tissue)**<br>• Give the name of the institutional review board or ethics committee that approved the study<br>• Include the approval number and/or a statement indicating approval of this research<br>• Indicate the form of consent obtained (written/oral) or the reason that consent was not obtained (e.g. the data were analyzed anonymously)<br><br>**Animal Research (involving vertebrate animals, embryos or tissues)**<br>• Provide the name of the Institutional Animal Care and Use Committee (IACUC) or other relevant ethics board that reviewed the study protocol, and indicate whether they approved this research or granted a formal waiver of ethical approval<br>• Include an approval number if one was obtained<br>• If the study involved *non-human primates*, add *additional details* about animal welfare and steps taken to ameliorate suffering<br>• If anesthesia, euthanasia, or any kind of animal sacrifice is part of the study, include briefly which substances and/or methods were applied<br><br>**Field Research**<br><br>Include the following details if this study involves the collection of plant, animal, or other materials from a natural setting:<br>• Field permit number<br>• Name of the institution or relevant body that granted permission	
**Data Availability**<br><br>Authors are required to make all data underlying the findings described fully available, without restriction, and from the time of publication. PLOS allows rare exceptions to address legal and ethical concerns. See the PLOS Data Policy and FAQ for detailed information.	No - some restrictions will apply

Powered by Editorial Manager® and ProduXion Manager® from Aries Systems Corporation

A Data Availability Statement describing where the data can be found is required at submission. Your answers to this question constitute the Data Availability Statement and **will be published in the article**, if accepted.<br><br>**Important:** Stating 'data available on request from the author' is not sufficient. If your data are only available upon request, select 'No' for the first question and explain your exceptional situation in the text box.<br><br>Do the authors confirm that all data underlying the findings described in their manuscript are fully available without restriction?	
**Describe where the data may be found in full sentences. If you are copying our sample text, replace any instances of XXX with the appropriate details.**<br><br>• If the data are **held or will be held in a public repository**, include URLs, accession numbers or DOIs. If this information will only be available after acceptance, indicate this by ticking the box below. For example: *All XXX files are available from the XXX database (accession number(s) XXX, XXX.)*.<br>• If the data are all contained **within the manuscript and/or Supporting Information files**, enter the following: *All relevant data are within the manuscript and its Supporting Information files.*<br>• If neither of these applies but you are able to provide **details of access elsewhere**, with or without limitations, please do so. For example:<br><br>*Data cannot be shared publicly because of [XXX]. Data are available from the XXX Institutional Data Access / Ethics Committee (contact via XXX) for researchers who meet the criteria for access to confidential data.*<br><br>*The data underlying the results presented in the study are available from (include the name of the third party*	Data is available in the qualitative format and hence cannot be shared here. However it is available with PI of the project and will be available after considering confidentiality aspect.

Powered by Editorial Manager® and ProduXion Manager® from Aries Systems Corporation

and contact information or URL).<br>• This text is appropriate if the data are owned by a third party and authors do not have permission to share the data.<br><br>* typeset	
Additional data availability information:	

Manuscript

Click here to access/download;Manuscript;LTFU_Manuscript_230620v3.doc

Factors leading to loss to follow-up (LTFU) of pulmonary TB (PTB) patients: A Study among *Saharia* a particularly vulnerable tribal group of Madhya Pradesh, India

Prashant Mishra, Ravendra K Sharma, Rajiv Yadav, VG Rao, Samridhi Nigam, Mercy Aparna. Lingala and Jyothi Bhat*,

ICMR-National Institute of Research in Tribal Health, Jabalpur

Corresponding author:

Dr. Jyothi Bhat, M.D.
ICMR-National Institute of Research in Tribal Health
Nagpur Road
P.O. Garha
Jabalpur 482003
Phone: +91 761 2370800
Email: drjyothibhat@rediffmail.com

ABSTRACT

Background: Loss to follow up (LTFU) of pulmonary tuberculosis (PTB) patients is a major challenge for TB control. However, there is dearth of information about the factors leading to LTFU among marginalized communities. This study highlights the factors associated with LTFU in Saharia, a tribe of Madhya Pradesh having high tuberculosis (TB) prevalence.

Methods: A qualitative study was carried out during January-April 2020 among twenty-two pulmonary TB patients, recorded as LTFU in NIKSHAY, along with 10 treatment supporters and 10 patient's family members. Semi structured personal interview tools were used to collect the information and interviews were transcribed and inductive content analysis was conducted to originate promising themes.

Results: The study explored various social, behavioral factors leadings to loss to follow-up among *Saharia* PTB patients. Drug side effects, alcoholism, social stigma, lack of awareness to seriousness of disease and poor counseling are the main barricades to treatment adherence in this community.

Conclusions: The study highlights the need of addressing the issues related to LTFU during the course of TB treatment. The enhanced efforts of treatment supporter, health staff and Family & community persons are required to motivate and support the patients.

Funding: The study was carried out as a sub-study under the ongoing study on Intensified Tuberculosis Control Project among Saharia: A Particularly Vulnerable Tribal Group in Madhya Pradesh. The main study is funded by the Government of Madhya Pradesh.

INTRODUCTION

India is the highest TB burden country in the world accounting for about a quarter of world's TB cases.[1] Though the Revised National Tuberculosis Control Programme (RNTCP), now National Tuberculosis Elimination Programme (NTEP), has been successfully implemented in India for more than two decades, tuberculosis is still a major challenge in the country, especially in rural and remotes areas. DOTS is a back bone of India's TB elimination programme with treatment compliance as one of the major strategy.[2] Poor compliance to tuberculosis treatment is a major obstacle for TB elimination programme as it can increase the risk of drug resistance and prolong infectiousness and may result in unfavourable outcomes such as treatment failure, death and relapse, thus posing a public health threat.[3,4] This is particularly relevant for tribal population, an underprivileged group of the society residing usually in remote rural areas thus having a poor access to the health delivery system.

Loss to follow up (LTFU) has been defined by the World Health Organization (WHO) as "patient whose treatment was interrupted for two consecutive months or more"[5]. LTFU is a result of several factors such as access to health services, socioeconomic status, literacy level and beliefs and practices prevalent in the society.[6,7] Several other studies conducted earlier in different settings have also identified many factors responsible for LTFU such as illiteracy, poverty, long duration of treatment, large number of medicines, access to health services, work related issues, smoking & alcoholism, beliefs & practices etc. [3,8,9].However, limited studies on the factors responsible for LTFU among TB patients are available in India especially from resource-constrained settings including remote tribal areas.

Madhya Pradesh (MP) state in central India alone accounts for 14.7% of country's total tribal population[10] and tribes are consistently having poor health seeking behavior and access to the health services[11] Tuberculosis is a key public health problem among *Saharia* tribe - a Particularly Vulnerable Tribal Group (PVTG) of Madhya Pradesh. The studies carried out among this tribe reported very high prevalence of pulmonary tuberculosis (PTB), 1270 in 1991-92[12], 1518 in 2008 [13] and 3294 in 2015[14] per 100,000 in selected *Saharia* areas. There is no

information available on the factors associated with LTFU of TB patients amongst Saharia tribal population.

We therefore conducted a qualitative study to obtain better understanding of TB patients, family members and treatment provider related factors for LTFU among Saharia tribe of Madhya Pradesh, central India.

METHODS

Study setting

The study was carried out as sub-study of the ongoing integrated TB control project in *Saharia* population residing in seven districts of Gwalior and Chambal divisions of Madhya Pradesh state in central India. The districts are linked to drug resistant TB center (DRTBC) Gwalior, Madhya Pradesh. Drug sensitive (DS) TB patients are provided medicine through DOT providers and linked to the nearest TB DOT center, whereas, drug resistant (DR) TB patients are treated at district TB center (DTC) initially during pre-treatment evaluation period and later receive treatment from the nearest TB DOT center.

Study design and sampling

Thirty Eight PTB (DS and DR) patients (31 males and 7 females)were recorded as LTFU in NIKSHAY, from the list of TB patients registered for treatment under integrated TB control project during January-October 2019. All LTFU patients were visited by survey team during January-April 2020 and among those five were found dead, three migrated to other districts and eight LTFU patients were on re-treatment. Rest all twenty-two LTFU patients (19 males and 3 females) were successfully interviewed. Ten treatment supporters and ten patients' family members were also interviewed. Purposive sampling was used to ensure variety of patients' family members and treatment supporters and to capture the maximum variation in responses of treatment supporters and family members. All qualitative in-depth interviews were performed using semi-structured interview guide.

Data collection and analysis

In-depth interviews were conducted in local regional language (dialect of *Hindi*) by project scientists (PS) and district coordinators (DC's) of the project who were trained in TB diagnosis and treatment guidelines. The semi structured interview tools for patients, treatment supporters and patient's family members include their background information, information on TB patient's previous history of anti-tuberculosis treatment (ATT), adverse drug events (ADE), social cognitive, behaviors, myths and misbeliefs that determine adherence and factors associated with loss to follow-up. All study participants were interviewed in the isolation at a place convenient to them and their audio-recorded responses were transcribed in English language. The codes and themes were developed alongside with data collection. Some direct verbatim quotes that showed important responses/ factors also presented in the manuscript.

Ethical considerations

This study was carried out as a part of the main ongoing study "Intensified Tuberculosis Control Project among Saharia of Madhya Pradesh". The ongoing TB control project is approved by Institutional Ethics Committee (IEC) with a reference no. NIRTH/IEC/2273/2016. Participants who were willing to participate and provided written informed consents were enrolled as study participants. The manuscript is also approved by institute publication committee (**ICMR-NIRTH/PSC/46/2020**).

RESULTS

Socio-demographic profile and living conditions of participants

The study participants comprised 22 TB patients reported as LTFU patients in NIKSHAY, aged 25 to 70 years. This includes 19 male and 3 female patients, and all belonging to *Saharia* tribal community. Among the LTFU patients, thirteen patients were drug resistant - 2 H-Mono resistant, 9 Rifampicin resistant or RR-MDR and 2 were XDR. Majority (18/22) of the patients were underweight (BMI <18.5) and previously treated (19/22). Most of respondents are illiterate and work as manual labours at stone quarries, agriculture fields and road/house constructions or other development projects which is their main source of livelihood. They also migrate to nearby districts and states in search of work/jobs and remain outside of their villages for a substantial

~~period of the year (Table-I). Most of their houses are Kutcha houses (walls and/or roof made of mud/stone) or huts (thatched walls and/or roof) with single room and mostly without any ventilation. The same room is also used for cooking and main fuel used for cooking is wood or crop residuals producing lot of smoke which is one of the major risk factor of pulmonary tuberculosis (PTB)(Table-I).~~

~~The in-depth interviews of participants identified a number of factors leadings to LTFU in the *Saharia* community. Themes that are developed during analysis of in-depth interviews are summarized in Table-II and important verbatim are described below-~~

Adverse drug effects

Out of twenty-two patients, eighteen reported adverse drug effects as a significant barricade for treatment adherence. All patients, treatment providers and patient's family members reported drug's side effects such as vomiting, severe headache, vertigo, stomach ache, nausea and hemoptysis. ~~However, relatively patients reported more adverse drug effects as an important reason for discontinuing the treatment compared to treatment providers and family members.~~

A 30 years old male reported – *"When I used to take medicines, I used to get blood out of my mouth, I used to vomit and had trouble in breathing! So, I quit the medicine."* [Patient 1, Male, 30 years].

Another 45 years old male reported- *"Whenever I took medicine, there was a severe pain in the stomach, a burning sensation in the urine, the medicine used to heat a lot! So, I quit the medicine."* [Patient 3, Male, 45 years].

The burning sensation and diarrhea were also commonly reported side effects of drugs, A 34 years of male reported –

"When I used to take pills, I had severe pain in the stomach, vomiting and diarrhea, there was a burning sensation in the stomach, and also feelt dizziness and nervousness. So, I quit this medicine." [Patient 10, Male, 34 years].

A 42 years family member (wife) told - *"He was not taking medicines because he was unhappy with medicine, he had burning sensation in his stomach and nervousness"* [Family member 4, Female, 42 years].

A 25 years treatment supporter reported - *"Patients do not take medicine properly because medicine heats and vomiting and diarrhea occur, so they quit medicine after few days and approach local gunia (traditional healer) or private practioner)"* [Treatment supporter 6, Male, 25 years].

High Pill burden and drug quality

During interview sessions, we also found that in addition to adverse drug effects, high pill burden is also an issue for LTFU in this community. The daily regimen of 9-11 pills with injection made it difficult for some patients to complete treatment. A patient reported –

"Though I had to take 8-10 pills every day, I was taking these pills but I was also asked to take injections. As I was already weak, I used to get dizziness after injection and go into slumber" [Patient 8, Male, 45 years].

Another patient complained of the quality of the drugs and reported- *"The pills were not of good quality, I had to take 8 pills, it was troublesome to take these medicine regularly"* [Patient 10, Male, 34 years].

Work related and financial constraint

~~Work associated problems~~ are also identified as one of the reasons ~~for discontinuation~~ of the treatment. Fourteen patients reported that they had to quit their job ~~for starting~~ the treatment. Some patients mentioned difficulties in carrying out their works after taking medicines.

A 35 years old patient reported *"I used to go to stone quarry work. Since I got sick and I am unable to go for work. Earlier, I used to earn Rs. 50-100 daily, now that too is gone"* [Patients 17, Male, 35 years].

A 50 years old male mentioned, *"When I was taking medicine, I used to get dizziness and I was afraid that I wouldn't get my wages"* [Patient 16, Male, 50 years].

Some of them are working in stone quarries and take payments in advance, so they have to continue the work even when they are sick. A 42 years old laborer reported –

"As I have taken payment in advance *and working away from my home, I cannot go to my house, so I quit medicine"* [Patient 20, Male, 42 years].

Alcohol abuse and smoking

~~Habits like alcohol use and tobacco consumption is very common among *Saharia* community. During the interviews, many patients reported alcohol consumption and smoking as a factor for not adhering to treatment. These were also reported by treatment supporter (DOTS providers) and patient's family members. However, after getting disease, many of them also stopped smoking and alcohol consumption.~~

A 50 years old male reported *"Earlier, I used to drink liquor, but now I have not consumed liquor from last 2-3 years. Now, I smoke only 2-4 Beedi per day, I have been smoking since I was 10-12 years old."* [Patient 9, Male, 50 years].

Another patient mentioned - *"Earlier I used to smoke Ganja* (Marijuana*) and I smoked for 4-5 years, but now I have given up since last 3-4 months"* [Patient 10, Male, 34 years].

A 35 years old female (patient's wife) reported - "*My husband is not taking medicine because he drinks liquor. I have explained to him many times about bad effects of liquor, but he does not listen to me. He is still suffering from disease, he coughs all the day"* [Family member 2, Female, 35 years].

A treatment supporter said, "*Many patients drink liquor and smoke (Beedis) during the course of treatment, due to which the medicine cause more side effects (adhik garmi karti hai) and they*

give up the medicine instead of stopping drinking and smoking" [Treatment supporter 4, Male, 29 Year].

However, some of them also reported that they are using alcohol to suppress the symptoms of the TB disease, especially the cough.

A 45 years old male told - *"Yes, I drink liquor, I drink to suppress cough and I take approximately 200 ml at a time."* [Patient 3, Male, 45 years].

Stigma and family support

~~Social stigma, discouragement and poor family support are also reported as major causes and barriers for not completing the TB treatment. The fears of discrimination by their own family members directly affect the activities to promote adherence and TB treatment outcomes. During the interview sessions, few TB patients reported that they did not want health care workers to visit their home for counseling and even they did not want to visit their local or block and district level DOT center, due to fear of disclosure of their disease status.~~

A 38 years old female reported - *"When my TB treatment started, I was very ashamed to tell my neighbor and the villagers. I do not know why, but there was always a fear; I did not tell anyone my treatment was for what"* [Patient 2, Female, 38 years].

A family member (patient's wife) told - *"We live separately, though he is my husband. He lives in a hut outside the house, because it is a communicable disease"* [Family member 2, Female, 35 years].

A male patient also mentioned - "*When I got sick, no one from my family was willing to accompany me to the hospital. Only my wife was willing to come, but I did not take her to hospital because she had never visited a hospital, she does not know anything"* [Patient 1, Male, 30 years].

Myths and misbeliefs

Lack of awareness to the seriousness of ~~disease~~ and its treatment also influenced treatment adherence adversely. Many patients who did not complete their treatment had myths and misbeliefs associated with the TB disease and treatment. Some patients do not trust diagnosis and continue to believe that they do not have TB disease. So, they don't start treatment or stop taking medicine after few days of treatment initiation. A 45 years old patient reported –

"*I do not have any disease, so why should I take medicine."* [Patient 13, Male, 45 years].

However, some also believed that taking medicine or anti-TB treatment will further deteriorate their health.

A 50 years old male reported, *"I would have not been able to sit again if I had taken these pills and would have died. People in my family also used to get upset and used to say why I should take such pills"* [Patient 9, Male, 50 years].

Another patient revealed - "*My younger brother had the same disease (TB). He also took medicine; but the medicines he was taking were different. My medicine caused stomach ache and burning sensation in the urine. My medicine was not good, he got better medicines"* [Patient 6, Male, 45 Year].

Some patients also expressed peer pressure to stop medicines. A 60 years old male reported –
"I was taking medicine for a long time and when I started getting blood in the cough, my neighbors and villagers asked me to stop taking too much medicines. So, I quit medicine" [Patient 14, Male, 60 years].

During interviews, it was also observed that patients, care providers and family members also correlate misfortunes with TB disease or its treatment. A 38 years old female patient who lost her child during the treatment reported –

“*When I was taking medicines, my son died; I was very much disheartened and left the medicines. When my son is no more, then for whom should I take medicines?”* [Patient 2, Female, 38 years].

Challenges and barriers (Representative’s quotations)

Treatment supporters and patient’s family members were also interviewed to identify the challenges and barriers leading to LTFU. Some of them mentioned that long duration of treatment, alcoholism and smoking are important causes of LTFU. As reported by one treatment supporter –

“*As far as I have experienced, the medicines have adverse effects (garmi karti hai) and the duration of treatment is prolonged. So, patients take medicine for one or two months and then they quit medicine”* [Treatment supporter 1, Male, 20 years].

A 29 years old male reported -

“Many patients drink liquor daily and get drunk and do not take medicines regularly. Subsequently they stop medicine [Treatment supporter 4, Male, 29 years].

Some patients stop medicine after taking one-two months when symptoms get subsided, thinking that they are disease free and thus do not take full course of treatment. A treatment supporter reported –

“*When patients take proper medicine regularly, they feel better within a month, and they start thinking that they have recovered from the disease and quit medicines”* [Treatment supporter 2, Male, 20 years].

“After taking medicines for 1-2 months, patient’s health improves and he think that he is cured, and start avoiding medicines, and finally stop the medicine” [Treatment supporter 3, Male, 35 years].

DISCUSSION

This is the first reported qualitative study that provide insight on the causes of LTFU of TB patients among *Saharia*- a PVTG in Madhya Pradesh having very high TB burden. The study focused on in-depth analyses of narrative data from patients, treatment supporters and patient's family members. Our findings revealed that approaching the health facility for diagnosis and treatment initiation (step-III) and enrolment/follow-up (step-IV) are very crucial for treatment adherence among TB patients (Fig-I).

We found that patient's judgment of the severity of their fatigue; dizziness and vomiting and high pill burdens are also leadings to LTFU among *Saharia*. The adverse drug events (ADE) and high pill burden of TB regimen promote discontinuation of treatment or patients shift to private TB treatment. The poor awareness to drug's possible side effects, absence of counseling before initiation of the drugs contribute to the LTFU. Without proper counseling, patients drift out of the system and discontinue the treatment. Several other studies also reported similar findings among TB patients[3,15-19]. In the community setting, particularly in remote tribal areas, when ADE occurs, patients need advice and counseling but they hardly find any health staff nearby to support them. So, they are compelled to approach local practitioners and traditional healers who are easily available in the hamlet/locality. Our findings show that the patients were not fully aware of the possible side effects of drugs and so they were not aware how to cope with these adverse effects, particularly in the intensive phase of treatment. Other studies have also reported similar findings in different regions [16,17,20]. The possible reason for higher ADE among Saharia tribe could be high prevalence of undernutrition among them. The BMI rates are lower in this tribe and deprivation of adequate nutrition is likely to lead to severe reactions like vomiting and nausea, thus promoting discontinuation of TB treatment among patients. The pre-treatment counseling and active surveillance of patients' adverse reactions by the health staff are crucial to improve treatment compliance.

Our work shows that alcohol consumption during the course of treatment is a major barrier for treatment compliance in Saharia tribe. The findings are consistent with previous investigations. Alcoholism has been reported as a significant factor of patient non-compliance or adverse treatment outcomes among tuberculosis patients receiving DOTS treatment [19,21-24] In view of these findings, the history of alcoholism before starting treatment would be useful in identifying

potential defaulters. We also found smoking leading to treatment discontinuation in the present study. Similar findings were observed among TB patients receiving standard TB regimen in different settings[22,25].

~~The Saharia tribe is one the marginalised community of Madhya Pradesh and they depend on daily work for their livelihood.~~ Most of the LTFU patients are either primary earners or are in ~~economic~~ active ages, work primarily on the daily wages. ~~The financial constraints and personal reasons related to work, i.e. fear of losing job and migration within the country for job are also key factors responsible for loss to follow up and not completing the treatment.~~ Several other studies also reported work and financial constraint as the possible reasons for treatment discontinuation[16, 21,. 26-28].

~~Saharia tribe is a particularly vulnerable tribal group of Madhya Pradesh with extensive illiteracy, our study also showed that most of the study participants are illiterate.~~ Thus, incorrect knowledge or myths and misconceptions about TB disease and its medications and treatments are common among patients and family members. ~~The low literacy could also be the reason for their incorrect knowledge about the TB disease as has been reported in several studies~~[19,29,30]. This highlights the importance of appropriate communication techniques to be adopted by the health workers while working in such communities with high illiteracy. ~~We found the cultural interpretation for the drugs as 'hot', 'heat generating' in this tribal community which is similar to the finding of a study among Vietnamese refugees~~ [31]. We also found many myths and misbeliefs prevalent among the patients and family members affecting treatment compliance. Several other studies also reported similar findings among TB patients [32,33] . In the present study, some patients denied TB diagnosis and were trying to hide the disease. Other studies also reported similar ~~perception~~ of TB patients in different settings. [34,35] Traditional healers are usually a part of the tribal ~~society and the community has full faith in them~~. So, they are consulted first for any ailment in addition to being easily approachable and locally available. We also found this faith in 'Gunia' (traditional healers) among Saharia tribe. A study in South Africa also ~~reported preference for traditional healers~~ among TB patients.[16]

~~The complications and the factors associated with LTFU reinforced the view that only free distribution of the medication is not enough for treatment adherence or the cure.~~ The stigma associated with tuberculosis also plays an important role in treatment compliance. We found that

stigma has strong influence on family and community members. Similar influence of stigma is also reported in various studies conducted in different countries [16,17,28,]. Social support, support from family, friends, neighborhoods, and community are extremely important for TB treatment adherence. Our study revealed that lack of social and family support to TB patients oblige them to stop or interrupt the treatment. Thus, family and community support are essential to promote early diagnosis and complete treatment. Several other studies also reported influence of social/family support including financial and emotional support on treatment adherence. [16,17,28,36].

Overall, the study revealed that multiple factors are responsible for LTFU among *Saharia* tribe and highlighted the importance of better awareness, motivational counseling of patients and family members, and involvement of community in TB diagnosis and treatment. The study also established the need of financial support and nutritional supplements to the TB patients. Thus, social and culturally acceptable interventions along with patient centric health facilities are essential to improve treatment compliance in high endemic areas/community.

Limitations: Few LTFU cases migrated to other districts and states in the search of work and were untraceable where as some demined to participate in the study or to provide a written consent and were excluded from the study. In spite of these limitations, the study provides substantial information about the factors leading to LTFU among the *Saharia* PVTG of Madhya Pradesh. However, the major strength of this study is that it provides information from patients, service providers and family member's perspectives and also from both drug-susceptible (DS) and drug-resistant (DR) TB patients.

***Acknowledgment*:** The authors are thankful to the Director ICMR-NIRTH, Jabalpur for his support and encouragement throughout the study period. Authors are grateful to Dr. Samrdihi Nigam and Dr. Mercy Aparna Lingala for their support in the study. Sincere thanks are due to the district coordinators for carrying out the study in remote tribal villages and all the field staff for their help during the data collection. We are also thankful to the study participants who spared their time and provided valuable information during the study.

REFERENCES

1. Central TB Division, Directorate General of Health Services, Ministry of Health and Family Welfare, Government of India. India TB Report 2018, Government of India, 2018.
2. WHO (2002) An expanded DOTS framework for effective tuberculosis control. WHO/CDS/TB/2002.297. Geneva: World Health Organization. 23 p. Available at: http://whqlibdoc.who.int/hq/2002/WHO_CDS_TB_2002.297. pdf. Accessed:12 October 2006.
3. Munro SA, Lewin SA, Smith HJ, Engel ME, Fretheim A, Volmink J. Patient adherence to tuberculosis treatment: A systematic review of qualitative research. PLoS Med. 2007; 4: e238. doi: 10.1371/journal. pmed.0040238 PMID: 17676945.
4. Mitchison DA (1998) How drug resistance emerges as a result of poor compliance during short course chemotherapy for tuberculosis. Int J Tuberc Lung Dis 2: 10–15.
5. World Health Organization (2013) Definitions and Reporting Framework for Tuber-culosis 2013 revision. WHO/HTM/TB/2013.2 Geneva, Switzerland: WHO.
6. Chang SH, Cataldo JK. A systematic review of global cultural variations in knowledge, attitudes and health responses to tuberculosis stigma. Int J Tuberc Lung Dis. 2014; 18: 168–73. doi: 10.5588/ijtld.13. 0181 PMID: 24429308.
7. Hasker E, Khodjikhanov M, Sayfiddinova S, Rasulova G, Yuldashova U, Uzakova G, et al. Why do tuberculosis patients default in Tashkent City, Uzbekistan? A qualitative study. Int J Tuberc Lung Dis. 2010; 14: 1132–1139. PMID: 20819258.
8. Gler M, Podewils L, Munez N, Galipot M, Quelapio M, Tupasi T (2012) Impact of patient and program factors on default during treatment of multidrug-resistant tub-erculosis. Int J Tuberc Lung Dis 2012 JJul 16(7):955–960. doi: 10.5588/ijtld.11.0502 PMID: 22584124.
9. Kurbatova EV, Taylor A, Gammino VM, Bayona J, Becerra M, Danilovitz M, et al. (2012) Predictors of poor outcomes among patients treated for multidrug-resistant tuber-culosis at DOTS-plus projects, Tuberculosis 92 (2012); 92:397–403. doi: 10.1016/j.tube.2012.06.003 PMID: 22789497.

10. Basai S, Saha KB, Sharma RK, Muniyandi M, Singh N. An overview of Tribal Population in India. Tribal Health Bulletin 2014; 20(special issue) https://nirth.res.in/publications/tribal_health_bulletin/thb_special_issue2014.pdf.
11. IIPS. National Family Health Survey-4 (2015-16), 2017. Mumbai: International Institute for Population Sciences (IIPS).
12. Chakma T, Rao PV, Pall S, Kaushal LS, Datta M, Tiwary RS. Survey of pulmonary tuberculosis in a primitive tribe of Madhya Pradesh. *Indian J Tuberc* 1996; 43: 85–9.
13. Rao VG, Gopi PG, Bhat J, Selvakumar N, Yadav R, Tiwari B, Gadge V, Bhondeley MK, Wares F. Pulmonary tuberculosis: a public health problem amongst the Saharia, a primitive tribe of Madhya Pradesh, Central India. *Int J Inf Dis* 2010; 14 (8):e713–716.
14. Rao VG, Bhat J, Yadav R, Muniyandi M, Sharma R, Bhondeley MK. Pulmonary tuberculosis - a health problem amongst Saharia tribe in Madhya Pradesh. *Indian J Med Res* 2015 May; 141(5): 630–635.
15. Mundra A, Deshmukh PR, Dawale A. Magnitude and determinants of adverse treatment outcomes among tuberculosis patients registered under Revised National Tuberculosis Control Program in a Tuberculosis Unit, Wardha, Central India: A record-based cohort study: Journal of Epidemiology and Global Health 2017; 7: 111–118. http://dx.doi.org/10.1016/j.jegh.2017.02.002.
16. Edginton ME, Sekatane CS, Goldstein SJ (2002) Patients' beliefs: Do they affect tuberculosis control? A study in a rural district of South Africa. Int J Tuberc Lung Dis 6: 1075–1082.
17. Wares DF, Singh S, Acharya AK, Dangi R (2003) Non-adherence to tuberculosis treatment in the eastern Tarai of Nepal. Int J Tuberc Lung Dis 7: 327–335.
18. Bhattacharya S, Singh A. Why tuberculosis control programmes fail? Role of microlevel and macrolevel factors: an analysis from India. BMJ Case Rep. 2017 Apr 21;2017:bcr2017219606. doi: 10.1136/bcr-2017-219606.
19. Sophia Vijay , Prahlad Kumar, Lakbir Singh Chauhan, Balasangameshwara Hanumanthappa Vollepore, Unnikrishnan Pallikkara Kizhakkethil, Sumathi Govinda Rao. Risk Factors Associated With Default Among New Smear Positive TB Patients Treated Under DOTS in India. PLoS One. 2010 Apr 6;5(4):e10043. doi: 10.1371/journal.pone.0010043.

20. Singh V, Jaiswal A, Porter JDH, Ogden JA, Sarin R, et al.(2002) TB control, poverty, and vulnerability in Delhi, India. Trop Med Int Health 7: 693–700.
21. Suhadev M, Thomas BE, Raja Sakthivel M, et al. Alcohol Use Disorders (AUD) among Tuberculosis Patients: A Study from Chennai, South India. PLoS ONE 2011; 6(5): e19485. doi:10.1371/journal.pone.0019485.
22. Bagchi S, Ambe G and Sathiakumar N. Determinants of poor adherence to antituberculosis treatment in Mumbai, India: International Journal of Preventive Medicine 2010; 1(4): 223–232.
23. Santha T, Garg R, Frieden TR, Chandrasekaran V, Subramani R, et al. (2002). Risk factors associated with default, failure and death among tuberculosis patients treated in a DOTS programme in Tiruvallur District, South India, 2000. Int J Tuberc Lung Dis 6(9): 780–788.
24. Duraisamy K, Mrithyunjayan S, Ghosh S, Nair SA, Balakrishnan S, Subramoniapillai J, Oeltmann JE, Moonan PK, Kumar AM. Does Alcohol consumption during multidrug-resistant tuberculosis treatment affect outcome? A population-based study in Kerala, India. Ann Am Thorac Soc. 2014 Jun;11(5):712-8. doi: 10.1513/AnnalsATS.201312-447OC.
25. Al-Hajjaj MS, Al-Khatim IM. High rate of noncompliance with antituberculosis treatment despite a retrieval system: a call for implementation of directly observed therapy in Saudi Arabia. Int J Tuberc Lung Dis 2000;4(4):345–9.
26. Deshmukh RD, Dhande DJ, Sachdeva KS, Sreenicas A, Kumar AMV, Satyanarayna S, et al. Patient and provider Reported Reasons for loss to follow up in MDRTB Treatment: A Qualitative Study from a Drug Resistant TB Center in INDIA. PloS ONE 2015;10(8): e0135802. Doi:10.1371/journal.pone.0135802.
27. Wohlleben J, Makhmudova M, Saidova F, Azamova S, 0Mergenthaler C, Verver S. Risk factors associated with loss to follow-up from tuberculosis treatment in Tajikistan: a case-control study. BMC Infectious Diseases, 2017; 17:543 DOI 10.1186/s12879-017-2655-7.
28. Khan MA, Walley JD, Witter SN, Shah SK, Javeed S (2005) Tuberculosis patient adherence to direct observation: Results of a social study in Pakistan. Health Policy Plan 20: 354–365.23.

29. Dandona R, Dandona L, Mishra A, Dhingra S, Venkatagopalakrishna K, et al. (2004) Utilization of and barriers to public sector tuberculosis services in India. Natl Med J India 17(6): 292–9.;
30. Garrido Mda S, Penna ML, Perez-Porcuna TM, de Souza AB, Marreiro Lda S, Albuquerque BC, Martínez-Espinosa FE, Bührer-Sékula S. Factors associated with tuberculosis treatment default in an endemic area of the Brazilian Amazon: a case control-study. PLoS One. 2012;7(6):e39134. doi: 10.1371/journal.pone.0039134. Epub 2012 Jun 12.
31. Nair D, George A, Chacko K (1997) Tuberculosis in Bombay: New insights from poor urban patients. Health Policy Plan 12: 77–85.
32. Cremers AL, de Laat MM, Kapata N, Gerrets R, Klipstein-Grobusch K, Grobusch MP. Assessing the Consequences of Stigma for Tuberculosis Patients in Urban Zambia. PLoS ONE 2015; 10(3): e0119861. doi:10.1371/journal.pone.0119861.
33. Musuka G, Teveredzi V, Mutenherwa F, Chingombe I, Mapingure M, Musuka et al. Tuberculosis knowledge, misconceptions/myths in adults: findings from Lesotho, Malawi, Namibia and Zambia Demographic Health Surveys (2013-2016). BMC Res Notes 2018;11:778 https://doi.org/10.1186/s13104-018-3884-6.
34. Watkins RE, Plant AJ (2004) Pathways to treatment for tuberculosis in Bali:Patient perspectives. Qual Health Res 14: 691–703.
35. Khan A, Walley J, Newell J, Imdad N (2000) Tuberculosis in Pakistan: Sociocultural constraints and opportunities in treatment. Soc Sci Med 50: 247–254.
36. Martha Priedeman Skiles, Siân L Curtis, Gustavo Angeles , Stephanie Mullen, Tatyana Senik. Evaluating the Impact of Social Support Services on Tuberculosis Treatment Default in Ukraine. PLoS One. 2018 Aug 9;13(8):e0199513. doi: 10.1371/journal.pone.0199513. eCollection 2018.

Table-I : Background characteristics of respondents (N=22)

**Factors**	**Frequency (N=22)**	**Factors**	**Frequency (N=22)**
**Sex**		**Migration within country**	
Males	19	Moved Yes	08
Females	03	Moved No	14
**Age group**		**Addiction**	
18-39	09	Only Alcohol	03
40-59	09	Only Smoking	05
≥60	04	Both	06
**Education**		Never	08
No formal education	18	**Current employment**	
Primary	04	Yes	06
Middle	0	No	16
Higher	0	**Employment before starting treatment but had to quit**	
**Patient category**		Yes	15
New	03	No	07
Previously treated	19	**Prior employment type**	
**Drug resistant status**		Stone quarries	05
Yes	13	Agricultural farming	06
No	09	daily wages labour	11
**Resistant type**		**BMI**	
RR-MDR	09	≥18.5	04
H-mono/Poly	02	≤18.5	18
XDR	02		
**Side effects**			
Yes	18		
No	04		

Table II: Common themes that emerged during in-depth interview with LTFU TB patients, Family members and treatment supporters.

S. No.	Themes		Frequency (N=22)
I	Living condition (Household related)	a) Head of family	Yes -16
		b) House type	Hut/Kutcha-11
		c) Mode of cooking	*Chulla* -21
		d) Place of cooking	Indoor -13
		e) Ventilation	No -12
II.	Personal and life style related	a) Education	Illiterate -17, Primary -4
		b) Alcohol	Yes - 9
		c) Smoking	Yes -11
		d) Myths and misbelief	Yes - 08
III.	Socio-economic factors related	a) Social stigma and discrimination	Social Stigma -5<br>Discrimination - 2
		b) Lack of family and social support	Family support -3<br>Social support - 4
		c) Unemployment and financial constrains	Unemployment -14<br>Finical constrains - 17
		d) Migration for wages	Yes - 8
IV.	Service provider related	a) Behaviour of treatment supporter	Satisfactory-22
		b) Poor counseling	Yes - 07
V.	Medications related	a) Adverse drug and treatment effects	Dizziness -8, Fatigue-8, Vertigo-10, Vomiting-9, Nausea-5, Stomach ache-6
		b) Long duration regimen	Yes - 8
		c) High pill burden	Yes - 8

Figure - I: Web-diagram of Multi factors responsible for loss to follow-up (LTFU) among *Saharia* TB patients

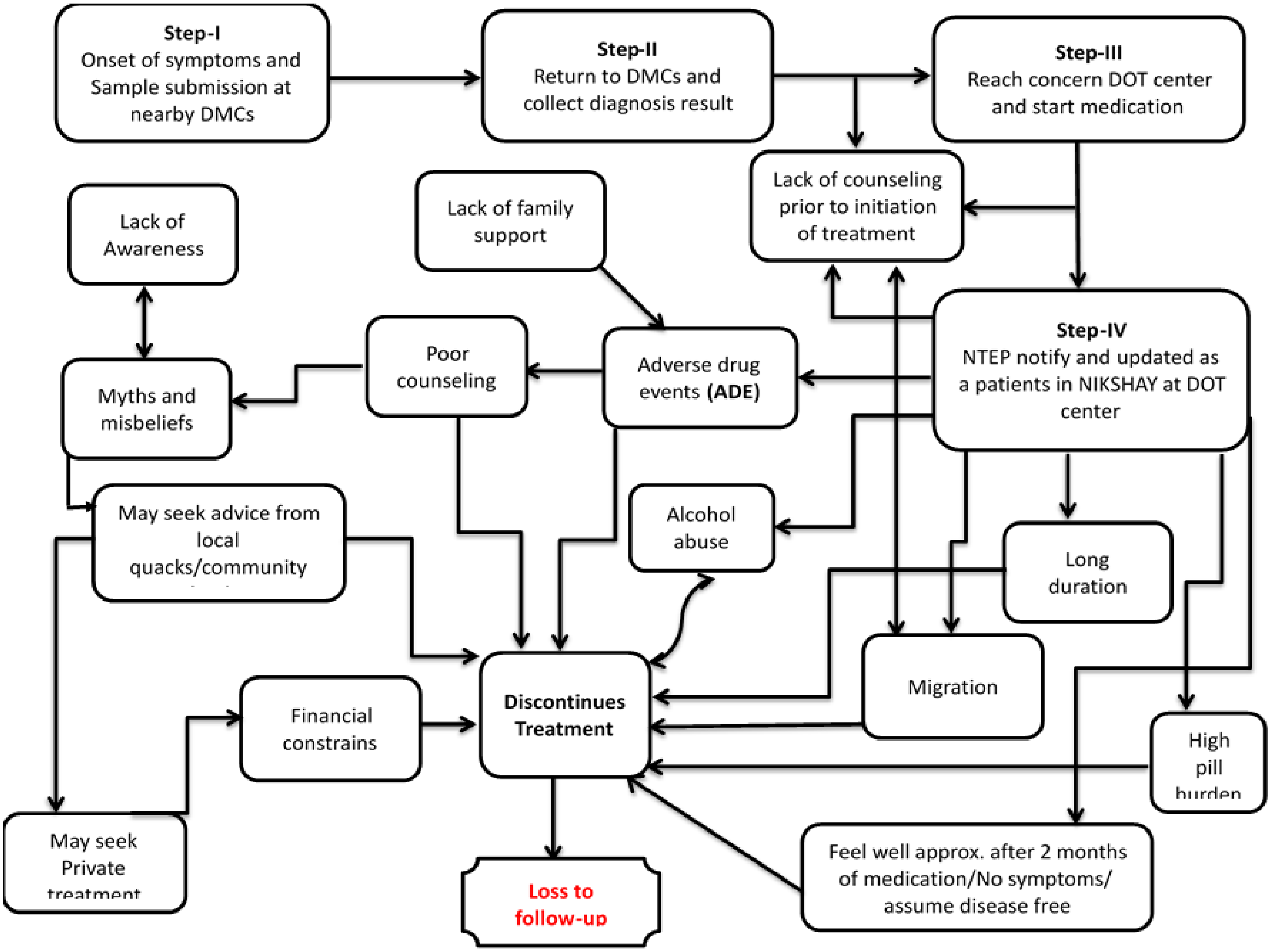

Supplement: Attachment — Submitted filename: PONE-D-20-21794_reviewer.pdf [file pone.0261152.s005.pdf]
