# Supplementary material for: Reasons for loss to follow-up (LTFU) of pulmonary TB (PTB) patients: A qualitative study among Saharia, a particularly vulnerable tribal group of Madhya Pradesh, India
Source: PLoS One. 2021 Dec 23;16(12):e0261152. doi: 10.1371/journal.pone.0261152 (PMC8699669; doi:10.1371/journal.pone.0261152)
Supplement: Attachment — Submitted filename: PONE-D-20-21794_Reviwed.pdf [file pone.0261152.s007.pdf]

PLOS ONE

Reasons for loss to follow-up (LTFU) of pulmonary TB (PTB) patients: A qualitative study among Saharia, a particularly vulnerable tribal group of Madhya Pradesh, India

--Manuscript Draft--

**Manuscript Number:**	PONE-D-20-21794R1
**Article Type:**	Research Article
**Full Title:**	Reasons for loss to follow-up (LTFU) of pulmonary TB (PTB) patients: A qualitative study among Saharia, a particularly vulnerable tribal group of Madhya Pradesh, India
**Short Title:**	Factors leading to Loss to follow up cases of Tuberculosis
**Corresponding Author:**	Jyothi Bhat<br>ICMR-National Institute of Research in Tribal Health<br>Jabalpur, Madhya Pradesh INDIA
**Keywords:**	Lost to follow up, adherence, treatment, Tuberculosis, Saharia
**Abstract:**	<p><p><div class="abstract"> <div><h1 class="heading">Background</h1> <p>Loss to follow up (LTFU) of pulmonary tuberculosis (PTB) patients is a major challenge for TB control. However, there is dearth of information about the factors leading to LTFU among marginalized communities. This study highlights the factors associated with LTFU in Saharia, a tribe of Madhya Pradesh having high tuberculosis (TB) prevalence.</p> <h1 class="heading">Methods</h1> <p>A qualitative study was carried out during January-April 2020 among twenty-two pulmonary TB patients, recorded as LTFU in NIKSHAY, along with 10 treatment supporters and 10 patient’s family members. Semi structured personal interview tools were used to collect the information and interviews were transcribed and inductive content analysis was conducted to originate promising themes.</p> <h1 class="heading">Results</h1> <p>The study explored various social, behavioral factors leadings to loss to follow-up among <i>Saharia</i> PTB patients. Drug side effects, alcoholism, social stigma, lack of awareness to seriousness of disease and poor counseling are the main barricades to treatment adherence in this community.</p> <h1 class="heading">Conclusions</h1> <p>The study highlights the need of addressing the issues related to LTFU during the course of TB treatment. The enhanced efforts of treatment supporter, health staff and Family & community persons are required to motivate and support the patients.</p> </div> </div></p></p>
**Order of Authors:**	Prashant Mishra
	Ravendra K Sharma
	Rajiv Yadav
	VG Rao
	Samridhi Nigam
	Mercy Aparna Lingala
	Jyothi Bhat
**Response to Reviewers:**	We have addressed all the comments from editor and reviewers.<br>Point wise reply file is uploaded.
**Additional Information:**	
**Question**	**Response**
**Financial Disclosure**<br><br>Enter a financial disclosure statement that describes the sources of funding for the work included in this submission. Review the submission guidelines for detailed	The study is financially supported by the Government of Madhya Pradesh (Budget 2210/2017-18/877 dated_27/01/18). However, the funding agency had no role in the study design, data collection, analyses, interpretation of results, reports & manuscript writing and submission to the journal.

Powered by Editorial Manager® and ProduXion Manager® from Aries Systems Corporation

requirements. View published research articles from *PLOS ONE* for specific examples.<br><br>This statement is required for submission and **will appear in the published article** if the submission is accepted. Please make sure it is accurate.<br><br>**Unfunded studies**<br>Enter: *The author(s) received no specific funding for this work.*<br><br>**Funded studies**<br>Enter a statement with the following details:<br>• Initials of the authors who received each award<br>• Grant numbers awarded to each author<br>• The full name of each funder<br>• URL of each funder website<br>• Did the sponsors or funders play any role in the study design, data collection and analysis, decision to publish, or preparation of the manuscript?<br>• **NO** - Include this sentence at the end of your statement: *The funders had no role in study design, data collection and analysis, decision to publish, or preparation of the manuscript.*<br>• **YES** - Specify the role(s) played.<br><br>* typeset	
**Competing Interests**<br><br>Use the instructions below to enter a competing interest statement for this submission. On behalf of all authors, disclose any competing interests that could be perceived to bias this work—acknowledging all financial support and any other relevant financial or non-financial competing interests.<br><br>This statement **will appear in the published article** if the submission is accepted. Please make sure it is accurate. View published research articles from *PLOS ONE* for specific examples.	The authors have declared that no competing interests exist

**NO authors have competing interests**<br><br>Enter: *The authors have declared that no competing interests exist.*<br><br>**Authors with competing interests**<br><br>Enter competing interest details beginning with this statement:<br><br>*I have read the journal's policy and the authors of this manuscript have the following competing interests: [insert competing interests here]*<br><br>* typeset	
**Ethics Statement**<br><br>Enter an ethics statement for this submission. This statement is required if the study involved:<br><br>• Human participants<br>• Human specimens or tissue<br>• Vertebrate animals or cephalopods<br>• Vertebrate embryos or tissues<br>• Field research<br><br>Write "N/A" if the submission does not require an ethics statement.<br><br>General guidance is provided below. Consult the submission guidelines for detailed instructions. **Make sure that all information entered here is included in the Methods section of the manuscript.**	This study was carried out as a part of the main ongoing study "Intensified Tuberculosis Control Project among Saharia of Madhya Pradesh". The ongoing TB control project is approved by Institutional Ethics Committee (IEC) with a reference no. NIRTH/IEC/2273/2016. Participants who were willing to participate and provided written informed consents were enrolled as study participants. ICMR-NIRTH is under Ministry of Health and Family Welfare, Government of India. The main study was approved and supported by the department of health and the department of tribal welfare, Government of Madhya Pradesh. Hence, no separate permission was sought. The manuscript is also approved by institute publication committee (ICMR-NIRTH/PSC/46/2020).

**Format for specific study types**<br><br>**Human Subject Research (involving human participants and/or tissue)**<br>• Give the name of the institutional review board or ethics committee that approved the study<br>• Include the approval number and/or a statement indicating approval of this research<br>• Indicate the form of consent obtained (written/oral) or the reason that consent was not obtained (e.g. the data were analyzed anonymously)<br><br>**Animal Research (involving vertebrate animals, embryos or tissues)**<br>• Provide the name of the Institutional Animal Care and Use Committee (IACUC) or other relevant ethics board that reviewed the study protocol, and indicate whether they approved this research or granted a formal waiver of ethical approval<br>• Include an approval number if one was obtained<br>• If the study involved *non-human primates*, add *additional details* about animal welfare and steps taken to ameliorate suffering<br>• If anesthesia, euthanasia, or any kind of animal sacrifice is part of the study, include briefly which substances and/or methods were applied<br><br>**Field Research**<br><br>Include the following details if this study involves the collection of plant, animal, or other materials from a natural setting:<br>• Field permit number<br>• Name of the institution or relevant body that granted permission	
**Data Availability**<br><br>Authors are required to make all data underlying the findings described fully available, without restriction, and from the time of publication. PLOS allows rare exceptions to address legal and ethical concerns. See the PLOS Data Policy and FAQ for detailed information.	No - some restrictions will apply

Powered by Editorial Manager® and ProduXion Manager® from Aries Systems Corporation

A Data Availability Statement describing where the data can be found is required at submission. Your answers to this question constitute the Data Availability Statement and **will be published in the article**, if accepted.<br><br>**Important:** Stating 'data available on request from the author' is not sufficient. If your data are only available upon request, select 'No' for the first question and explain your exceptional situation in the text box.<br><br>Do the authors confirm that all data underlying the findings described in their manuscript are fully available without restriction?	
**Describe where the data may be found in full sentences. If you are copying our sample text, replace any instances of XXX with the appropriate details.**<br><br>• If the data are **held or will be held in a public repository**, include URLs, accession numbers or DOIs. If this information will only be available after acceptance, indicate this by ticking the box below. For example: *All XXX files are available from the XXX database (accession number(s) XXX, XXX.).*<br>• If the data are all contained **within the manuscript and/or Supporting Information files**, enter the following: *All relevant data are within the manuscript and its Supporting Information files.*<br>• If neither of these applies but you are able to provide **details of access elsewhere**, with or without limitations, please do so. For example:<br><br>*Data cannot be shared publicly because of [XXX]. Data are available from the XXX Institutional Data Access / Ethics Committee (contact via XXX) for researchers who meet the criteria for access to confidential data.*<br><br>*The data underlying the results presented in the study are available from (include the name of the third party*	The manuscript is based on qualitative study. Hence the raw data has names of the participants. So, a formal administrative and ethical clearances is required for sharing raw data/information. The Institutional ethical committee's Member Secretary, Dr.Tapas Chakma, Scientist 'G' may be contacted in this regard (tapas_chakma@rediffmail.com).".

and contact information or URL).<br>• This text is appropriate if the data are owned by a third party and authors do not have permission to share the data.<br><br>* typeset	
Additional data availability information:	

Powered by Editorial Manager® and ProduXion Manager® from Aries Systems Corporation

Manuscript

Click here to access/download;Manuscript;LTFU_Manuscript_R1_PlosONE_F

Reasons for loss to follow-up (LTFU) of pulmonary TB (PTB) patients: A qualitative study among *Saharia*, a particularly vulnerable tribal group of Madhya Pradesh, India

Prashant Mishra[1], Ravendra K Sharma[2], Rajiv Yadav[1], VG Rao[1], Samridhi Nigam[1], Mercy Aparna. Lingala[1] and Jyothi Bhat[1,*]

1. ICMR-National Institute of Research in Tribal Health (NIRTH), Jabalpur, India.
2. ICMR-National Institute of Medical Statistics (NIMS), New Delhi, India

*Corresponding author

Email: drjyothibhat@rediffmail.com (JB)

Abstract

Background

Loss to follow-up (LTFU) of pulmonary tuberculosis (PTB) patients is a significant challenge for TB control. However, there is a dearth of information about the factors leading to LTFU among marginalized communities. This study highlights the factors associated with LTFU in Saharia, a tribe of Madhya Pradesh having high tuberculosis (TB) prevalence.

Methods

A qualitative study was carried out during January-April 2020 among twenty-two pulmonary TB patients, recorded as LTFU in NIKSHAY, along with ten treatment supporters and ten patient's family members. The project team interviewed them using semi-structured personal interview tools to collect their background information, information on the history of anti-tuberculosis treatment, adverse drug events (ADE), social cognitive, behaviors, myths, and misbeliefs. The interviews were transcribed and conducted a thematic analysis to examine underlying themes.

Results

The study explored various social, behavioral factors leading to loss of follow-up among *Saharia* PTB patients. Drug side effects, alcoholism, social stigma, lack of awareness of the disease's seriousness, and inadequate counseling are the main barricades to treatment adherence in this community.

Conclusions

The study highlights the need to address the issues related to LTFU during TB treatment. The enhanced efforts of treatment supporters, health staff, and Family & community persons are required to motivate and support the patients.

Funding

The study was carried out as a sub-study under the ongoing study on Intensified Tuberculosis Control Project among Saharia: A Particularly Vulnerable Tribal Group in Madhya Pradesh. The Government of Madhya Pradesh funded the main study.

Introduction

India is the highest TB burden country globally, accounting for about a quarter of the world's TB cases [1]. Though the Revised National Tuberculosis Control Programme (RNTCP), now National Tuberculosis Elimination Programme (NTEP), has been successfully implemented in India for more than two decades, tuberculosis is still a major challenge in the country, especially in rural and remotes areas. DOTS is the backbone of India's TB elimination program, with treatment compliance as an effective strategy [2]. The NTEP adopted a Directly observed treatment short course (DOTS) as a TB treatment strategy for almost three decades in the country. The same strategy is adopted for both DS and DRTB patients under programmatic conditions using multiple anti-tubercular drugs. The standardized treatment regimens for both drug susceptible TB and drug resistant TB are used under NTEP. The regimens comprising of 4 drugs for 6-9 months (isoniazid, rifampicin, pyrazinamide, ethambutol) are used for drug

susceptible TB and comprising of 6 drugs for (kanamycin, ofloxacin, ethionamide, pyrazinamide, ethambutol, and cycloserine) are used for drug resistant TB for 24 months. The programme has now introduced a shorter MDR TB regimen and all oral longer MDR TB regimen with new drugs like Bedaquiline and Delamanid which can be modified according to DST results. The patients are required to attend the hospital at the end of the intensive as well as continuation phase. During the visits, necessary investigations including sputum examination are performed and the health condition of the patients is reviewed. All these anti-tubercular drugs are known for their various side effects. Though treatment providers are expected to support the patients in treatment continuation, there is no community-based adherence treatment support available to patients in this tribal community. Poor compliance to tuberculosis treatment is a major obstacle for TB elimination programme as it can increase the risk of drug resistance and prolong infectiousness and may result in unfavourable outcomes such as treatment failure, death, and relapse, thus posing a public health threat [3,4]. This is particularly relevant for the tribal population, an underprivileged group of the society usually residing in remote rural areas, thus having poor access to the health delivery system.

Loss to follow up (LTFU) has been defined by the World Health Organization (WHO) as "patient whose treatment was interrupted for two consecutive months or more" [5]. LTFU is a result of several factors such as access to health services, socioeconomic status, literacy level, and beliefs and practices prevalent in the society [6,7]. Several other studies conducted earlier in different settings have also identified many factors responsible for LTFU: illiteracy, poverty, long duration of treatment, many medicines, access to health services, work-related issues, smoking & alcoholism beliefs & practices, etc.[3,8,9]. . However, limited studies on the factors

responsible for LTFU among TB patients are available in India, especially from resource-constrained settings, including remote tribal areas.

Madhya Pradesh (MP) state in central India alone accounts for 14.7% of the country's total tribal population [10] and tribes are consistently having poor health-seeking behavior and access to health services [11]. The Saharia is one of the Particularly Vulnerable Tribal Groups (PVTG) in the state of Madhya Pradesh, central India. Their habitats are located outside the main village called as Saharana. Their economy is primitive and is socio-economically underdeveloped with poor nutritional status. They migrate from one place to other in search of livelihoods [12,13]. Tuberculosis is a key public health problem among them. The studies carried out among this tribe reported a very high prevalence of pulmonary tuberculosis (PTB), 1270 in 1991-92 [14], 1518 in 2008 [15],and 3294 in 2015 [16] per 100,000 in selected *Saharia* areas. A population-based study carried out during 2013 – 2015 also showeda high prevalence of PTB (3003 per 100,000) along with a high proportion of unfavourable treatment outcomes including LTFU among them [17]. The drug resistant TB is an emerging public health problem in the Saharia tribe [18]. Bhat et al. however reported that the rates for drug resistant TB were not different from the national average in this community [19]. The study also reported that most patients with MDR TB in this community were previously treated, patients. There is, however, no information available on LTFU among drug resistant tuberculosis patients and the factors associated with LTFU of TB patients amongst the Saharia tribal population. We, therefore, conducted a qualitative study to obtain a better understanding of TB patients, family members, and treatment provider-related factors for LTFU among the Saharia tribe of Madhya Pradesh, central India.

Methods

Study setting and Population

The study was carried out as a sub-study of the ongoing integrated TB control project (ITCP) in the Saharia population residing in seven districts of Gwalior and Chambal divisions of Madhya Pradesh state in central India. The districts are linked to drug-resistant TB center (DRTBC) Gwalior, Madhya Pradesh. Drug sensitive (DS) TB patients are provided medicine from the nearest TB DOT center by DOTs providers. The drug-resistant (DR) TB patients are treated at the district TB center (DTC) initially during the pre-treatment evaluation period and later receive treatment from the nearest TB DOT center.

Study design and sampling

A qualitative study was carried out to explore the reasons for loss to follow-up (LTFU) among Saharia pulmonary TB (PTB) patients. Thirty-Eight PTB (DS and DR) patients (31 males and seven females) were recorded as LTFU in NIKSHAY from the list of TB patients registered for treatment under the integrated TB control project January-October 2019. The survey team visited all LTFU patients during January-April 2020, and among those, five died, three migrated to other districts, and eight LTFU patients were on re-treatment. Rest all twenty-two LTFU patients (19 males and three females) were successfully interviewed and these include thirteen drug-resistant and nine drug-sensitive pulmonary TB patients. Ten treatment supporters and family members of ten patients were also interviewed. Purposive sampling was conducted to ensure maximum variation in responses of treatment supporters and family members

Data Collection Tool and collection technique

All interviews were carried out using pre-designed open-ended in-depth interview guides developed separately for TB patients, family members, and treatment supporters. These guides were prepared in consultation with the help of clinicians, DOTS providers in the field, and subject experts and social scientists at ICMR-NIRTH, Jabalpur. The semi-structured interview guides include structured questions on their background information, like age, sex, education status, occupation and past/family history of anti-TB treatment (ATT), and open-ended questions related to adverse drug events (ADE), social cognitive, behaviors, myths and misbelieve that determine adherence and factors associated with loss to follow-up.

All in-depth interviews were conducted in local regional language (a dialect of *Hindi*) by project scientists (PS) and district coordinators (DC's) of the project who possessed a postgraduate degree either in Life Science, or Public Health, or Social Science disciplines and are trained in TB diagnosis and treatment guidelines. The researchers have been working with the study population for more than one year and are familiar with the local dialect of the Saharia tribe. But, none of the researchers belonged to Saharia tribal community. The in-depth interviews were carried out during the researchers' routine field monitoring visits. All study participants were interviewed in isolation at a place convenient to them and interview sessions were also audio-recorded with the prior permission of participants.

Data analysis

The audio recordings of participant's responses were initially transcribed in the Hindi language, later translated into the English language by project research scientists. The transcripts were read by two researchers in the field and codes were developed. All these transcripts were also read and audio recordings were heard by a senior social scientist at ICMR-NIRTH, Jabalpur to ensure

that the experiences of the participants were accurately captured and reflected in the inductive codes. The transcripts were coded and categorized into different themes and sub-themes relevant to the study objective. A thematic framework was developed to examine the relationship between themes and understand the various reasons for loss to follow-up, data was reviewed to identify the perspectives of different types of respondents that have been selected to ensure maximum variation to understand similarities and differences between two groups. And also between the various respondents for triangulating the findings or differences. Some direct verbatim quotes that showed important responses under each themeare also presented in the manuscript.

Ethical considerations

This study was carried out as a part of the main ongoing study, “Intensified Tuberculosis Control Project among Saharia of Madhya Pradesh” approved and supported by the department of health and the department of tribal welfare, Government of Madhya Pradesh. The ongoing TB control project is approved by Institutional Ethics Committee (IEC) with reference no. NIRTH/IEC/2273/2016. All the participants were briefed about the objectives of this study, and were enrolled as study participants after obtaining informed written consent. The study participants were not involved in designing the research protocol and interview guides, data collection, analysis, and dissemination of outcomes. ICMR-NIRTH is under Ministry of Health and Family Welfare, Government of India. The main study was approved and supported by the department of health and the department of tribal welfare, Government of Madhya Pradesh. Hence, no separate permission was sought. The institute publication committee also approved the manuscript (ICMR-NIRTH/PSC/46/2020).

Results

Socio-demographic profile and living conditions of participants

The study participants comprised 22 TB patients reported as LTFU patients in NIKSHAY, aged 25 to 70, including 19 male and three female patients, all belonging to the Saharia tribal community. Among the LTFU patients, thirteen patients were drug-resistant - 2 H-Mono resistant, 9 Rifampicin resistant or RR-MDR and 2 were XDR (Table 1).

Table 1: Background information of patients (N=22)

Factors	Sex	Age	Previous Treatment history	DR Status	Resistant Type
Patient No.1	M	30	Yes	Yes	RR
Patient No.2	F	38	Yes	Yes	H-Mono
Patient No.3	M	45	Yes	No	
Patient No.4	M	36	Yes	No	
Patient No.5	M	25	Yes	No	
Patient No.6	M	45	Yes	No	
Patient No.7	M	60	Yes	No	
Patient No.8	M	45	Yes	Yes	RR
Patient No.9	M	50	Yes	Yes	RR
Patient No.10	M	34	New	Yes	RR
Patient No.11	M	70	Yes	Yes	H-Mono
Patient No.12	M	32	Yes	No	
Patient No.13	M	45	New	No	
Patient No.14	M	60	Yes	Yes	RR
Patient No.15	M	40	Yes	No	
Patient No.16	M	50	Yes	Yes	RR
Patient No.17	M	35	Yes	Yes	RR
Patient No.18	M	32	Yes	Yes	RR
Patient No.19	F	30	New	No	
Patient No.20	M	42	Yes	Yes	XDR
Patient No.21	F	65	Yes	Yes	RR
Patient No.22	M	45	Yes	Yes	XDR

The majority (18/22) of the patients were underweight (BMI <18.5) and previously treated (19/22). Most of the respondents are illiterate and work as manual labours at stone quarries, agriculture fields, road/house constructions, or other development projects, which is their primary livelihood source. They also migrate to nearby districts and states in search of work/jobs and remain outside of their villages for a substantial period of the year. Most of their houses are Kutcha houses (walls and/or roof made of mud/stone) or huts (thatched walls and/or ceiling) with a single room and mostly without ventilation. The same room is also used for cooking. The primary fuel used for cooking is wood or crop residuals producing a lot of smoke, one of the significant risk factors of pulmonary tuberculosis (Table 2).

The in-depth interviews of participants identified several factors leading to LTFU in the Saharia community. The themes developed during the analysis of in-depth interviews andimportant findings are summarized below -

Table 2: Common themes that emerged during in-depth interviews of LTFU TB patients

**S. No.**	**Themes**	**Sub-Themes**	**Drug-Sensitive (N=09)**	**Drug-resistant (N= 13)**
I	Living condition **(**Household related**)**	a) Head of family	Yes -07	Yes- 09
		b) House type	Hut/Kutcha- 02	Hut/Kutcha-09
		c) Mode of cooking	*Chulla* - 08	*Chulla* -13
		d) Place of cooking	Indoor - 04	Indoor – 09
		e) Ventilation	No - 03	No – 09
II.	Personal and lifestyle related	a) Education	Illiterate -06, Primary -03	Illiterate -11, Primary -02
		b) Alcohol	Yes - 04	Yes – 05
		c) Smoking	Yes -04	Yes -07
		d) Myths and misbelief	Yes - 02	Yes-06

III.	Socio-economic factors related	a) Social stigma and discrimination	Social Stigma -1<br>Discrimination - 01	Social Stigma -04<br>Discrimination -01
		b) Lack of family and social support	Family support -01<br>Social support – 01	Family support -02<br>Social support – 03
		c) Unemployment and financial constraints	Unemployment -04<br>Finical constraints - 07	Unemployment -10<br>Finical constraints - 11
		d) Migration for wages	Yes - 04	Yes -04
IV.	Service provider related	a) Behaviour of treatment supporter	Satisfactory-09	Satisfactory-13
		b) Poor counseling	Yes - 04	Yes – 03
V.	Medications related	a) Adverse drug and treatment effects	Dizziness - 02, Fatigue-04,<br>Vertigo-03,<br>Vomiting-02,<br>Nausea-01,<br>Stomach ache-03	Dizziness -06,<br>Fatigue-04,<br>Vertigo-07,<br>Vomiting-07,<br>Nausea-04,<br>Stomach ache-03
		b) Long duration regimen	Yes - 0	Yes – 08
		c) High pill burden	Yes - 0	Yes – 08

Adverse drug effects

The adverse drug effects (ADE) of medications have been a significant barricade for treatment adherence in the study population. The majority of patients (eighteen out of twenty-two patients) reported some kind of adverse drug effects, Commonly reported ADE of TB medication were vomiting, severe headache, vertigo, stomach ache, nausea, and haemoptysis. The adverse drug effects were reported by both drug-sensitive and drug-resistant TB patients.

A 30 years old DR male patient reported – *"When I used to take medicines, I used to get blood out of my mouth, I used to vomit and had trouble in breathing! So, I quit the medicine."* [Patient 1]. Similarly, a 45 years old DS male reported *"Whenever I took medicine, there was severe pain in the stomach, a burning sensation in the urine, the medicine used to heat a lot! So, I quit the medicine."* [Patient 3].

Treatment providers and patient's family members also reported drug side effects as a reason for treatment discontinuation and LTUF among patients.

A 42 years old family member (patient's wife) told - *"He was not taking medicines because he was unhappy with medicine, he had a burning sensation in his stomach and nervousness"* [Family member 4,] whereas

a 25 years old male treatment supporter reported - *"Patients do not take medicine properly because medicine causes heat in the stomach, and vomiting and diarrhea occur, so they quit medicine after few days, and they approach local Gunia (traditional healer) or private practitioner)"* [Treatment supporter 6].

However, more patients perceived adverse drug effects as the important reason for discontinuing treatment compared to the treatment providers and family members.

High Pill burden and drug quality

In addition to adverse drug effects, a high pill burden is also an important reason for LTFU in TB patients in this community. The daily regimen of 9-11 pills with injection made it difficult for some patients to complete treatment mainly among drug-resistant patients. A drug-resistant patient reported –

"Though I had to take 8-10 pills every day, I was taking these pills but I was also asked to take injections. As I was already weak, I used to get dizziness after injection and go into slumber" [Patient 8, Male, 45 years].

However, few patients also perceive that apart from the quantity, the poor quality of drugs is also a reason for loss to follow-ups and the drug's poor effects.

A 34 years old drug-resistant patient complained of the quality of the drugs and reported- *"The pills were not of good quality, I had to take 8 pills, it was troublesome to take these medicines regularly"* [Patient 10].

The high pill burden and fear of injection among TB patients is also recognized as a reason for LTFU by treatment provides. As one treatment supporter reported "*Patients give up medicine as they have to take many pills, and due to the fear of injections* [Treatment supporter 08, Male, 25 years].

Work-related and financial constraint

Work-associated problems are also identified as one of the reasons for the discontinuation of the treatment among patients. Fourteen patients reported that they had to quit their job to start the treatment. Some patients mentioned difficulties in carrying out their works after taking medicines. These Saharia TB patients are economically poor and they mainly work as daily wage agriculture or a manual labourer.

A 35 years old male DR- patient reported *"I used to go to stone quarry work. Since I got sick and I am unable to go to work. Earlier, I used to earn Rs. 50-100 daily, now that too has gone"* [Patients 17,]. While few others mentioned financial constraints in continuing their treatment,

and because of their poverty they have to work despite their sickness. A 42 years old male XDR patient reported-

"*As I had taken payment in advance and was working away from my home, I could not go to my house, so I quit medicine"* [Patient 20].

When they are too weak to continue their work, their family members substitute them so that their family could sustain them. As a 30 years old DS male patient reported "*I used to earn Rs 250-300 daily, but I can no longer go to work due to my illness. So, now, my wife goes to work" [Patient 03]. A family member also reported-*

"Since he is sick, I and my son go to earn outside, I want him to get well soon and start earning so that we do not have to worry. But he has stopped taking medicine due to feeling dizziness after medication. We are troubled by working outside" [Family member 4, Female, 42 years].

Alcohol abuse and smoking

Habits like alcohol use and tobacco consumption are prevalent among the Saharia community. During the interviews, many patients reported alcohol consumption and smoking as a factor for not adhering to treatment. These were also written by treatment supporters (DOTS providers) and the patient's family members. However, after getting the disease, many of them also stopped smoking and alcohol consumption.

A 40 years old male DS patient mentioned *"Earlier I used to smoke beedi and drink liquor, but now I have given up since I got sick"* [Patient 15]. Another 50 years old DR male reported *"Earlier, I used to drink liquor, but now I have not consumed liquor from last 2-3 years. Now, I smoke only 2-4 Beedi per day, I have been smoking since I was 10-12 years old."* [Patient 9]. But few patients do not stop alcohol consumption and some prefer to stop taking medicine over alcohol. A 35 years old female family member (patient's wife) reported - "*My husband is not*

taking medicine because he drinks liquor. I have explained to him many times about the bad effects of liquor, but he does not listen to me. He is still suffering from disease, he coughs all the day" [Family member, 2].

However, few patients also consume or continue to consume alcohol to suppress the symptoms of disease and side effects of the medicine. A 45 years old DS-patient reported *"Yes, I drink liquor, I drink to suppress cough and I take approximately 200 ml at a time"* [Patient 3].

A treatment supporter also mentioned, "*Many patients drink liquor and smoke (Beedis) during the treatment, due to which the medicine cause more heat/burning sensation (adhik garmi karti hai) and they give up the medicine instead of stopping drinking and smoking"* [Treatment supporter 4, Male, 29 Year].

Stigma and family support

Social stigma, discouragement, and low-income family support are also reported as significant causes and barriers to not completing the TB treatment. The fears of discrimination by their family members directly affect the activities to promote adherence and TB treatment outcomes. During the interview sessions, few TB patients reported that they did not want health care workers to visit their homes for counseling. They did not want to visit their local or block and district level DOT center due to fear of disclosure of their disease status.

A 38 years old DR female patient reported - *"When my TB treatment started, I was very ashamed to tell my neighbor and the villagers. I do not know why, but there was always a fear; I did not tell anyone my treatment was for what"* [Patient 2]. A 30 years old male DR patient also mentioned "*When I got sick, no one from my family was willing to accompany me to the hospital. Only my wife was willing to come, but I did not take her to the hospital because she had never visited a hospital, she does not know anything about hospitals"* [Patient 1]. Few family members

also informed that they prefer to keep the patient outside of the house to avoid infection to other family members. A 35 years old family member (patient's wife) told - *"We live separately, though he is my husband. He lives in a hut outside the house, because it is a communicable disease"* [Family member 2].

Myths and misbelieve

Lack of awareness about the seriousness of the disease and its treatment also influenced treatment adherence adversely. Many patients who did not complete their treatment had myths and misbeliefs associated with the TB disease and treatment. Some patients do not trust diagnosis and continue to believe that they do not have TB disease. So, they are reluctant to start treatment and stop taking medicine after a few days/ weeks of treatment initiation. A 45 years old male DS patient reported –

"*I do not have any disease, so why should I take medicine."* [Patient 13].

However, some also believed that taking medicine or anti-TB treatment will further deteriorate their health.

A 50 years old male DR patient reported, *"I would have not been able to sit again if I had taken these pills and would have died. People in my family also used to get upset and used to say why I should take such pills"* [Patient 9].

A few of them also perceived that different patients get different anti-TB medicines. One who received good medicines he/she completes treatment, while others who don't receive good medicines had a side effect.

A 45 years old male DS patient revealed - "*My younger brother had the same disease (TB). He also took medicine but the medicines he was taking were different. My medicine caused stomach*

aches and a burning sensation in the urine. My medicine was not good, he got better medicines" [Patient 6]. Whereas, some patients also expressed peer pressure to stop medicines. Sixty years old male DR-patient reported –

"I was taking medicine for a long time and when I started getting blood in the cough, my neighbors and villagers asked me to stop taking too many medicines. So, I quit medicine" [Patient 14].

During interviews, it was also observed that patients, care providers, and family members also correlate misfortunes with TB disease or its treatment. A 38 years old DR female patient who lost her child during the treatment reported –

"*When I was taking medicines, my son died. I was very much disheartened and left the medicines. When my son is no more, then for whom should I take medicines?"* [Patient 2].

Challenges and barriers (Representative's quotations)

Treatment supporters and patient's family members were also interviewed to identify the challenges and barriers leading to LTFU. Some of them mentioned that long duration of treatment, alcoholism, and smoking are important causes of LTFU. As reported by one treatment supporter –

"As far as I have experienced, the medicines have adverse effects (garmi karti hai) and the duration of treatment is prolonged. So, patients take medicine for one or two months and then they quit medicine" [Treatment supporter 1, Male, 20 years].

Another treatment supporter reported -

"Many patients drink liquor daily and get drunk and do not take medicines regularly. Subsequently, they stop medicine [Treatment supporter 4, Male, 29 years].

Some patients stop the medicine after taking one-two months when symptoms get subsided, thinking that they are disease-free and thus do not take a full course of treatment. A treatment supporter reported –

"*When patients take proper medicine regularly, they start to feel better within a month, and they start thinking that they have recovered from the disease and quit the medicines"* [Treatment supporter 2, Male, 20 years]. Similarly, another treatment supporter revealed -

"After taking medicines for 1-2 months, patient's health improves and he thinks that he is cured, and start avoiding medicines, and finally stop the medicine" [Treatment supporter 3, Male, 35 years].

The important quotations of the drug-sensitive and drug-resistant study participants are given below (Table 3) -

Table 3: Theme-wise important quotations of the drug-sensitive and drug-resistant study participants

**Drug Sensitive participants**	**Drug Resistant participants**
**Adverse drug effects**	
"Whenever I took medicine, there was severe pain in the stomach, a burning sensation in the urine, the medicine used to heat a lot! So, I quit the medicine." [Patient 3, Male, 45 years].<br><br>*"I used to vomit after taking medicine, So, I quit this medicine."* [Patient 05, Male, 25 years].	*"When I used to take medicines, I used to get blood out of my mouth, I used to vomit and had trouble in breathing! So, I quit the medicine."* [Patient 1, Male, 30 years].<br><br>*"When I used to take pills, I had severe pain in the stomach, vomiting, and diarrhea, there was a burning sensation in the stomach, and also felt dizziness and nervousness. So, I quit this medicine."* [Patient 10, Male, 34 years].
**High Pill burden and drug quality**	
	"Though I had to take 8-10 pills every day, I was taking these pills but I was also asked to take injections. As I was already weak, I used to get

	dizziness after injection and go into slumber" [Patient 8, Male, 45 years].<br><br>*"The pills were not of good quality, I had to take 8 pills, it was troublesome to take these medicines regularly"* [Patient 10, Male, 34 years].
**Work-related and financial constraint**	
"I used to go to daily wages work. Since I got sick and I am unable to go to work. Earlier, I used to earn Rs. 200-250 daily, now that too is gone otherwise, I would have earned 6-7 thousand in a month" [Patients 04, Male, 35 years].<br><br>*"I used to earn Rs. 250-300 daily; I can no longer go to work due to illness. Now wife goes to work"* [Patients 03, Male, 35 years].	*"I used to go to stone quarry work. Since I got sick and I am unable to go to work. Earlier, I used to earn Rs. 50-100 daily, now that too is gone"* [Patients 17, Male, 35 years].<br><br>*"When I was taking medicine, I used to get dizziness and I was afraid that I wouldn't get my wages"* [Patient 16, Male, 50 years].<br><br>"As I have taken payment in advance *and working away from my home, I cannot go to my house, so I quit medicine"* [Patient 20, Male, 42 years].
**Alcohol abuse and smoking**	
"Yes, I drink liquor, I drink to suppress cough and I take approximately 200 ml at a time." [Patient 3, Male, 45 years].<br><br>*"Earlier I used to smoke beedi and drink liquor, but now I have given up since I got sick" [Patient 15, Male, 40 years].*	*"Earlier, I used to drink liquor, but now I have* not consumed liquor for the last 2-3 years. *Now, I smoke only 2-4 Beedi per day, I have been smoking since I was 10-12 years old." [Patient 9, Male, 50 years].*<br><br>*"Earlier I used to smoke Ganja (Marijuana) and I smoked for 4-5 years, but now I have given up since last 3-4 months" [Patient 10, Male, 34 years].*
**Stigma and family support**	
"When my TB treatment started, my wife started living separately from me, I have to live in a hut (Jhopdi) outside the home and my food is also prepared separately" [Patient 04, Male, 34 years].	*"When my TB treatment started, I was very ashamed to tell my neighbor and the villagers. I do not know why, but there was always a fear; I did not tell anyone my treatment was for what"* [Patient 2, Female, 38 years].<br><br>"*When I got sick, no one from my family was*

	willing to accompany me to the hospital. Only my wife was willing to come, but I did not take her to the hospital because she had never visited a hospital, she does not know anything" [Patient 1, Male, 30 years].
**Myths and misbelieves**	
"*I do not have any disease, so why should I take medicine."* [Patient 13, Male, 45 years].<br><br>"*My younger brother had the same disease (TB). He also took medicine, but the medicines he was taking were different. My medicine caused stomach aches and a burning sensation in the urine. My medicine was not good, he got better medicines"* [Patient 6, Male, 45 Year].	*"I would have not been able to sit again if I had taken these pills and would have died. People in my family also used to get upset and used to say why I should take such pills"* [Patient 9, Male, 50 years].<br><br>*"I was taking medicine for* a long time and when I started getting blood in the cough, my neighbors and villagers asked me to stop taking too much *medicine. So, I quit medicine"* [Patient 14, Male, 60 years].<br><br>"*When I was taking medicines, my son died; I was very much disheartened and left the medicines. When my son is no more, then for whom should I take medicines?"* [Patient 2, Female, 38 years].

Discussion

This is the first reported qualitative study that provides insight into the causes of LTFU of TB patients among the Saharia- a PVTG in Madhya Pradesh with a very high TB burden. India's National Tuberculosis Elimination Programme reported 4% LTFU for the TB patients notified in 2018 [20]. A recent study from central India reported 8.6% LTFU among TB patients registered under NTEP [21]. Our study among the Saharia tribe found a high proportion of unfavorable treatment outcomes including a higher rate of post-treatment mortality [22] and LTFU (15.3%) among Saharia TB patients (unpublished data). The low socio-economic status, frequent

migration to other areas, under-nutrition, poor access to health facilities, and inadequate reach of the health system due to remotely located Saharia habitats might be contributing to higher LTFU in this community. The study focused on in-depth analyses of narrative data from patients, treatment supporters, and patient's family members.

We found that patients' judgment of the severity of their fatigue, dizziness, vomiting, and high pill burdens also led to LTFU among the Saharia. The adverse drug events (ADE) and high pill burden of TB regimen promote discontinuation of treatment or patients shift to private TB treatment, especially among DR patients. The poor awareness of the drug's possible side effects and the absence of counseling before initiating medications contribute to the LTFU. . Without proper counseling, patients drift out of the system and discontinue the treatment. Several other studies also reported similar findings among TB patients [3,21, 23-26]. In the community setting, particularly in remote tribal areas, when ADE occurs, patients need advice and counseling but they hardly find any health staff nearby to support them. So, they are compelled to approach local practitioners and traditional healers readily available in the hamlet/locality. Our findings show that the patients were not fully aware of the possible side effects of drugs and were not aware of how to cope with these adverse effects, particularly in the intensive phase of treatment. Other studies have also reported similar findings in different regions [23,24,27]. The possible reason for higher ADE among the Saharia tribe could be the high prevalence of undernutrition among them. Patients with poor nutritional status are reported to have a higher risk of hepatotoxicity which is one of the important ADE due to anti-TB drugs, thus contributing to LTFU [28,29]. The BMI rates are lower in this tribe, and adequate nutrition deprivations likely to lead to severe reactions like vomiting and nausea, thus promoting discontinuation of TB

treatment among patients. . The pre-treatment counseling and active surveillance of patients' adverse reactions by the health staff are crucial in improving treatment compliance.

Our work shows that alcohol consumption during treatment is a significant barrier to treatment compliance in the Saharia tribe. The findings are consistent with previous investigations. Alcoholism has been reported as a significant factor of patient non-compliance or adverse treatment outcomes among tuberculosis patients receiving DOTS treatment [26,30-33]. Because of these findings, the history of alcoholism before starting treatment would help identify potential defaulters. We also found smoking leading to treatment discontinuation in the present study. Similar findings were observed among TB patients receiving standard TB regimen in different settings [31,34]. The Saharia tribe is one of Madhya Pradesh's marginalized communities, and they depend on daily work for their livelihood. Most of the LTFU patients are either primary earners or are in economic active ages, work primarily on the daily wages. The financial constraints and personal reasons related to work, i.e., fear of losing a job and migration within the country for the job, are key factors responsible for losing follow-up and not completing the treatment. Several other studies also reported work and financial constraints as the possible reasons for treatment discontinuation [23,30,35-37].

The Saharia tribe is a particularly vulnerable tribal group of Madhya Pradesh with extensive illiteracy. Our study also showed that most of the study participants are illiterate. Thus, incorrect knowledge or myths, and misconceptions about TB disease and its medications and treatments are common among patients and family members. The low literacy could also be why their incorrect knowledge about TB disease has been reported in several studies [26,38,39]. . This highlights the importance of appropriate communication techniques to be adopted by the health workers while working in such communities with high illiteracy. We found the cultural

interpretation for the drugs as ‘hot’, ‘heat generating’ in this tribal community, which is similar to finding a study among Vietnamese refugees [40]. We also found many myths and misbeliefs prevalent among the patients and family members affecting treatment compliance. Several other studies also reported similar findings among TB patients [41,42]. In the present study, some patients denied TB diagnosis and were trying to hide the disease. Other studies also reported similar perceptions of TB patients in different settings [43,44]. Traditional healers are usually a part of the tribal society, and the community has complete faith in them. So, they are consulted first for any ailment in addition to being easily approachable and locally available. We also found this faith in ‘Gunia’ (traditional healers) among the Saharia tribe. A study in South Africa also reported a preference for traditional healers among TB patients [23].

The complications and the factors associated with LTFU reinforced the view that only the medication's free distribution is not enough for treatment adherence or the cure. The stigma associated with tuberculosis also plays an important role in treatment compliance. We found that stigma has a strong influence on family and community members. A similar influence of stigma is also reported in various studies conducted in different countries [23,24,37],. Social support, support from family, friends, neighborhoods, and community are extremely important for TB treatment adherence. Our study revealed that lack of social and family support to TB patients obliges them to stop or interrupt the treatment. Thus, family and community support are essential to promote early diagnosis and complete treatment. Several other studies also reported social/family support influence, including financial and emotional support, on treatment adherence [23,24,37,45].

Overall, the study revealed that multiple factors are responsible for LTFU among the Saharia tribe and highlighted the importance of better awareness, motivational counseling of patients and

family members, and community involvement in TB diagnosis and treatment. The study also established the need for financial support and nutritional supplements for TB patients. The enhanced efforts of treatment supporters, health staff, and family & community persons are required to motivate and support the patients. Further research to devise appropriate strategies to improve treatment compliance should be undertaken as part of TB control in high endemic areas/communities.

Limitations

The study is based on an in-depth interview of 22 Saharia tribe TB patients and provides substantial information about the factors leading to LTFU among the *Saharia* PVTG of Madhya Pradesh, but one needs to be cautious while generalizing the findings.

The major strength of this study is that it provides information from patients, service providers, and family member's perspectives and also from both drug-susceptible (DS) and drug-resistant (DR) TB patients.

Acknowledgment

The authors are thankful to the Director ICMR-NIRTH, Jabalpur for his support and encouragement throughout the study period. Sincere thanks are due to the district coordinators of the project for carrying out the study in remote tribal villages and all the field staff for their help during the data collection. We are also thankful to the study participants who spared their time and provided valuable information during the study.

References

1. Central TB Division, Directorate General of Health Services, Ministry of Health and Family Welfare, Government of India. India TB Report 2018, Government of India, 2018.
2. World Health Organization. An expanded DOTS framework for effective tuberculosis control. WHO/CDS/TB/2002.297. Geneva: World Health Organization. 2002. Available at: http://whqlibdoc.who.int/hq/2002/WHO_CDS_TB_2002.297.
3. Munro SA, Lewin SA, Smith HJ, Engel ME, Fretheim A, Volmink J. Patient adherence to tuberculosis treatment: A systematic review of qualitative research. PLoS Med. 2007; 4: e238. doi: 10.1371/journal. pmed.0040238 PMID: 17676945.
4. Mitchison DA. How drug resistance emerges as a result of poor compliance during short course chemotherapy for tuberculosis. Int J Tuberc Lung Dis. 1998; 2: 10–15.
5. World Health Organization. Definitions and Reporting Framework for Tuberculosis 2013 revision. WHO/HTM/TB/2013.2 Geneva, Switzerland: WHO.2013.
6. Chang SH, Cataldo JK. A systematic review of global cultural variations in knowledge, attitudes and health responses to tuberculosis stigma. Int J Tuberc Lung Dis. 2014; 18: 168–173. doi: 10.5588/ijtld.13. 0181 PMID: 24429308.
7. Hasker E, Khodjikhanov M, Sayfiddinova S, Rasulova G, Yuldashova U, Uzakova G, et al. Why do tuberculosis patients default in Tashkent City, Uzbekistan? A qualitative study. Int J Tuberc Lung Dis. 2010; 14: 1132–1139. PMID: 20819258.
8. Gler M, Podewils L, Munez N, Galipot M, Quelapio M, Tupasi T. Impact of patient and program factors on default during treatment of multidrug-resistant tuberculosis. Int J Tuberc Lung Dis. 2012; 16(7):955–960. doi: 10.5588/ijtld.11.0502 PMID: 22584124.

9. Kurbatova EV, Taylor A, Gammino VM, Bayona J, Becerra M, Danilovitz M, et al. Predictors of poor outcomes among patients treated for multidrug-resistant tuberculosis at DOTS-plus projects, Tuberculosis. 2012; 92(5):397–403. doi: 10.1016/j.tube.2012.06.003 PMID: 22789497.
10. Basai S, Saha KB, Sharma RK, Muniyandi M, Singh N. An overview of Tribal Population in India. Tribal Health Bulletin. 2014; 20(special issue) https://nirth.res.in/publications/tribal_health_bulletin/thb_special_issue2014.pdf.
11. IIPS. National Family Health Survey-4 (2015-16), Mumbai: International Institute for Population Sciences (IIPS). 2017.
12. Sati VP. Sahariya Tribe: Society, Culture, Economy and Habitation. Ann Nat Sci. 2015;1(1): 26-31.
13. Rajak J. Nutritional and Socio-Economic Status of Saharia Tribes In Madhya Pradesh. Int J Hum Soc Sci. 2016; 6(1):79-85.
14. Chakma T, Rao PV, Pall S, Kaushal LS, Datta M, Tiwary RS. Survey of pulmonary tuberculosis in a primitive tribe of Madhya Pradesh. Indian J Tuberc. 1996; 43: 85–89.
15. Rao VG, Gopi PG, Bhat J, Selvakumar N, Yadav R, Tiwari B, et al. Pulmonary tuberculosis: a public health problem amongst the Saharia, a primitive tribe of Madhya Pradesh, Central India. Int J Inf Dis. 2010; 14 (8):e713–716.
16. Rao VG, Bhat J, Yadav R, Muniyandi M, Sharma R, Bhondeley MK. Pulmonary tuberculosis - a health problem amongst the Saharia tribe in Madhya Pradesh. Indian J Med Res. 2015; 141(5): 630–635.

17. Rao VG, Bhat J, Yadav R, Sharma RK and Muniyandi M. Declining tuberculosis prevalence in Saharia, a particularly vulnerable tribal community in Central India: evidences for action. BMC Infect Dis. 2019;19(1):180. doi: 10.1186/s12879-019-3815-8.
18. Prakash R, Kumar D, Gupta VK, Jain S, Chauhan DS, Tiwari PK, et al. Status of multidrug-resistant tuberculosis (MDR-TB) among the Sahariya tribe of North Central India. J Infect Public Health. 2016;9(3):289-297. doi: 10.1016/j.jiph.2015.10.008. Epub 2016 Jan 6.PMID: 26775848.
19. Bhat J, Rao VG, Yadav R, Muniyandi M, Sharma R, Karfarma C, et al. Situation of drug resistant tuberculosis in Saharia tribe of central India. Indian J Med Res. 2015;141(5):636-639. doi: 10.4103/0971-5916.159562.PMID: 26139782.
20. Central TB Division. India TB Report, 2020. Annual Report. Ministry of Health and Family Welfare. Government of India.2020.
21. Mundra A, Deshmukh PR, Dawale A. Magnitude and determinants of adverse treatment outcomes among tuberculosis patients registered under Revised National Tuberculosis Control Program in a Tuberculosis Unit, Wardha, Central India: A record-based cohort study: J Epidemiol Glob Health. 2017; 7: 111–118. http://dx.doi.org/10.1016/j.jegh.2017.02.002.
22. Rao VG, Muniyandi M, Sharma RK, Yadav R, Bhat Jyothi. Long term survival of patients treated for tuberculosis: a population-based longitudinal study in a resource poor setting. Trop Med Int Health (In press),
23. Edginton ME, Sekatane CS, Goldstein SJ. Patients' beliefs: Do they affect tuberculosis control? A study in a rural district of South Africa. Int J Tuberc Lung Dis. 2002; 6: 1075–1082.

24. Wares DF, Singh S, Acharya AK, Dangi R. Non-adherence to tuberculosis treatment in the eastern Tarai of Nepal. Int J Tuberc Lung Dis. 2003; 7: 327–335.

25. Bhattacharya S, Singh A. Why tuberculosis control programmes fail? Role of microlevel and macrolevel factors: an analysis from India. BMJ Case Rep. 2017 Apr 21;2017:bcr2017219606. doi: 10.1136/bcr-2017-219606.

26. Vijay S, Kumar P, Chauhan LS, Vollepore BH, Kizhakkethil UP, Rao SG. . Risk Factors Associated With Default Among New Smear Positive TB Patients Treated Under DOTS in India. PLoS One. 2010; ;5(4):e10043. doi: 10.1371/journal.pone.0010043.

27. Singh V, Jaiswal A, Porter JDH, Ogden JA, Sarin R, Sharma PP, et al. TB control, poverty, and vulnerability in Delhi, India. Trop Med Int Health. 2002; 7: 693–700.

28. Padmapriyadarsini C, Shobana M, Lakshmi M, Beena T, Swaminathan S. Undernutrition & tuberculosis in India: Situation analysis & the way forward. Indian J Med Res. 2016 Jul;144(1):11-20. doi: 10.4103/0971-5916.193278.PMID: 27834321.

29. Lv X, Tang S, Xia Y, Wang X, Yuan Y, Hu D, et al. Adverse reactions due to directly observed treatment strategy therapy in Chinese tuberculosis patients: a prospective study. PLoS One. 2013;8(6):e65037. doi:10.1371/journal.pone.0065037. PMID: 23750225.

30. Suhadev M, Thomas BE, Raja Sakthivel M, Murugesan P, Chandrasekaran V, Charles N, et al. Alcohol Use Disorders (AUD) among Tuberculosis Patients: A Study from Chennai, South India. PLoS ONE. 2011; 6(5): e19485. doi:10.1371/journal.pone.0019485.

31. Bagchi S, Ambe G and Sathiakumar N. Determinants of poor adherence to antituberculosis treatment in Mumbai, India: Int J Prev Med. 2010; 1(4): 223–232.

32. Santha T, Garg R, Frieden TR, Chandrasekaran V, Subramani R, Gopi PG, et al. Risk factors associated with default, failure, and death among tuberculosis patients treated in a DOTS programme in Tiruvallur District, South India, 2000. Int J Tuberc Lung Dis. 2002; 6(9): 780–788.

33. Duraisamy K, Mrithyunjayan S, Ghosh S, Nair SA, Balakrishnan S, Subramoniapillai J, et al. Does Alcohol consumption during multidrugresistant tuberculosis treatment affect outco me? A population-based study in Kerala, India. Ann Am Thorac Soc. 2014;;11(5):712-8. doi: 10.1513/AnnalsATS.201312-447OC.

34. Al-Hajjaj MS, Al-Khatim IM. High rate of noncompliance with antituberculosis treatment despite a retrieval system: a call for implementation of directly observed therapy in Saudi Arabia. Int J Tuberc Lung Dis. 2000;4(4):345–349.

35. Deshmukh RD, Dhande DJ, Sachdeva KS, Sreenicas A, Kumar AMV, Satyanarayna S, et al. Patient and provider Reported Reasons for loss to follow up in MDRTB Treatment: A Qualitative Study from a Drug Resistant TB Center in INDIA. PloS ONE. 2015;10(8): e0135802. Doi:10.1371/journal.pone.0135802.

36. Wohlleben J, Makhmudova M, Saidova F, Azamova S, Mergenthaler C, Verver S. Risk factors associated with loss to follow-up from tuberculosis treatment in Tajikistan: a case-control study. BMC Infectious Diseases. 2017; 17:543 DOI 10.1186/s12879-017-2655-7.

37. Khan MA, Walley JD, Witter SN, Shah SK, Javeed S. Tuberculosis patient adherence to direct observation: Results of a social study in Pakistan. Health Policy Plan. 2005; 20: 354–365. doi: 10.1093/heapol/czi047. Epub 2005 Sep 23.

38. Dandona R, Dandona L, Mishra A, Dhingra S, Venkatagopalakrishna K, Chauhan LS. Utilization of and barriers to public sector tuberculosis services in India. Natl Med J India. 2004; 17(6): 292–299.
39. Garrido Mda S, Penna ML, Perez-Porcuna TM, de Souza AB, Marreiro Lda S, Albuquerque BC, et al. Factors associated with tuberculosis treatment default in an endemic area of the Brazilian Amazon: a case control-study. PLoS One. 2012;7(6):e39134. doi: 10.1371/journal.pone.0039134. Epub 2012 Jun 12.
40. Nair D, George A, Chacko K. Tuberculosis in Bombay: New insights from poor urban patients. Health Policy Plan. 1997; 12: 77–85.
41. Cremers AL, de Laat MM, Kapata N, Gerrets R, Klipstein-Grobusch K, Grobusch MP. Assessing the Consequences of Stigma for Tuberculosis Patients in Urban Zambia. PLoS ONE. 2015; 10(3): e0119861. doi:10.1371/journal.pone.0119861.
42. Musuka G, Teveredzi V, Mutenherwa F, Chingombe I, Mapingure M. Tuberculosis knowledge, misconceptions/myths in adults: findings from Lesotho, Malawi, Namibia and Zambia Demographic Health Surveys (2013-2016). BMC Res Notes. 2018;11:778 https://doi.org/10.1186/s13104-018-3884-6.
43. Watkins RE, Plant AJ. Pathways to treatment for tuberculosis in Bali: Patient perspectives. Qual Health Res. 2004; 14: 691–703.
44. Khan A, Walley J, Newell J, Imdad N. Tuberculosis in Pakistan: Sociocultural constraints and opportunities in treatment. Soc Sci Med. 2000;50: 247–254.
45. Priedeman SM, Curtis SL, Angeles G, Mullen S, Senik T. Evaluating the Impact of Social Support Services on Tuberculosis Treatment Default in Ukraine. PLoS One. 2018; 13(8):e0199513. doi: 10.1371/journal.pone.0199513. eCollection 2018.

Click here to access/download
Supporting Information
DOTS provider.doc

Click here to access/download
Supporting Information
DOTS provider interview guide.doc

Click here to access/download
Supporting Information
Family member interview guide.doc

Click here to access/download
Supporting Information
LTFU Patient Interview guide.doc

~~Factors leading to~~Reasons for loss to follow-up (LTFU) of pulmonary TB (PTB) patients: A qualitative ~~S~~study among *Saharia*, a particularly vulnerable tribal group of Madhya Pradesh, India

Prashant Mishra[1], Ravendra K Sharma[2], Rajiv Yadav[1], VG Rao[1], Samridhi Nigam[1], Mercy Aparna. Lingala[1] and Jyothi Bhat[1,*]~~,~~

1. ICMR-National Institute of Research in Tribal Health (NIRTH), Jabalpur, India.
2. ICMR-National Institute of Medical Statistics (NIMS), New Delhi, India

*Corresponding author~~:~~

~~Dr. Jyothi Bhat, M.D.~~
~~ICMR-National Institute of Research in Tribal Health~~
~~Nagpur Road~~
~~P.O. Garha~~

Formatted: Font: 18 pt
Formatted: Font: 18 pt
Formatted: Font: 18 pt
Formatted: Font: 18 pt
Formatted: Font: 18 pt
Formatted: Font: Times New Roman, 18 pt
Formatted: Superscript
Formatted: Superscript
Formatted: Superscript
Formatted: Superscript
Formatted: Superscript
Formatted: Superscript
Formatted: Font: Not Italic
Formatted: Numbered + Level: 1 + Numbering Style: 1, 2, 3, … + Start at: 1 + Alignment: Left + Aligned at: 0.25" + Indent at: 0.5"
Formatted: Font: Not Italic
Formatted: Font: Not Italic
Formatted: Font: Not Italic, Not Superscript/ Subscript
Formatted: Font: Not Italic

~~Jabalpur 482003~~
~~Phone: +91 761 2370800~~
Email: drjyothibhat@rediffmail.com (JB)

Abstract

Background~~:~~

Loss to ~~follow up~~Follow-up (LTFU) of pulmonary tuberculosis (PTB) patients is a ~~major~~ significant challenge for TB control. However, there is the dearth of information about the factors leading to LTFU among marginalized communities. This study highlights the factors associated with LTFU in Saharia, a tribe of Madhya Pradesh having high tuberculosis (TB) prevalence.

Methods~~:~~

A qualitative study was carried out during January-April 2020 among twenty-two pulmonary TB patients, recorded as LTFU in NIKSHAY, along with ~~10~~ ten treatment supporters and ~~10~~ ten patient's family members. The project team interviewed them using ~~S~~semi~~-~~-structured personal interview tools to collect their background information, information on history of anti-tuberculosis treatment, adverse drug events (ADE), social cognitive, behaviors, myths and misbeliefs. ~~were used to collect the information, and~~ The interviews were transcribed and conducted thematic ~~inductive content~~ analysis ~~was conducted~~ to ~~originate promising~~examine underlying themes.

Results~~:~~

Formatted: Font: 18 pt

Formatted: Font: 14 pt

Formatted: Line spacing: Double

Formatted: Font: 14 pt

Formatted: Line spacing: Double

Formatted: Font: 14 pt

The study explored various social, behavioral factors leadings to loss to of follow-up among *Saharia* PTB patients. Drug side effects, alcoholism, social stigma, lack of awareness to of the disease's seriousness, of disease and poor inadequate counseling are the main barricades to treatment adherence in this community.

Formatted: Line spacing: Double

Conclusions:

Formatted: Font: 14 pt

The study highlights the need of to addressing the issues related to LTFU during the course of TB treatment. The enhanced efforts of treatment supporters, health staff and Family & community persons are required to motivate and support the patients.

Formatted: Line spacing: Double

Funding:

Formatted: Font: 14 pt

The study was carried out as a sub-study under the ongoing study on Intensified Tuberculosis Control Project among Saharia: A Particularly Vulnerable Tribal Group in Madhya Pradesh. The Government of Madhya Pradesh The main study is funded the main study.by the Government of Madhya Pradesh.

Formatted: Line spacing: Double

Introduction

India is the highest TB burden country globally, accounting ~~in the world accounting~~ for about a quarter of the world's TB cases [1].~~1~~ Though the Revised National Tuberculosis Control Programme (RNTCP), now National Tuberculosis Elimination Programme (NTEP), has been successfully implemented in India for more than two decades, tuberculosis is still a major challenge in the country, especially in rural and remotes areas. DOTS is ~~a~~ the back~~-~~bone of India's TB elimination program~~me~~, with treatment compliance as ~~one of the major~~an effective strategy [2].~~2~~ The NTEP adopted Directly observed treatment short course (DOTS) as TB treatment strategy for almost three decades in the country. The same strategy is adopted for both DS and DRTB patients under programmatic conditions using multiple anti-tubercular drugs. The standardized treatment regimens for both drug susceptible TB and drug resistant TB are used under NTEP. The regimens comprising of 4 drugs for 6-9 months (isoniazid, rifampicin, pyrazinamide, ethambutol) are used for drug susceptible TB and comprising of 6 drugs for (kanamycin, ofloxacin, ethionamide, pyrazinamide, ethambutol, and cycloserine) are used for drug resistant TB for 24 months. The programme has now introduced shorter MDR TB regimen and all oral longer MDR TB regimen with new drugs like Bedaquiline and Delamanid which can be modified according to DST results. The patients are required to attend the hospital at the end of the intensive as well as continuation phase. During the visits, necessary investigations including sputum examination are performed and the health condition of the patients is reviewed. All these anti-tubercular drugs are known for various side effects. Though treatment providers are expected to support the patients in treatment continuation, there is no community-based adherence treatment support available to patients in this tribal community. Poor compliance to

Formatted: Font: Times New Roman
Formatted: Font: 18 pt
Formatted: Font: (Default) Times New Roman, 12 pt, Font color: Auto
Formatted: Font: Times New Roman
Formatted: Line spacing: Double
Formatted: Font: (Default) Times New Roman, 12 pt
Formatted: Font: (Default) Times New Roman, 12 pt
Formatted: Font: (Default) Times New Roman, 12 pt, Font color: Auto, Pattern: Clear
Formatted: Font: (Default) Times New Roman, 12 pt
Formatted: Font: (Default) Times New Roman, 12 pt, Font color: Auto, Pattern: Clear
Formatted: Font: (Default) Times New Roman, 12 pt
Formatted: Font: (Default) Times New Roman, 12 pt, Font color: Auto, Pattern: Clear
Formatted: Font: (Default) Times New Roman, 12 pt
Formatted: Font: (Default) Times New Roman, 12 pt, Font color: Auto, Pattern: Clear
Formatted: Font: (Default) Times New Roman, 12 pt
Formatted: Font: (Default) Times New Roman, 12 pt, Font color: Auto, Pattern: Clear
Formatted: Font: (Default) Times New Roman, 12 pt
Formatted: Font: (Default) Times New Roman, 12 pt, Not Highlight
Formatted: Font: (Default) Times New Roman, 12 pt, Not Highlight
Formatted: Not Highlight
Formatted: Not Highlight
Formatted: Font: (Default) Times New Roman, 12 pt, Not Highlight
Formatted: Font: (Default) Times New Roman, 12 pt
Formatted: Font: (Default) Times New Roman, 12 pt
Formatted: Font color: Auto

tuberculosis treatment is a major obstacle for TB elimination programme as it can increase the risk of drug resistance and prolong infectiousness and may result in unfavourable outcomes such as treatment failure, death and relapse, thus posing a public health threat [3,4].[3,4] This is particularly relevant for the tribal population, an underprivileged group of the society usually residing usually in remote rural areas, thus having a poor access to the health delivery system.

Loss to follow up (LTFU) has been defined by the World Health Organization (WHO) as “patient whose treatment was interrupted for two consecutive months or more” [5][5]. LTFU is a result of several factors such as access to health services, socioeconomic status, literacy level and beliefs and practices prevalent in the society [6,7].[6,7] Several other studies conducted earlier in different settings have also identified many factors responsible for LTFU: illiteracy, poverty, long duration of treatment, many medicines, access to health services, work-related issues, smoking & alcoholism beliefs & practices, etc.[3,8,9]. Several other studies conducted earlier in different settings have also identified many factors responsible for LTFU such as illiteracy, poverty, long duration of treatment, large number of medicines, access to health services, work related issues, smoking & alcoholism, beliefs & practices etc. [3,8,9]. However, limited studies on the factors responsible for LTFU among TB patients are available in India, especially from resource-constrained settings, including remote tribal areas. India especially from resource-constrained settings including remote tribal areas.

Madhya Pradesh (MP) state in central India alone accounts for 14.7% of the country’s total tribal population [10] and tribes are consistently having poor health-seeking country’s total tribal population[10] and tribes are consistently having poor health seeking behavior and access to the health-services [11].[11] The Saharia is one of the Particularly Vulnerable Tribal Groups (PVTG)

Formatted: Justified, Line spacing: Double

Formatted: Font: (Default) Times New Roman, 12 pt

Formatted: Font: (Default) Times New Roman, 12 pt

Formatted: Font: (Default) Times New Roman, 12 pt

Formatted: Font color: Black

Formatted: Justified, Line spacing: Double

Formatted: Font: (Default) Times New Roman, 12 pt

Formatted: Font: (Default) Times New Roman, 12 pt

Formatted: Font: (Default) Times New Roman, 12 pt

in the state of Madhya Pradesh, central India. Their habitats are located outside the main village called as Saharana. Their economy is primitive and are socio-economically underdeveloped with poor nutritional status. They migrate from one place to other in search of livelihoods [12,13]. Tuberculosis is a key public health problem among *Saharia* tribe – a Particularly Vulnerable Tribal Group (PVTG) of Madhya Pradesh them. The studies carried out among this tribe reported a very high prevalence of pulmonary tuberculosis (PTB), 1270 in 1991-92 [14][12], 1518 in 2008 [15] [13] and 3294 in 2015 [16][14] per 100,000 in selected *Saharia* areas. A population-based study carried out during 2013 – 2015 also showed high prevalence PTB (3003 per 100,000) along with high proportion of unfavourable treatment outcomes including LTFU among them [17]. The drug resistant TB is an emerging public health problem in Saharia tribe [18]. Bhat et al. however reported that the rates for drug resistant TB were not different from national average in this community [19]. The study also reported that most patients with MDR TB in this community were previously treated patients. There is however, no information available on LTFU among drug resistant tuberculosis patients and the factors associated with LTFU of TB patients amongst the Saharia tribal population. We, therefore, conducted a qualitative study to obtain a better understanding of TB patients, family members, and treatment provider-related factors for LTFU among the Saharia tribe of Madhya Pradesh, central India.

information available on the factors associated with LTFU of TB patients amongst Saharia tribal population.

Formatted: Font: Not Italic

Formatted: Font: (Default) Times New Roman, 12 pt, Font color: Auto

Formatted: Font: (Default) Times New Roman, 12 pt

Formatted: Font: (Default) Times New Roman, 12 pt

Formatted: Font: (Default) Times New Roman, 12 pt, Font color: Black

Formatted: Line spacing: Double

Formatted: Font color: Black

Formatted: Justified, Line spacing: Double

We therefore conducted a qualitative study to obtain better understanding of TB patients, family members and treatment provider related factors for LTFU among Saharia tribe of Madhya Pradesh, central India.

Methods

Study setting and Population

The study was carried out as a sub-study of the ongoing integrated TB control project (ITCP) in the Saharia population residing in seven districts of Gwalior and Chambal divisions of Madhya Pradesh state in central India. The districts are linked to drug-resistant TB center (DRTBC) Gwalior, Madhya Pradesh. Drug sensitive (DS) TB patients are provided medicine from the nearest TB DOT center by DOTs providers. The drug-resistant (DR) TB patients are treated at the district TB center (DTC) initially during the pre-treatment evaluation period and later receive treatment from the nearest TB DOT center.

The study was carried out as sub-study of the ongoing integrated TB control project in *Saharia* population residing in seven districts of Gwalior and Chambal divisions of Madhya Pradesh state in central India. The districts are linked to drug resistant TB center (DRTBC) Gwalior, Madhya Pradesh. Drug sensitive (DS) TB patients are provided medicine through DOT providers and linked to the nearest TB DOT center, whereas, drug resistant (DR) TB patients are treated at district TB center (DTC) initially during pre-treatment evaluation period and later receive treatment from the nearest TB DOT center.

Study design and sampling

Formatted: Justified
Formatted: Font: (Default) Times New Roman, 12 pt, Not Bold, Font color: Auto
Formatted: Line spacing: 1.5 lines, Adjust space between Latin and Asian text, Adjust space between Asian text and numbers, Pattern: Clear (White)
Formatted: Font: 18 pt
Formatted: Font: 14 pt, Not Italic
Formatted: Font: 14 pt, Not Italic
Formatted: Font: (Default) Times New Roman, 12 pt
Formatted: Justified, Line spacing: Double
Formatted: Font: (Default) Times New Roman, 12 pt
Formatted: Line spacing: Double
Formatted: Font: 14 pt, Not Italic

A qualitative study was carried out to explore the reasons for loss to follow-up (LTFU) among Saharia pulmonary TB (PTB) patients. Thirty-Eight PTB (DS and DR) patients (31 males and seven females) were recorded as LTFU in NIKSHAY from the list of TB patients registered for treatment under the integrated TB control project January-October 2019. The survey team visited all LTFU patients during January-April 2020, and among those, five ~~were found dead~~died, three migrated to other districts, and eight LTFU patients were on re-treatment. Rest all twenty-two LTFU patients (19 males and three females) were successfully interviewed and these include thirteen drug-resistant and nine drug-sensitive pulmonary TB patients.~~,~~ Ten treatment supporters and family members of ten patients were also interviewed. Purposive sampling was conducted to ensure maximum variation in responses of treatment supporters and family members

~~Thirty Eight PTB (DS and DR) patients (31 males and 7 females)were recorded as LTFU in NIKSHAY, from the list of TB patients registered for treatment under integrated TB control project during January-October 2019. All LTFU patients were visited by survey team during January-April 2020 and among those five were found dead, three migrated to other districts and eight LTFU patients were on re-treatment. Rest all twenty two LTFU patients (19 males and 3 females) were successfully interviewed. Ten treatment supporters and ten patients' family members were also interviewed. Purposive sampling was used to ensure variety of patients' family members and treatment supporters and to capture the maximum variation in responses of treatment supporters and family members. All qualitative in-depth interviews were performed using semi-structured interview guide.~~

Formatted: Font color: Black

Formatted: Font: (Default) Times New Roman, 12 pt

Formatted: Font: (Default) Times New Roman, 12 pt

Formatted: Font: (Default) Times New Roman, 12 pt

Formatted: Font: (Default) Times New Roman, 12 pt

Formatted: Font color: Black

Formatted: Line spacing: Double

Data Collection Tool and collection technique

Formatted: Font: 14 pt, Not Italic

All interviews were carried out using pre-designed open-ended in-depth interview guides developed separately for TB patients, family members, and treatment supporters. These guides were prepared in consultation with the help of clinicians, DOTS providers in the field, and subject experts and social scientists at ICMR-NIRTH, Jabalpur. The semi-structured interview guides include structured questions on their background information, like age, sex, education status, occupation and past/family history of anti-TB treatment (ATT), and open-ended questions related to adverse drug events (ADE), social cognitive, behaviors, myths and misbelieve that determine adherence and factors associated with loss to follow-up.

Formatted: Line spacing: Double

All in-depth interviews were conducted in local regional language (a dialect of *Hindi*) by project scientists (PS) and district coordinators (DC's) of the project who possessed a postgraduate degree either in Life Science, or Public Health, or Social Science disciplines and are trained in TB diagnosis and treatment guidelines. The researchers have been working with the study population for more than one year and are familiar with the local dialect of the Saharia tribe. But, none of the researchers belonged to Saharia tribal community. The in-depth interviews were carried out during the researchers' routine field monitoring visits. All study participants were interviewed in isolation at a place convenient to them and interview sessions were also audio-recorded with the prior permission of participants.

Formatted: Not Highlight

Data ~~collection and~~ analysis

Formatted: Font: 14 pt, Not Italic

~~In-depth interviews were conducted in local, regional language (a dialect of *Hindi*) by project scientists (PS) and district coordinators (DC's) of the project who were trained in TB diagnosis and treatment guidelines. The semi- structured interview tools for patients, treatment supporters~~

Formatted: Justified, Line spacing: Double

~~and patient's family members include their background information, information on history of anti tuberculosis treatment TB patient's previous history of anti-tuberculosis treatment (ATT), adverse drug events (ADE), social cognitive, behaviors, myths and misbeliefs that determine adherence and factors associated with loss to follow up. All study participants were interviewed in the isolation at a place convenient to them and their audio recorded~~ The audio recordings of participant's responses were initially transcribed in the Hindi language, later translated into ~~in~~ the English language by project research scientists. The transcripts were read by two researchers in the field and codes were developed independantly. All these transcripts were also read and audio recordings were heard by a senior social scientist at ICMR-NIRTH, Jabalpur to ensure that the experiences of the participants were accurately captured and reflected in the inductive codes. The transcripts were coded and categorized into different themes and sub-themes relevant to the study objective. A thematic framework was developed to examine the relationship between themes and understand the various reasons for loss to follow-up, data was reviewed to identify the perspectives of different types of respondents that have been selected to ensure maximum variation to understand similarities and differences between two groups. Also, the between the various respondents for triangulating findings or difference.

~~The codes and themes were developed alongside with data collection.~~ Some direct verbatim quotes that showed important responses under each theme ~~/ factors~~ are also presented in the manuscript.

Ethical considerations

This study was carried out as a part of the main ongoing study, "Intensified Tuberculosis Control Project among Saharia of Madhya Pradesh" approved and supported by the department of health

Formatted: Font: (Default) Times New Roman, 12 pt

Formatted: Font: Font color: Black, Pattern: Clear (White)

Formatted: Line spacing: Double

Formatted: Line spacing: Double, Don't adjust space between Latin and Asian text, Don't adjust space between Asian text and numbers

Formatted: Font color: Black

Formatted: Font: 14 pt, Not Italic

and the department of tribal welfare, Government of Madhya Pradesh. The ongoing TB control project is approved by Institutional Ethics Committee (IEC) with a reference no. NIRTH/IEC/2273/2016. All the ~~P~~participants were briefed about the objectives of this study, ~~who were willing to participate and provided~~ and were enrolled as study participants after obtaining informed written ~~informed~~ consent. ~~s were enrolled as study participants~~. The study participants were not involved in designing the research protocol and interview guides, data collection, analysis, and dissemination of outcomes. ICMR-NIRTH is under Ministry of Health and Family Welfare, Government of India. The main study was approved and supported by the department of health and the department of tribal welfare, Government of Madhya Pradesh. Hence, no separate permission was sought~~-~~. The institute publication committee also approved the manuscript ~~The manuscript is also approved by institute publication committee~~ (ICMR-NIRTH/PSC/46/2020).

Results

Socio-demographic profile and living conditions of participants

The study participants comprised 22 TB patients reported as LTFU patients in NIKSHAY, aged 25 to 70, including 19 male and three female patients, all belonging to the Saharia tribal community. Among the LTFU patients, thirteen patients were drug-resistant - 2 H-Mono resistant, 9 Rifampicin resistant or RR-MDR and 2 were XDR (Table 1).

Table 1: Background information of patients (N=22)

Factors	Sex	Age	Previous Treatment history	DR Status	Resistant Type
Patient No.1	M	30	Yes	Yes	RR
Patient No.2	F	38	Yes	Yes	H-Mono
Patient No.3	M	45	Yes	No	

Formatted: Font: (Default) Times New Roman, 12 pt

Formatted: Font: (Default) Times New Roman, 12 pt, Not Bold

Formatted: Font: Font color: Black

Formatted: Font: 18 pt

Formatted: Font: 14 pt, Not Italic

Formatted: Font: (Default) Times New Roman, 12 pt

Formatted: Justified, Line spacing: Double

Formatted: Font: (Default) Times New Roman, 12 pt

Formatted: Centered

Formatted Table

Patient No.4	M	36	Yes	No	
Patient No.5	M	25	Yes	No	
Patient No.6	M	45	Yes	No	
Patient No.7	M	60	Yes	No	
Patient No.8	M	45	Yes	Yes	RR
Patient No.9	M	50	Yes	Yes	RR
Patient No.10	M	34	New	Yes	RR
Patient No.11	M	70	Yes	Yes	H-Mono
Patient No.12	M	32	Yes	No	
Patient No.13	M	45	New	No	
Patient No.14	M	60	Yes	Yes	RR
Patient No.15	M	40	Yes	No	
Patient No.16	M	50	Yes	Yes	RR
Patient No.17	M	35	Yes	Yes	RR
Patient No.18	M	32	Yes	Yes	RR
Patient No.19	F	30	New	No	
Patient No.20	M	42	Yes	Yes	XDR
Patient No.21	F	65	Yes	Yes	RR
Patient No.22	M	45	Yes	Yes	XDR

The majority (18/22) of the patients were underweight (BMI <18.5) and previously treated (19/22). Most of the respondents are illiterate and work as manual labours at stone quarries, agriculture fields, road/house constructions, or other development projects, which is their primary livelihood source. They also migrate to nearby districts and states in search of work/jobs and remain outside of their villages for a substantial The study participants comprised 22 TB patients reported as LTFU patients in NIKSHAY, aged 25 to 70 years. This includes 19 male and 3 female patients, and all belonging to *Saharia* tribal community. Among the LTFU patients, thirteen patients were drug resistant – 2 H-Mono resistant, 9 Rifampicin resistant or RR-MDR and 2 were XDR. Majority (18/22) of the patients were underweight (BMI <18.5) and previously treated (19/22). Most of respondents are illiterate and work as manual labours at stone quarries, agriculture fields and road/house constructions or other development projects which is their main source of livelihood. They also migrate to nearby districts and states in search of work/jobs and

Formatted: Justified, Line spacing: Double

Formatted: Font: (Default) Times New Roman, 12 pt

~~remain outside of their villages for a substantial~~ period of the year ~~(Table I)~~. Most of their houses are Kutcha houses (walls and/or roof made of mud/stone) or huts (thatched walls and/or ceiling) with a single room and mostly without ventilation. The same room is also used for cooking. The primary fuel used for cooking is wood or crop residuals producing a lot of smoke, one of the significant risk factors of pulmonary tuberculosis (Table 2).

The in-depth interviews of participants identified several factors leading to LTFU in the Saharia community. The themes developed during the analysis of in-depth interviews and important findings are summarized ~~in Table~~ ~~, and~~ ~~described~~ below ~~.~~

Table 2: Common themes that emerged during in-depth interviews of LTFU TB patients

**S. No.**	**Themes**	**Sub-Themes**	**Drug-Sensitive (N=09)**	**Drug-resistant (N= 13)**
I	Living condition (Household related)	a) Head of family	Yes -07	Yes- 09
		b) House type	Hut/Kutcha- 02	Hut/Kutcha-09
		c) Mode of cooking	*Chulla* - 08	*Chulla* -13
		d) Place of cooking	Indoor - 04	Indoor – 09
		e) Ventilation	No - 03	No – 09
II.	Personal and lifestyle related	a) Education	Illiterate -06, Primary -03	Illiterate -11, Primary -02
		b) Alcohol	Yes - 04	Yes – 05
		c) Smoking	Yes -04	Yes -07
		d) Myths and misbelief	Yes - 02	Yes-06
III.	Socio-economic factors related	a) Social stigma and discrimination	Social Stigma -1 Discrimination - 01	Social Stigma -04 Discrimination -01
		b) Lack of family and social support	Family support -01 Social support – 01	Family support -02 Social support – 03
		c) Unemployment and	Unemployment -04	Unemployment -10

Formatted: Font: (Default) Times New Roman, 12 pt
Formatted: Font: (Default) Times New Roman, 12 pt
Formatted: Font: (Default) Times New Roman, 12 pt
Formatted: Font: (Default) Times New Roman, 12 pt
Formatted: Font: (Default) Times New Roman, 12 pt
Formatted: Font: (Default) Times New Roman, 12 pt
Formatted: Font: (Default) Times New Roman, 12 pt
Formatted: Centered
Formatted Table

		financial constraints	Finical constraints - 07	Finical constraints - 11
		d) Migration for wages	Yes - 04	Yes -04
IV.	Service provider related	a) Behaviour of treatment supporter	Satisfactory-09	Satisfactory-13
		b) Poor counseling	Yes - 04	Yes – 03
V.	Medications related	a) Adverse drug and treatment effects	Dizziness - 02, Fatigue-04, Vertigo-03, Vomiting-02, Nausea-01, Stomach ache-03	Dizziness -06, Fatigue-04, Vertigo-07, Vomiting-07, Nausea-04, Stomach ache-03
		b) Long duration regimen	Yes - 0	Yes – 08
		c) High pill burden	Yes - 0	Yes – 08

Formatted: Font: (Default) Times New Roman, 12 pt

~~period of the year (Table I). Most of their houses are Kutcha houses (walls and/or roof made of mud/stone) or huts (thatched walls and/or roof) with single room and mostly without any ventilation. The same room is also used for cooking and main fuel used for cooking is wood or crop residuals producing lot of smoke which is one of the major risk factor of pulmonary tuberculosis (PTB)(Table I).~~

Formatted: Line spacing: Double

~~The in-depth interviews of participants identified a number of factors leadings to LTFU in the *Saharia* community. Themes that are developed during analysis of in-depth interviews are summarized in Table II and important verbatim are described below~~

Formatted: Indent: First line: 0"

Formatted: Font: Not Italic

Adverse drug effects

Formatted: Font: 14 pt, Not Italic

The adverse drug effects (ADE) of medications have been a significant barricade for treatment adherence in the study population. The majority of patients (eighteen Oout of twenty-two patients) reported some kind of adverse drug effects, Commonly reported ADE of TB medication were eighteen reported adverse drug effects as a significant barricade for treatment adherence. All patients, treatment providers and patient's family members reported drug's side effects such as vomiting, severe headache, vertigo, stomach ache, nausea and haemoptysis. The adverse drug effects were reported by both drug-sensitive and drug-resistant TB patients.

Formatted: Line spacing: Double

A 30 years old DR male patient reported – *"When I used to take medicines, I used to get blood out of my mouth, I used to vomit and had trouble in breathing! So, I quit the medicine."* [Patient 1]. Similarly, a 45 years old DS male reported *"Whenever I took medicine, there was severe pain in the stomach, a burning sensation in the urine, the medicine used to heat a lot! So, I quit the medicine."* [Patient 3].

Treatment providers and patient's family members also reported drug side effects as a reason for treatment discontinuation and LTUF among patients.

However, relatively patients reported more adverse drug effects as an important reason for discontinuing the treatment compared to treatment providers and family members.

Formatted: Font: (Default) Times New Roman, 12 pt, Font color: Auto

Formatted: Line spacing: Double

A 30 years old male reported – *"When I used to take medicines, I used to get blood out of my mouth, I used to vomit and had trouble in breathing! So, I quit the medicine."* [Patient 1, Male, 30 years].

~~Another 45 years old male reported~~ ~~*"Whenever I took medicine, there was a severe pain in the stomach, a burning sensation in the urine, the medicine used to heat a lot! So, I quit the medicine."*~~ ~~[Patient 3, Male, 45 years].~~

~~The burning sensation and diarrhea were also commonly reported side effects of drugs, A 34 years of male reported –~~

~~*"When I used to take pills, I had severe pain in the stomach, vomiting and diarrhea, there was a burning sensation in the stomach, and also feelt dizziness and nervousness. So, I quit this medicine."*~~ ~~[Patient 10, Male, 34 years].~~

A 42 years old family member (patient's wife) told - *"He was not taking medicines because he was unhappy with medicine, he had a burning sensation in his stomach and nervousness"* [Family member 4~~, Female, 42 years~~] whereas ~~.~~

~~A~~ a 25 years old male treatment supporter reported - *"Patients do not take medicine properly because medicine causes heat in the stomach, and vomiting and diarrhea occur, so they quit medicine after few days, and they approach local Gunia (traditional healer) or private practitioner* ~~*Patients do not take medicine properly because medicine heats and vomiting and diarrhea occur, so they quit medicine after few days and approach local gunia (traditional healer) or private practioner*~~*)"* [Treatment supporter 6~~, Male, 25 years~~].

However, more patients perceived adverse drug effects as the important reason for discontinuing treatment compared to the treatment providers and family members.

High Pill burden and drug quality

Formatted: Font: 14 pt, Not Italic

During interview sessions, we also found that iIn addition to adverse drug effects, a high pill burden is also an important reason issue for LTFU in TB patients in this community. The daily regimen of 9-11 pills with injection made it difficult for some patients to complete treatment mainly among drug-resistant patients. A drug-resistant patient reported –

"Though I had to take 8-10 pills every day, I was taking these pills but I was also asked to take injections. As I was already weak, I used to get dizziness after injection and go into slumber" [Patient 8, Male, 45 years].

However, few patients also perceive that apart from the quantity, the poor quality of drugs is also a reason for loss to follow-ups and the drug's poor effects.

A 34 years old drug-resistant Another patient complained of the quality of the drugs and reported- *"The pills were not of good quality, I had to take 8 pills, it was troublesome to take these medicine regularly"* [Patient 10, Male, 34 years].

The high pill burden and fear of injection among TB patients is also recognized as a reason for LTFU by treatment provides. As one treatment supporter reported "*Patients give up medicine as they have to take many pills, and due to the fear of injections* [Treatment supporter 08, Male, 25 years].

Work--related and financial constraint

Work--associated problems are also identified as one of the reasons for the discontinuation of the treatment among patients. Fourteen patients reported that they had to quit their job for to starting the treatment. Some patients mentioned difficulties in carrying out their works after taking medicines. These Saharia TB patients are economically poor and they mainly work as daily wage agriculture or a manual labourer.

Formatted: Line spacing: Double

Formatted: Font: 14 pt, Not Italic

Formatted: Font: 14 pt, Not Italic

Formatted: Line spacing: Double

A 35 years old male DR- patient patient reported *"I used to go to stone quarry work. Since I got sick and I am unable to go ~~for~~ to work. Earlier, I used to earn Rs. 50-100 daily, now that too ~~is~~ has gone"* [Patients 17,~~ Male, 35 years~~]. While few others mentioned financial constraints in continuing their treatment, and because of their poverty they have to work despite their sickness. A 42 years old male XDR patient-reported-

"*As I had taken payment in advance and was working away from my home, I could not go to my house, so I quit medicine"* [Patient 20].

When they are too weak to continue their work, their family members substitute them so that their family could sustain them. As a 30 years old DS male patient reported "*I used to earn Rs 250-300 daily, but I can no longer go to work due to my illness. So, now, my wife goes to work" [Patient 03]. A family member also reported-*

"Since he is sick, I and my son go to earn outside, I want him to get well soon and start earning so that we do not have to worry. But he has stopped taking medicine due to feeling dizziness after medication. We are troubled by working outside" [Family member 4, Female, 42 years].

~~A 50 years old male mentioned, *"When I was taking medicine, I used to get dizziness and I was afraid that I wouldn't get my wages"* [Patient 16, Male, 50 years].~~

~~Some of them are working in stone quarries and take payments in advance, so they have to continue the work even when they are sick. A 42 years old laborer reported -~~

“As I have taken payment in advance *and working away from my home, I cannot go to my house, so I quit medicine”* [Patient 20, Male, 42 years].

Alcohol abuse and smoking

Habits like alcohol use and tobacco consumption are prevalent among the Saharia community. During the interviews, many patients reported alcohol consumption and smoking as a factor for not adhering to treatment. These were also written by treatment supporters (DOTS providers) and the patient’s family members. However, after getting the disease, many of them also stopped smoking and alcohol consumption.

Habits like alcohol use and tobacco consumption is very common among *Saharia* community. During the interviews, many patients reported alcohol consumption and smoking as a factor for not adhering to treatment. These were also reported by treatment supporter (DOTS providers) and patient’s family members. However, after getting disease, many of them also stopped smoking and alcohol consumption.

A 40 years old male DS patient mentioned *“Earlier I used to smoke beedi and drink liquor, but now I have given up since I got sick”* [Patient 15]. Another A 50 years old DR male reported *“Earlier, I used to drink liquor, but now I have not consumed liquor from last 2-3 years. Now, I smoke only 2-4 Beedi per day, I have been smoking since I was 10-12 years old.”* [Patient 9, Male, 50 years]. But few patients do not stop alcohol consumption and some prefer to stop taking medicine over alcohol. A 35 years old female family member (patient’s wife) reported - “*My husband is not taking medicine because he drinks liquor. I have explained to him many times about the bad effects of liquor, but he does not listen to me. He is still suffering from disease, he coughs all the day”* [Family member, 2].

Formatted: Font: 14 pt, Not Italic

Formatted: Font: (Default) Times New Roman, 12 pt

Formatted: Justified, Line spacing: Double

Formatted: Line spacing: Double

However, few patients also consume or continue to consume alcohol to suppress the symptoms of disease and side effects of the medicine. A 45 years old DS-patient reported *"Yes, I drink liquor, I drink to suppress cough and I take approximately 200 ml at a time"* [Patient 3].

Another patient mentioned - *"Earlier I used to smoke Ganja,* (Marijuana) *and I smoked for 4-5 years, but now I have given up since last 3-4 months"* [Patient 10, Male, 34 years].

Formatted: Font: Times New Roman

A 35 years old female (patient's wife) reported - *"My husband is not taking medicine because he drinks liquor. I have explained to him many times about bad effects of liquor, but he does not listen to me. He is still suffering from disease, he coughs all the day"* [Family member 2, Female, 35 years].

A treatment supporter also mentionedsaid, "*Many patients drink liquor and smoke (Beedis) during the course of treatment, due to which the medicine cause more heat/burning sensation side effects (adhik garmi karti hai) and they give up the medicine instead of stopping drinking and smoking"* [Treatment supporter 4, Male, 29 Year].

However, some of them also reported that they are using alcohol to suppress the symptoms of the TB disease, especially the cough.

A 45 years old male told - *"Yes, I drink liquor, I drink to suppress cough and I take approximately 200 ml at a time."* [Patient 3, Male, 45 years].

Stigma and family support

Formatted: Font: 14 pt, Not Italic

Social stigma, discouragement, and low-income family support are also reported as significant causes and barriers for not completing the TB treatment. The fears of discrimination by their family members directly affect the activities to promote adherence and TB treatment outcomes. During the interview sessions, few TB patients reported that they did not want health care workers to visit their homes for counseling. They did not want to visit their local or block and district level DOT center due to fear of disclosure of their disease status.

Social stigma, discouragement and poor family support are also reported as major causes and barriers for not completing the TB treatment. The fears of discrimination by their own family members directly affect the activities to promote adherence and TB treatment outcomes. During the interview sessions, few TB patients reported that they did not want health care workers to visit their home for counseling and even they did not want to visit their local or block and district level DOT center, due to fear of disclosure of their disease status.

A 38 years old DR female patient reported - *"When my TB treatment started, I was very ashamed to tell my neighbor and the villagers. I do not know why, but there was always a fear; I did not tell anyone my treatment was for what"* [Patient 2, Female, 38 years]. A 30 years old male DR patient also mentioned *"When I got sick, no one from my family was willing to accompany me to the hospital. Only my wife was willing to come, but I did not take her to the hospital because she had never visited a hospital, she does not know anything about hospitals"* [Patient 1]. Few family members also informed that they prefer to keep the patient outside of the house to avoid infection to other family members.

Formatted: Font: (Default) Times New Roman, 12 pt

Formatted: Justified, Line spacing: Double

Formatted: Line spacing: Double

A 35 years old family member (patient's wife) told - *"We live separately, though he is my husband. He lives in a hut outside the house, because it is a communicable disease"* [Family member 2~~, Female, 35 years~~].

~~A male patient also mentioned - "*When I got sick, no one from my family was willing to accompany me to the hospital. Only my wife was willing to come, but I did not take her to hospital because she had never visited a hospital, she does not know anything*" [Patient 1, Male, 30 years].~~

Myths and misbelieve ~~misbeliefs~~

Lack of awareness ~~to~~ about the seriousness of the disease and its treatment also influenced treatment adherence adversely. Many patients who did not complete their treatment had myths and misbeliefs associated with the TB disease and treatment. Some patients do not trust diagnosis and continue to believe that they do not have TB disease. So, they ~~don't~~ are reluctant to start treatment ~~or~~ and stop taking medicine after a few days/ weeks of treatment initiation. A 45 years old male DS patient reported –

"*I do not have any disease, so why should I take medicine.*" [Patient 13~~, Male, 45 years~~].

However, some also believed that taking medicine or anti-TB treatment will further deteriorate their health.

Formatted: Font: 14 pt, Not Italic

Formatted: Font: 14 pt, Not Italic

Formatted: Line spacing: Double

A 50 years old male DR patient reported, *"I would have not been able to sit again if I had taken these pills and would have died. People in my family also used to get upset and used to say why I should take such pills"* [Patient 9~~, Male, 50 years~~].

A few of them also perceived that different patients get different anti-TB medicines. One who received good medicines he/she completes treatment, while others who don't receive good medicines they had a side effect.

A~~nother~~ 45 years old male DS~~-~~patient revealed - "*My younger brother had the same disease (TB). He also took medicine~~;~~ but the medicines he was taking were different. My medicine caused stomach aches and a burning sensation in the urine. My medicine was not good, he got better medicines"* [Patient 6~~, Male, 45 Year~~]. Whereas,

s~~S~~ome patients also expressed peer pressure to stop medicines. ~~A 60~~Sixty years old male DR-patient reported –

"I was taking medicine for a long time and when I started getting blood in the cough, my neighbors and villagers asked me to stop taking too much medicines. So, I quit medicine" [Patient 14~~, Male, 60 years~~].

During interviews, it was also observed that patients, care providers and family members also correlate misfortunes with TB disease or its treatment. A 38 years old DR female patient who lost her child during the treatment reported –

"When I was taking medicines, my son died.; I was very much disheartened and left the medicines. When my son is no more, then for whom should I take medicines?" [Patient 2, Female, 38 years].

Challenges and barriers (Representative's quotations)

Treatment supporters and patient's family members were also interviewed to identify the challenges and barriers leading to LTFU. Some of them mentioned that long duration of treatment, alcoholism and smoking are important causes of LTFU. As reported by one treatment supporter –

"As far as I have experienced, the medicines have adverse effects (garmi karti hai) and the duration of treatment is prolonged. So, patients take medicine for one or two months and then they quit medicine" [Treatment supporter 1, Male, 20 years].

Another treatment supporter 29 years old male reported -

"Many patients drink liquor daily and get drunk and do not take medicines regularly. Subsequently they stop medicine [Treatment supporter 4, Male, 29 years].

Some patients stop the medicine after taking one-two months when symptoms get subsided, thinking that they are disease--free and thus do not take a full course of treatment. A treatment supporter reported –

Formatted: Font: 14 pt, Not Italic

Formatted: Line spacing: Double

"*When patients take proper medicine regularly, they start to feel better within a month, and they start thinking that they have recovered from the disease and quit the medicines*" [Treatment supporter 2, Male, 20 years]. Similarly, another treatment supporter revealed -

"*After taking medicines for 1-2 months, patient's health improves and he think that he is cured, and start avoiding medicines, and finally stop the medicine*" [Treatment supporter 3, Male, 35 years].

The important quotations of the drug-sensitive and drug-resistant study participants are given below (Table 3) -

Table 3: Theme-wise important quotations of the drug-sensitive and drug-resistant study participants

**Drug Sensitive participants**	**Drug Resistant participants**
**Adverse drug effects**	
"Whenever I took medicine, there was severe pain in the stomach, a burning sensation in the urine, the medicine used to heat a lot! So, I quit the medicine." [Patient 3, Male, 45 years].<br><br>*"I used to vomit after taking medicine, So, I quit this medicine."* [Patient 05, Male, 25 years].	*"When I used to take medicines, I used to get blood out of my mouth, I used to vomit and had trouble in breathing! So, I quit the medicine."* [Patient 1, Male, 30 years].<br><br>*"When I used to take pills, I had severe pain in the stomach, vomiting and diarrhea, there was a burning sensation in the stomach, and also feelt dizziness and nervousness. So, I quit this medicine."* [Patient 10, Male, 34 years].
**High Pill burden and drug quality**	
	"Though I had to take 8-10 pills every day, I was taking these pills but I was also asked to take injections. As I was already weak, I used to get dizziness after injection and go into slumber" [Patient 8, Male, 45 years].<br><br>*"The pills were not of good quality, I had to take 8*

Formatted: Font: Not Bold

Formatted: Font: Not Italic

Formatted Table

Formatted Table

Formatted Table

	pills, it was troublesome to take these medicines regularly" [Patient 10, Male, 34 years].
**Work-related and financial constraint**	
"I used to go to daily wages work. Since I got sick and I am unable to go to work. Earlier, I used to earn Rs. 200-250 daily, now that too is gone otherwise, I would have earned 6-7 thousand in a month" [Patients 04, Male, 35 years].<br><br>*"I used to earn Rs. 250-300 daily; I can no longer go to work due to illness. Now wife goes to work"* [Patients 03, Male, 35 years].	*"I used to go to stone quarry work. Since I got sick and I am unable to go to work. Earlier, I used to earn Rs. 50-100 daily, now that too is gone"* [Patients 17, Male, 35 years].<br><br>*"When I was taking medicine, I used to get dizziness and I was afraid that I wouldn't get my wages"* [Patient 16, Male, 50 years].<br><br>"As I have taken payment in advance *and working away from my home, I cannot go to my house, so I quit medicine"* [Patient 20, Male, 42 years].
**Alcohol abuse and smoking**	
"Yes, I drink liquor, I drink to suppress cough and I take approximately 200 ml at a time." [Patient 3, Male, 45 years].<br><br>*"Earlier I used to smoke beedi and drink liquor, but now I have given up since I got sick" [Patient 15, Male, 40 years].*	*"Earlier, I used to drink liquor, but now I have not consumed liquor for the last 2-3 years. Now, I smoke only 2-4 Beedi per day, I have been smoking since I was 10-12 years old." [Patient 9, Male, 50 years].*<br><br>*"Earlier I used to smoke Ganja (Marijuana) and I smoked for 4-5 years, but now I have given up since last 3-4 months" [Patient 10, Male, 34 years].*
**Stigma and family support**	
"When my TB treatment started, my wife started living separately from me, I have to live in a hut (Jhopdi) outside the home and my food is also prepared separately" [Patient 04, Male, 34 years].	*"When my TB treatment started, I was very ashamed to tell my neighbor and the villagers. I do not know why, but there was always a fear; I did not tell anyone my treatment was for what"* [Patient 2, Female, 38 years].<br><br>"*When I got sick, no one from my family was willing to accompany me to the hospital. Only my wife was willing to come, but I did not take her to hospital because she had never visited a hospital,*

Formatted Table

	she does not know anything" [Patient 1, Male, 30 years].
**Myths and misbelieves**	
"I do not have any disease, so why should I take medicine." [Patient 13, Male, 45 years].<br><br>*"My younger brother had the same disease (TB). He also took medicine, but the medicines he was taking were different. My medicine caused stomach aches and a burning sensation in the urine. My medicine was not good, he got better medicines"* [Patient 6, Male, 45 Year].	*"I would have not been able to sit again if I had taken these pills and would have died. People in my family also used to get upset and used to say why I should take such pills"* [Patient 9, Male, 50 years].<br><br>*"I was taking medicine for a long time and when I started getting blood in the cough, my neighbors and villagers asked me to stop taking too much medicine. So, I quit medicine"* [Patient 14, Male, 60 years].<br><br>*"When I was taking medicines, my son died; I was very much disheartened and left the medicines. When my son is no more, then for whom should I take medicines?"* [Patient 2, Female, 38 years].

Discussion

This is the first reported qualitative study that provides insight into the causes of LTFU of TB patients among the Saharia- a PVTG in Madhya Pradesh with a very high TB burden. India's National Tuberculosis Elimination Programme reported 4% LTFU for the TB patients notified in 2018 [20]. A recent study from central India reported 8.6% LTFU among TB patients registered under NTEP [21]. Our study among Saharia tribe found high proportion of unfavorable treatment outcomes including higher rate of post-treatment mortality [22] and LTFU (15.3%) among Saharia TB patients (unpublished data). The low socio-economic status, frequent migration to other areas, under-nutrition, poor access to health facilities and inadequate reach of the health system due to remotely located Saharia habitats might be contributing to higher LTFU in this community.

Formatted: Font: 18 pt

Formatted: Font: (Default) Times New Roman, 12 pt, Pattern: Clear (White)

Formatted: Justified, Line spacing: Double

Formatted: Font: Times New Roman, 12 pt

Formatted: Font: Times New Roman, 12 pt

Formatted: Font: Times New Roman, 12 pt

Formatted: Font: Times New Roman, 12 pt

Formatted: Font: (Default) Times New Roman, 12 pt, Font color: Black, Pattern: Clear (White)

This is the first reported qualitative study that provide insight on the causes of LTFU of TB patients among *Saharia*, a PVTG in Madhya Pradesh having very high TB burden. The study focused on in-depth analyses of narrative data from patients, treatment supporters and patient's family members. Our findings revealed that approaching the health facility for diagnosis and treatment initiation (step III) and enrolment/follow-up (step IV) are very crucial for treatment adherence among TB patients (Fig I).

We found that patients' judgment of the severity of their fatigue, dizziness, vomiting, and high pill burdens also led to LTFU among the Saharia.

We found that patient's judgment of the severity of their fatigue; dizziness and vomiting and high pill burdens are also leadings to LTFU among *Saharia*. The adverse drug events (ADE) and high pill burden of TB regimen promote discontinuation of treatment or patients shift to private TB treatment, especially among DR patients. The poor awareness of the drug's possible side effects and the absence of counseling before initiating medications contribute to the LTFU. The poor awareness to drug's possible side effects, absence of counseling before initiation of the drugs contribute to the LTFU. Without proper counseling, patients drift out of the system and discontinue the treatment. Several other studies also reported similar findings among TB patients [3,21, 23-26][3,15-19]. In the community setting, particularly in remote tribal areas, when ADE occurs, patients need advice and counseling but they hardly find any health staff nearby to support them. So, they are compelled to approach local practitioners and traditional healers readily available in the hamlet/locality.

So, they are compelled to approach local practitioners and traditional healers who are easily available in the hamlet/locality. Our findings show that the patients were not fully aware of the

Formatted: Line spacing: Double

Formatted: Font: 14 pt, Bold, Font color: Auto, Pattern: Clear

Formatted: Justified, Line spacing: Double

Formatted: Font: (Default) Times New Roman, 12 pt, Pattern: Clear (White)

Formatted: Font color: Black, Pattern: Clear (White)

possible side effects of drugs and were not aware of how to cope with these adverse effects, particularly in the intensive phase of treatment.

Our findings show that the patients were not fully aware of the possible side effects of drugs and so they were not aware how to cope with these adverse effects, particularly in the intensive phase of treatment. Other studies have also reported similar findings in different regions [23,24,27] 16,17,20. The possible reason for higher ADE among Saharia tribe could be high prevalence of undernutrition among them. Patients with poor nutritional status are reported to have higher risk of hepatotoxicity which is one of the important ADE due to anti-TB drugs, thus contributing to LTFU [28,29]. The BMI rates are lower in this tribe, and adequate nutrition deprivations likely to lead to severe reactions like vomiting and nausea, thus promoting discontinuation of TB treatment among patients. The BMI rates are lower in this tribe and deprivation of adequate nutrition is likely to lead to severe reactions like vomiting and nausea, thus promoting discontinuation of TB treatment among patients. The pre-treatment counseling and active surveillance of patients' adverse reactions by the health staff are crucial to in improvinge treatment compliance.

Our work shows that alcohol consumption during treatment is a significant barrier to treatment compliance in the Saharia tribe.Our work shows that alcohol consumption during the course of treatment is a major barrier for treatment compliance in Saharia tribe. The findings are consistent with previous investigations. Alcoholism has been reported as a significant factor of patient non-compliance or adverse treatment outcomes among tuberculosis patients receiving DOTS treatment [26,30-33].–[19,21-24] Because of In view of these findings, the history of alcoholism before starting treatment would help identify potential defaulters.

Formatted: Pattern: Clear (White)

Formatted: Font: (Default) Times New Roman, 12 pt, Pattern: Clear (White)

Formatted: Line spacing: Double

Formatted: Font color: Black

Formatted: Font color: Black

Formatted: Font color: Black, Not Superscript/ Subscript

Formatted: Font color: Black

Formatted: Font: (Default) Times New Roman, 12 pt, Pattern: Clear

Formatted: Font: (Default) Times New Roman, 12 pt, Font color: Black

Formatted: Font: (Default) Times New Roman, 12 pt, Pattern: Clear

Formatted: Font: (Default) Times New Roman, 12 pt, Pattern: Clear

Formatted: Font: Times New Roman, 12 pt

Formatted: Font: Times New Roman, 12 pt

Formatted: Font: Times New Roman, 12 pt

Formatted: Font color: Black

Formatted: Justified, Line spacing: Double

Formatted: Font: Times New Roman, 12 pt

Formatted: Font: Times New Roman, 12 pt

Formatted: Font: Times New Roman, 12 pt

Formatted: Font: Times New Roman, 12 pt, Font color: Black

~~starting treatment would be useful in identifying potential defaulters.~~ We also found smoking leading to treatment discontinuation in the present study. Similar findings were observed among TB patients receiving standard TB regimen in different settings [31,34]~~[22,25]~~.

The Saharia tribe is one of Madhya Pradesh's marginalized communities, and they depend on daily work for their livelihood.

~~The Saharia tribe is one the marginalised community of Madhya Pradesh and they depend on daily work for their livelihood~~. Most of the LTFU patients are either primary earners or are in economic active ages, work primarily on the daily wages. The financial constraints and personal reasons related to work, i.e., fear of losing a job and migration within the country for the job, are key factors responsible for losing follow-up and not completing the treatment.

~~The financial constraints and personal reasons related to work, i.e. fear of losing job and migration within the country for job are also key factors responsible for loss to follow up and not completing the treatment.~~ Several other studies also reported work and financial constraints as the possible reasons for treatment discontinuation [23,30,35-37]~~[16, 21, 26-28]~~.

The Saharia tribe is a particularly vulnerable tribal group of Madhya Pradesh with extensive illiteracy. Our study also showed that most of the study participants are illiterate.

~~Saharia tribe is a particularly vulnerable tribal group of Madhya Pradesh with extensive illiteracy, our study also showed that most of the study participants are illiterate.~~ Thus, incorrect knowledge or myths and misconceptions about TB disease and its medications and treatments are common among patients and family members. The low literacy could also be why their incorrect knowledge about TB disease has been reported in several studies [26,38,39]. ~~The low literacy could also be the reason for their incorrect knowledge about the TB disease as has been reported in several studies[19,29,30]~~. This highlights the importance of appropriate communication

Formatted: Line spacing: Double

Formatted: Font: Times New Roman, 12 pt

Formatted: Justified, Line spacing: Double

Formatted: Font: Times New Roman, 12 pt

Formatted: Line spacing: Double

Formatted: Font: Times New Roman, 12 pt

Formatted: Justified, Line spacing: Double

Formatted: Font: Times New Roman, 12 pt

techniques to be adopted by the health workers while working in such communities with high illiteracy. We found the cultural interpretation for the drugs as ‘hot’, ‘heat generating’ in this tribal community, which is similar to finding a study among Vietnamese refugees [40]. We found the cultural interpretation for the drugs as ‘hot’, ‘heat generating’ in this tribal community which is similar to the finding of a study among Vietnamese refugees [31]. We also found many myths and misbeliefs prevalent among the patients and family members affecting treatment compliance. Several other studies also reported similar findings among TB patients [41,42] [32,33]. In the present study, some patients denied TB diagnosis and were trying to hide the disease. Other studies also reported similar perceptions of TB patients in different settings [43,44]. [34,35] Traditional healers are usually a part of the tribal society, and the community has complete faith in them. society and the community has full faith in them. So, they are consulted first for any ailment in addition to being easily approachable and locally available. We also found this faith in ‘Gunia’ (traditional healers) among Saharia tribe. A study in South Africa also reported a preference for traditional healers among TB patients [23].[16]

The complications and the factors associated with LTFU reinforced the view that only the medication's free distribution is not enough for treatment adherence or the cure. The complications and the factors associated with LTFU reinforced the view that only free distribution of the medication is not enough for treatment adherence or the cure. The stigma associated with tuberculosis also plays an important role in treatment compliance. We found that stigma has strong influence on family and community members. Similar influence of stigma is also reported in various studies conducted in different countries [23,24,37] [16,17,28]. Social support, support from family, friends, neighborhoods, and community are extremely important

Formatted: Font: Times New Roman, 12 pt

Formatted: Font: Times New Roman, 12 pt, Font color: Black

Formatted: Font: Times New Roman, 12 pt

Formatted: Font: Times New Roman, 12 pt, Font color: Black

Formatted: Line spacing: Double

Formatted: Font: Times New Roman, 12 pt

Formatted: Justified, Line spacing: Double

for TB treatment adherence. Our study revealed that lack of social and family support to TB patients oblige them to stop or interrupt the treatment. Thus, family and community support are essential to promote early diagnosis and complete treatment. Several other studies also reported social/family support influence, including financial and emotional support, on treatment adherence [23,24,37,45].

Several other studies also reported influence of social/family support including financial and emotional support on treatment adherence. [16,17,28,36].

Overall, the study revealed that multiple factors are responsible for LTFU among the Saharia tribe and highlighted the importance of better awareness, motivational counseling of patients and family members, and community involvement in TB diagnosis and treatment. The study also established the need for financial support and nutritional supplements for TB patients. The enhanced efforts of treatment supporters, health staff and Family & community persons are required to motivate and support the patients. Further research to devise appropriate strategies to improve treatment compliance should be undertaken as part of TB control in high endemic areas/communities.

Overall, the study revealed that multiple factors are responsible for LTFU among *Saharia* tribe and highlighted the importance of better awareness, motivational counseling of patients and family members, and involvement of community in TB diagnosis and treatment. The study also established the need of financial support and nutritional supplements to the TB patients. Thus, social and culturally acceptable interventions along with patient centric health facilities are essential to improve treatment compliance in high endemic areas/community.

Formatted: Font: Times New Roman, 12 pt

Formatted: Font: Times New Roman, 12 pt

Formatted: Font: Times New Roman, 12 pt, Font color: Black

Formatted: Line spacing: Double

Formatted: Font: Times New Roman, 12 pt

Formatted: Justified, Line spacing: Double

Formatted: Font: (Default) Times New Roman, 12 pt

Formatted: Line spacing: Double

Limitations~~:~~

The study is based on an in-depth interview of 22 Saharia tribe TB patients and provides substantial information about the factors leading to LTFU among the *Saharia* PVTG of Madhya Pradesh, but one needs to be cautious while generalizing the findings.

~~Few LTFU cases migrated to other districts and states in the search of work and were untraceable where as some demined to participate in the study or to provide a written consent and were excluded from the study. In spite of these limitations, the study provides substantial information about the factors leading to LTFU among the *Saharia* PVTG of Madhya Pradesh. However, t~~The major strength of this study is that it provides information from patients, service providers and family member's perspectives and also from both drug-susceptible (DS) and drug-resistant (DR) TB patients.

Acknowledgment~~:~~

The authors are thankful to the Director ICMR-NIRTH, Jabalpur for his support and encouragement throughout the study period. ~~Authors are grateful to Dr. Samrdihi Nigam and Dr. Mercy Aparna Lingala for their support in the study.~~ Sincere thanks are due to the district coordinators of the project for carrying out the study in remote tribal villages and all the field staff for their help during the data collection. We are also thankful to the study participants who spared their time and provided valuable information during the study.

Formatted: Font: Not Italic

Formatted: Font: 18 pt, Not Italic

Formatted: Font: 18 pt

Formatted: Line spacing: Double, Don't adjust space between Latin and Asian text, Don't adjust space between Asian text and numbers

Formatted: Font: 18 pt, Not Italic

Formatted: Font: 18 pt

Formatted: Line spacing: Double

References

1. Central TB Division, Directorate General of Health Services, Ministry of Health and Family Welfare, Government of India. India TB Report 2018, Government of India, 2018.

2. World Health Organization. ~~WHO (2002)~~ An expanded DOTS framework for effective tuberculosis control. WHO/CDS/TB/2002.297. Geneva: World Health Organization. 2002. ~~23 p.~~ Available at: http://whqlibdoc.who.int/hq/2002/WHO_CDS_TB_2002.297.

~~2. pdf. Accessed:12 October 2006.~~

3. Munro SA, Lewin SA, Smith HJ, Engel ME, Fretheim A, Volmink J. Patient adherence to tuberculosis treatment: A systematic review of qualitative research. PLoS Med. 2007; 4: e238. doi: 10.1371/journal. pmed.0040238 PMID: 17676945.

4. Mitchison DA. ~~(1998)~~ How drug resistance emerges as a result of poor compliance during short course chemotherapy for tuberculosis. Int J Tuberc Lung Dis. 1998; 2: 10–15.

5. World Health Organization. ~~(2013)~~ Definitions and Reporting Framework for Tuberculosis 2013 revision. WHO/HTM/TB/2013.2 Geneva, Switzerland: WHO.2013.

6. Chang SH, Cataldo JK. A systematic review of global cultural variations in knowledge, attitudes and health responses to tuberculosis stigma. Int J Tuberc Lung Dis. 2014; 18: 168–173. doi: 10.5588/ijtld.13. 0181 PMID: 24429308.

7. Hasker E, Khodjikhanov M, Sayfiddinova S, Rasulova G, Yuldashova U, Uzakova G, et al. Why do tuberculosis patients default in Tashkent City, Uzbekistan? A qualitative study. Int J Tuberc Lung Dis. 2010; 14: 1132–1139. PMID: 20819258.

Commented [h1]: Correct numbering

Formatted: Font: 18 pt

Formatted: Font: 18 pt

Formatted: Line spacing: Double

Formatted: Font: Times New Roman

Formatted: Font: 11 pt, Font color: Custom Color(RGB(91,97,107))

Formatted: Font: Times New Roman

Formatted: Line spacing: Double

Formatted: Font: Times New Roman

Formatted: Font: (Default) Times New Roman, 12 pt

Formatted: Font color: Custom Color(RGB(91,97,107))

Formatted: Font: Times New Roman

8. Gler M, Podewils L, Munez N, Galipot M, Quelapio M, Tupasi T. ~~(2012)~~ Impact of patient and program factors on default during treatment of multidrug-resistant tuberculosis. Int J Tuberc Lung Dis. 2012; ~~JJul~~ 16(7):955–960. doi: 10.5588/ijtld.11.0502 PMID: 22584124.

9. Kurbatova EV, Taylor A, Gammino VM, Bayona J, Becerra M, Danilovitz M, et al. ~~(2012)~~ Predictors of poor outcomes among patients treated for multidrug-resistant tuberculosis at DOTS-plus projects, Tuberculosis. 2012; ~~92 (2012);~~ 92(5):397–403. doi: 10.1016/j.tube.2012.06.003 PMID: 22789497.

10. Basai S, Saha KB, Sharma RK, Muniyandi M, Singh N. An overview of Tribal Population in India. Tribal Health Bulletin. 2014; 20(special issue) https://nirth.res.in/publications/tribal_health_bulletin/thb_special_issue2014.pdf.

11. IIPS. National Family Health Survey-4 (2015-16), ~~2017.~~ Mumbai: International Institute for Population Sciences (IIPS). 2017.

12. Sati VP. Sahariya Tribe: Society, Culture, Economy and Habitation. Ann Nat Sci. 2015;1(1): 26-31.

~~11.~~13. Rajak J. Nutritional and Socio-Economic Status of Saharia Tribes In Madhya Pradesh. Int J Hum Soc Sci. 2016; 6(1):79-85.

~~12.~~14. Chakma T, Rao PV, Pall S, Kaushal LS, Datta M, Tiwary RS. Survey of pulmonary tuberculosis in a primitive tribe of Madhya Pradesh. Indian J Tuberc. 1996; 43: 85–89.

~~13.~~15. Rao VG, Gopi PG, Bhat J, Selvakumar N, Yadav R, Tiwari B, et al. ~~Gadge V, Bhondeley MK, Wares F.~~ Pulmonary tuberculosis: a public health problem amongst the

Formatted: Font: Times New Roman

Formatted: Font: Times New Roman

Formatted: Font: Times New Roman

Formatted: Line spacing: Double

Formatted: Font: Not Italic

Saharia, a primitive tribe of Madhya Pradesh, Central India. Int J Inf Dis. 2010; 14 (8):e713–716.

16. Rao VG, Bhat J, Yadav R, Muniyandi M, Sharma R, Bhondeley MK. Pulmonary tuberculosis - a health problem amongst Saharia tribe in Madhya Pradesh. Indian J Med Res. 2015~~; May~~; 141(5): 630–635.

17. Rao VG, Bhat J, Yadav R, Sharma RK and Muniyandi M. Declining tuberculosis prevalence in Saharia, a particularly vulnerable tribal community in Central India: evidences for action. BMC Infect Dis. 2019;19(1):180. doi: 10.1186/s12879-019-3815-8.

18. Prakash R, Kumar D, Gupta VK, Jain S, Chauhan DS, Tiwari PK, et al. Status of multidrug resistant tuberculosis (MDR-TB) among the Sahariya tribe of North Central India. J Infect Public Health. 2016;9(3):289-297. doi: 10.1016/j.jiph.2015.10.008. Epub 2016 Jan 6.PMID: 26775848.

19. Bhat J, Rao VG, Yadav R, Muniyandi M, Sharma R, Karfarma C, et al. Situation of drug resistant tuberculosis in Saharia tribe of central India. Indian J Med Res. 2015;141(5):636-639. doi: 10.4103/0971-5916.159562.PMID: 26139782.

~~14.~~20. Central TB Division. India TB Report, 2020. Annual Report. Ministry of Health and Family Welfare. Government of India.2020.

21. Mundra A, Deshmukh PR, Dawale A. Magnitude and determinants of adverse treatment outcomes among tuberculosis patients registered under Revised National Tuberculosis Control Program in a Tuberculosis Unit, Wardha, Central India: A record-based cohort study: J Epidemiol Glob Health. ~~Journal of Epidemiology and Global Health~~ 2017; 7: 111–118. http://dx.doi.org/10.1016/j.jegh.2017.02.002.

Formatted: Font: Not Italic

Formatted: Font: Not Italic

Formatted: Font color: Auto, Pattern: Clear

Formatted: Space After: 10 pt, Line spacing: Double

Formatted: Line spacing: Double

Formatted: Font: (Default) Times New Roman, 12 pt, Not Bold, Font color: Auto, Pattern: Clear

~~15.~~22. Rao VG, Muniyandi M, Sharma RK, Yadav R, Bhat Jyothi. Long term survival of patients treated for tuberculosis: a population-based longitudinal study in a resource poor setting. Trop Med Int Health (In press).

~~16.~~23. Edginton ME, Sekatane CS, Goldstein SJ. ~~(2002)~~ Patients' beliefs: Do they affect tuberculosis control? A study in a rural district of South Africa. Int J Tuberc Lung Dis. 2002; 6: 1075–1082.

~~17.~~24. Wares DF, Singh S, Acharya AK, Dangi R. ~~(2003)~~ Non-adherence to tuberculosis treatment in the eastern Tarai of Nepal. Int J Tuberc Lung Dis. 2003; 7: 327–335.

~~18.~~25. Bhattacharya S, Singh A. Why tuberculosis control programmes fail? Role of microlevel and macrolevel factors: an analysis from India. BMJ Case Rep. 2017 Apr 21;2017:bcr2017219606. doi: 10.1136/bcr-2017-219606.

~~19.~~26. Vijay S, Kumar P, Chauhan LS, Vollepore BH, Kizhakkethil UP, Rao SG. ~~Sophia Vijay , Prahlad Kumar, Lakbir Singh Chauhan, Balasangameshwara Hanumanthappa Vollepore, Unnikrishnan Pallikkara Kizhakkethil, Sumathi Govinda Rao~~. Risk Factors Associated With Default Among New Smear Positive TB Patients Treated Under DOTS in India. PLoS One. 2010; ~~Apr 6~~;5(4):e10043. doi: 10.1371/journal.pone.0010043.

27. Singh V, Jaiswal A, Porter JDH, Ogden JA, Sarin R, Sharma PP, et al. ~~(2002)~~ TB control, poverty, and vulnerability in Delhi, India. Trop Med Int Health. 2002; 7: 693–700.

28. Padmapriyadarsini C, Shobana M, Lakshmi M, Beena T, Swaminathan S. Undernutrition & tuberculosis in India: Situation analysis & the way forward. Indian J Med Res. 2016 Jul;144(1):11-20. doi: 10.4103/0971-5916.193278.PMID: 27834321.

~~20.~~29. Lv X, Tang S, Xia Y, Wang X, Yuan Y, Hu D, et al. Adverse reactions due to directly observed treatment strategy therapy in Chinese tuberculosis patients: a

Formatted: Font: Times New Roman
Formatted: Font: Times New Roman
Formatted: Font: Times New Roman
Formatted: Font: Times New Roman
Formatted: Font: Times New Roman, 12 pt, Not Bold
Formatted: Font: Times New Roman, 12 pt
Formatted: Font: Times New Roman, 12 pt, Not Bold
Formatted: Font: Times New Roman, 12 pt
Formatted: Font: Times New Roman
Formatted: Font: Times New Roman
Field Code Changed
Field Code Changed
Field Code Changed
Field Code Changed
Formatted: Font: Times New Roman
Formatted: Font: 11 pt, Font color: Custom Color(RGB(91,97,107))
Formatted: Line spacing: Double

prospective study. PLoS One. 2013;8(6):e65037. doi:10.1371/journal.pone.0065037. PMID: 23750225.

~~21.~~30. Suhadev M, Thomas BE, Raja Sakthivel M, Murugesan P, Chandrasekaran V, Charles N, et al. Alcohol Use Disorders (AUD) among Tuberculosis Patients: A Study from Chennai, South India. PLoS ONE. 2011; 6(5): e19485. doi:10.1371/journal.pone.0019485.

~~22.~~31. Bagchi S, Ambe G and Sathiakumar N. Determinants of poor adherence to antituberculosis treatment in Mumbai, India: Int J Prev Med. ~~International Journal of Preventive Medicine~~ 2010; 1(4): 223–232.

~~23.~~32. Santha T, Garg R, Frieden TR, Chandrasekaran V, Subramani R, Gopi PG, et al. ~~(2002).~~ Risk factors associated with default, failure and death among tuberculosis patients treated in a DOTS programme in Tiruvallur District, South India, 2000. Int J Tuberc Lung Dis. 2002; 6(9): 780–788.

~~24.~~33. Duraisamy K, Mrithyunjayan S, Ghosh S, Nair SA, Balakrishnan S, Subramoniapillai J, et al. ~~Oeltmann JE, Moonan PK, Kumar AM.~~ Does Alcohol consumption during multidrug~~-~~resistant tuberculosis treatment affect outcome? A population-based study in Kerala, India. Ann Am Thorac Soc. 2014;~~ Jun~~;11(5):712-8. doi: 10.1513/AnnalsATS.201312-447OC.

~~25.~~34. Al-Hajjaj MS, Al-Khatim IM. High rate of noncompliance with antituberculosis treatment despite a retrieval system: a call for implementation of directly observed therapy in Saudi Arabia. Int J Tuberc Lung Dis. 2000;4(4):345–349.

Formatted: Font: Times New Roman

Formatted: Font: (Default) Times New Roman, 12 pt, Font color: Auto, Pattern: Clear

Formatted: Font: (Default) Times New Roman, 12 pt, Not Bold, Font color: Auto, Pattern: Clear

Formatted: Font: Times New Roman

Formatted: Font: (Default) Times New Roman, 12 pt, Font color: Auto, Pattern: Clear

Formatted: Font: Times New Roman

Formatted: Font: Times New Roman

Formatted: Font: Times New Roman

Formatted: Font: Times New Roman

~~26.~~35. Deshmukh RD, Dhande DJ, Sachdeva KS, Sreenicas A, Kumar AMV, Satyanarayna S, et al. Patient and provider Reported Reasons for loss to follow up in MDRTB Treatment: A Qualitative Study from a Drug Resistant TB Center in INDIA. PloS ONE. 2015;10(8): e0135802. Doi:10.1371/journal.pone.0135802.

~~27.~~36. Wohlleben J, Makhmudova M, Saidova F, Azamova S, ~~0~~Mergenthaler C, Verver S. Risk factors associated with loss to follow-up from tuberculosis treatment in Tajikistan: a case-control study. BMC Infectious Diseases.~~,~~ 2017; 17:543 DOI 10.1186/s12879-017-2655-7.

~~28.~~37. Khan MA, Walley JD, Witter SN, Shah SK, Javeed S. ~~(2005)~~ Tuberculosis patient adherence to direct observation: Results of a social study in Pakistan. Health Policy Plan. 2005; 20: 354–365. doi: 10.1093/heapol/czi047. Epub 2005 Sep 23.~~23.~~

~~29.~~38. Dandona R, Dandona L, Mishra A, Dhingra S, Venkatagopalakrishna K, Chauhan LS. ~~et al. (2004)~~ Utilization of and barriers to public sector tuberculosis services in India. Natl Med J India. 2004; 17(6): 292–299.~~;~~

~~30.~~39. Garrido Mda S, Penna ML, Perez-Porcuna TM, de Souza AB, Marreiro Lda S, Albuquerque BC, et al. ~~Martínez-Espinosa FE, Bührer-Sékula S.~~ Factors associated with tuberculosis treatment default in an endemic area of the Brazilian Amazon: a case control-study. PLoS One. 2012;7(6):e39134. doi: 10.1371/journal.pone.0039134. Epub 2012 Jun 12.

~~31.~~40. Nair D, George A, Chacko K. ~~(1997)~~ Tuberculosis in Bombay: New insights from poor urban patients. Health Policy Plan. 1997; 12: 77–85.

Formatted: Font: Times New Roman

Formatted: Font: Times New Roman

Formatted: Font: (Default) Times New Roman, 12 pt, Font color: Auto, Pattern: Clear

Formatted: Font: (Default) Times New Roman, 12 pt, Font color: Auto

Formatted: Font: (Default) Times New Roman, 12 pt, Font color: Auto, Pattern: Clear

Formatted: Font: Times New Roman

Formatted: Font: Times New Roman

Formatted: Font: Times New Roman

Formatted: Font: Times New Roman

~~32.~~41. Cremers AL, de Laat MM, Kapata N, Gerrets R, Klipstein-Grobusch K, Grobusch MP. Assessing the Consequences of Stigma for Tuberculosis Patients in Urban Zambia. PLoS ONE. 2015; 10(3): e0119861. doi:10.1371/journal.pone.0119861.

~~33.~~42. Musuka G, Teveredzi V, Mutenherwa F, Chingombe I, Mapingure M.~~,~~ ~~Musuka et al.~~ Tuberculosis knowledge, misconceptions/myths in adults: findings from Lesotho, Malawi, Namibia and Zambia Demographic Health Surveys (2013-2016). BMC Res Notes. 2018;11:778 https://doi.org/10.1186/s13104-018-3884-6.

~~34.~~43. Watkins RE, Plant AJ. ~~(2004)~~ Pathways to treatment for tuberculosis in Bali: Patient perspectives. Qual Health Res. 2004; 14: 691–703.

~~35.~~44. Khan A, Walley J, Newell J, Imdad N. ~~(2000)~~ Tuberculosis in Pakistan: Sociocultural constraints and opportunities in treatment. Soc Sci Med. 2000;50: 247–254.

~~36.~~45. Priedeman SM, Curtis SL, Angeles G, Mullen S, Senik T. ~~Martha Priedeman Skiles, Siân L Curtis, Gustavo Angeles , Stephanie Mullen, Tatyana Senik.~~ Evaluating the Impact of Social Support Services on Tuberculosis Treatment Default in Ukraine. PLoS One. 2018; ~~Aug 9;~~13(8):e0199513. doi: 10.1371/journal.pone.0199513. eCollection 2018.

Formatted: Font: Times New Roman

Formatted: Font: (Default) Times New Roman, 12 pt, Font color: Auto, Pattern: Clear

Formatted: Font: (Default) Times New Roman, 12 pt

Formatted: Font: Times New Roman

Formatted: Font: Times New Roman

Field Code Changed

Formatted: Font: Times New Roman

Formatted: Font: Times New Roman

Table I : Background characteristics of respondents (N=22)

Factors	Frequency (N=22)	Factors	Frequency (N=22)
**Sex**		**Migration within country**	
Males	19	Moved Yes	08
Females	03	Moved No	14
**Age group**		**Addiction**	
18-39	09	Only Alcohol	03
40-59	09	Only Smoking	05
≥60	04	Both	06
**Education**		Never	08
No formal education	18	**Current employment**	
Primary	04	Yes	06
Middle	0	No	16
Higher	0	**Employment before starting treatment but had to quit**	
**Patient category**		Yes	15
New	03	No	07
Previously treated	19	**Prior employment type**	
**Drug resistant status**		Stone quarries	05
Yes	13	Agricultural farming	06
No	09	daily wages labour	11
**Resistant type**		**BMI**	
RR-MDR	09	≥18.5	04
H-mono/Poly	02	≤18.5	18
XDR	02		
**Side effects**			
Yes	18		
No	04		

Table II: Common themes that emerged during in-depth interview with LTFU TB patients, Family members and treatment supporters.

S. No.	Themes		Frequency (N=22)
I	Living condition (Household related)	a) Head of family	Yes 16
		b) House type	Hut/Kutcha 11
		c) Mode of cooking	*Chulla* 21
		d) Place of cooking	Indoor 13
		e) Ventilation	No 12
II.	Personal and life style related	a) Education	Illiterate 17, Primary 4
		b) Alcohol	Yes 9
		c) Smoking	Yes 11
		d) Myths and misbelief	Yes 08
III.	Socio-economic factors related	a) Social stigma and discrimination	Social Stigma 5<br>Discrimination 2
		b) Lack of family and social support	Family support 3<br>Social support 4
		c) Unemployment and financial constrains	Unemployment 14<br>Finical constrains 17
		d) Migration for wages	Yes 8
IV.	Service provider related	a) Behaviour of treatment supporter	Satisfactory 22
		b) Poor counseling	Yes 07
V.	Medications related	a) Adverse drug and treatment effects	Dizziness 8, Fatigue 8, Vertigo 10, Vomiting 9, Nausea 5, Stomach ache 6
		b) Long duration regimen	Yes 8
		c) High pill burden	Yes 8

Formatted: Font: 14 pt

Formatted: Left

Formatted: Left

Formatted: Left

Formatted: Left

Formatted: Left

Figure - I: Web-diagram of Multi factors responsible for loss to follow-up (LTFU) among *Saharia* TB patients

Formatted: Font: 14 pt

Formatted: Font: Times New Roman

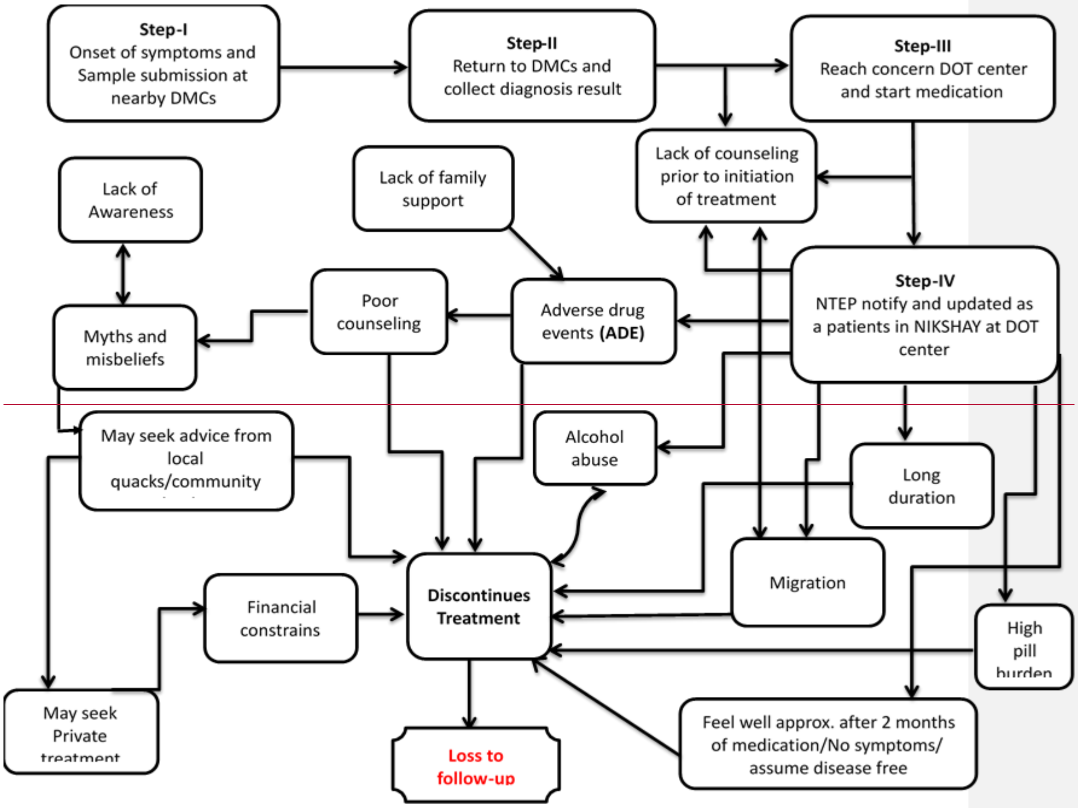

PLOS ONE

Ref.: Ms. No. PONE-D-20-21794

Factors leading to loss to follow-up (LTFU) of pulmonary TB (PTB) patients: A Study among Saharia, a particularly vulnerable tribal group of Madhya Pradesh, India

We sincerely thank reviewer(s) for their valuable observations and suggestions. We have tried our best to include all suggestions of reviewers. Point-wise reply to the reviewers' comments are given below-

**Editor comments**	**Reply to Editor/Reviewer**	**Reference**
1. Please ensure that your manuscript meets PLOS ONE's style requirements, including those for file naming. The PLOS ONE style templates can be found at<br>https://journals.plos.org/plosone/s/file?id=wjVg/PLOSOne_formatting_sample_main_body.pdf and<br>https://journals.plos.org/plosone/s/file?id=ba62/PLOSOne_formatting_sample_title_authors_affiliations.pdf	Many thanks for the suggestions. The required changes have been made as per PLOS ONE Submission Guidelines	Throughout the manuscript
2. Please include the study type in the title (i.e. qualitative study).	The study type: 'qualitative study' is included in the title.	Title
3. Please include additional information regarding the interview guide used in the study and ensure that you have provided sufficient details that others could replicate the analyses. For instance, if you developed the guide as part of this study and it is not under a copyright more restrictive than CC-BY, please	A copy of the interview guide is provided in supporting information.	

include a copy, in both the original language and English, as Supporting Information.		
4. Please discuss any land access permits or Saharia tribal permissions obtained for this research.	ICMR-NIRTH is under Ministry of Health and Family Welfare, Government of India. The main study was approved and supported by the department of health and the department of tribal welfare, Government of Madhya Pradesh.<br><br>Hence, no separate permission was required.	Mentioned under 'Ethical considerations'
5. We note that you have indicated that data from this study are available upon request. PLOS only allows data to be available upon request if there are legal or ethical restrictions on sharing data publicly. For information on unacceptable data access restrictions, please see http://journals.plos.org/plosone/s/data-availability#loc-unacceptable-data-access-restrictions.	A formal administrative and ethical clearances are required for sharing raw data/information".	
In your revised cover letter, please address the following prompts:<br><br>a) If there are ethical or legal restrictions on sharing a de-identified data set, please explain them in detail (e.g., data contain potentially identifying or sensitive patient information) and who has imposed them (e.g., an ethics committee). Please also provide contact information for a data access committee, ethics committee, or other institutional body to which data requests may be sent.		

b) If there are no restrictions, please upload the minimal anonymized data set necessary to replicate your study findings as either Supporting Information files or to a stable, public repository and provide us with the relevant URLs, DOIs, or accession numbers. Please see http://www.bmj.com/content/340/bmj.c181.long for guidelines on how to de-identify and prepare clinical data for publication. For a list of acceptable repositories, please see http://journals.plos.org/plosone/s/data-availability#loc-recommended-repositories.<br><br>We will update your Data Availability statement on your behalf to reflect the information you provide.		
**Reviewers comments**		
**Reviewer 1:**		
**Overall comments**<br>This manuscript focused on an important topic to identify the Factors leading to loss to follow up of Saharia a particularly vulnerable tribal pulmonary TB patients. Overall this research address important issue and well written paper. I recommended for publication with minor revisions and worth emphasiing the following points.	We thank the reviewer for his encouraging comments. We have tried to improve the manuscript as per the suggestions.	
**Specific comments**<br><br>**Abstract:** Need to add details on what is the design of the study, who collected data, what are the information collected.	The details as suggested have been added.	Added in Abstract under methods.
**Introduction**<br>The authors should strengthen the introduction by providing more information	As suggested, we have added details to strengthen the	Introduction-

on Rationale and novelty of the study in the introduction as they quote this is the first qualitative study undertaken among the Saharia Tribe. It was mentioned that several other studies conducted earlier in different settings have also identified many factors responsible for LTFU such as illiteracy, poverty, long duration of treatment, large number of medicines, access to health services, work related issues, smoking & alcoholism, beliefs and practices etc. However, limited studies on the factors responsible for LTFU among TB patients are available in India. Why, the authors expecting any different finding from this area, if so whether they identified any new factors responsible for loss to follow-up this population.	introduction.	last para
**Methods**		
• Drug sensitive (DS) TB patients are provided medicine through DOT providers and linked to the nearest TB DOT center, whereas, drug resistant (DR) TB patients are treated at district TB center (DTC) initially during pre-treatment evaluation period and later receive treatment from the nearest TB DOT center. But the analysis is combined with DS and DR. Why this was done separately, because the treatment itself different.	We agree with the reviewer. As suggested, the analysis of Drug sensitive (DS) and drug resistant (DR) is given separately in Table 3.	Table 3
• Status of loss to follow-up patients need explanation. Reasons for five deaths, is it due to loss to follow-up or any other reasons.	During the visit to all the recorded LTFU patients, the survey team found five dead. Though these deaths were associated with TB, the cause of death could not be ascertained as no verbal autopsy was done.	
• Authors included all the sample, why it called as a purposive sample – if so	In case of patients, all LTFU	

on what criteria?	patients were included in the study. However, purposive sampling was adopted for the selection of family members of patient and treatment supporters.	
**Results**		
• Age of the patient was 25-70 is wide range, is it OK	All the LTFU patients were included and the age range of these patients was 25-70 years.	
• Table 2 need explanation	The major findings in Table 2 are mentioned in results.	Results- Under 'Socio-demographic profile and living conditions of participants'- Below Table 1
• How drug reaction was measured, any definition used or any medical officer defined. Who assesse this problem is related to drugs	The responses on adverse drug reactions are based on the perceptions of patients and hence subjective. We have mentioned their responses during the interviews which they consider responsible for discontinuation of	

	the treatment.	
**Discussion**		
• What is the loss to follow-up in other areas, is it different from other areas, if so what is the possible reasons to be disused	It is now discussed, as suggested.	Discussion- 1st para
• Provide reference for higher ADE with under nutrition.	Appropriate reference is now provided.	Discussion- 2nd para
•How counseling will help in reducing ADE	Counseling will not directly reduce ADE. It will provide support to the patients and in turn reduce the possibility of LTFU.<br><br>We have mentioned "The poor awareness of the drug's possible side effects and the absence of counseling before initiating medications contribute to the LTFU" in discussion.	Discussion- Line 553-554
• The findings are consistent with previous investigations is it in Saharia tribe or other, if so then there is no difference between Saharia and other population.	In view of our finding that the Saharia patients were not fully aware of the possible ADE, we have mentioned the importance of proper counseling and referred studies reporting similar findings.	Discussion- Line 551-572

• The recommendations are not relevant to reduction of ADE , how better awareness, motivational counseling of patients and family members, and involvement of community in TB diagnosis and treatment will help to reduce ADE.	We have mentioned that better awareness, motivational counseling of patients and family members, and community involvement in TB diagnosis and treatment are important in reducing LTFU.<br>We agree with the reviewer that these will not reduce ADE.	Discussion-Last para; Above 'Limitations'
• "Social and culturally acceptable interventions along with patient centric health facilities are essential to improve treatment compliance in high endemic areas/community" this is not from this study findings, need to be modified.	As suggested, we have modified the text.	Discussion-Last para; Above 'Limitations'
**Reviewer 2:**		
Manuscript needs major corrections	The manuscript is revised, as suggested.	Throughout the text
The manuscript needs vast grammar corrections	We have attempted appropriate corrections in the manuscript.	Throughout the text
All the highlighted sentences are needed to be revised before to submit	All highlighted sentences are revised, as suggested.	Throughout the text
Results parts are to be revised and give briefly	As suggested, the result section is revised and updated.	Results
**Reviewer 3:**		
**General Comments:**	We thank the reviewer for very	Throughout the

An important paper that deals with a marginalized population with high incidence of TB. They are some typographical and grammatical errors that should be corrected.	useful comments. We have tried to incorporate the suggestions in the revised manuscript.	text
• The title however could be adjusted to better reflect the content of the study. Instead of factors associated with (which implies a quantitative study), perhaps: Reasons for …….	As suggested, the title is modified.	Title
• The introduction should better describe the TB treatment program, regimens and duration for MDR TB and DS TB. How often patients visit the hospitals, any community-based adherence treatment support available to patients? any social risk mitigation available to the patients?	Many thanks for this valid suggestion. We have now described the TB program, treatment regimens and treatment support in this area.	Introduction – 1st para
• More information on the TB burden among the Saharia tribe (both DS TB and MDR TB) and on magnitude of loss to follow-up among the Saharia tribe in India ((both DS TB and MDR TB). Are most patients with MDR TB in this region previously treated patients?	As suggested, the information on TB burden, LTFU and MDR TB is now included in the introduction..	Introduction – last para
**Methods:**The methods have been described however more detailed description of certain elements of the study would improve its strength e.g.<br>• The study population is mixed (patients with MDRTB and DS TB). Identification of patient quotes should indicate if which type of TB patient had. It is possible that some findings e.g. use of many pills and injectables may be specific to patients with MDR TB.	Thanks for the suggestions. We have separated the findings of DS and DR patients. Table 1 provides details about DS and DR patients. Type of TB patient is mentioned along with patient quotes in	Table 1

	results.	
• More detailed description of study design including methodological orientation of the study (theoretical framework), duration of interviews, a note on data saturation.	As suggested, the methodology section is updated	Under 'Methods'
• **Data analysis:** how many coders analyzed the data, analysis approach (were themes identified in advance or derived from the data), software used to analyze the data-if any.	Three researchers with PG qualifications and experience working with tribal communities read the transcripts independently. Codes and analyses were done manually. These transcripts were thematically analyzed to finalize the themes. The analysis was based on the literature review and analysis of collected data.<br><br>The methodology section is updated accordingly.	Under 'Methods'
• **Study findings:** a discussion of minor themes or diverse cases	The minor themes are presented in table 2.	Table 2.
• Service provider related reasons mentioned in Table 2 are not represented in the results.	Thanks for the suggestions, now, the revised result section also includes the responses of family members and treatment providers.	Under 'Results'
• Figure 1 combines the pathways of care with the reasons for loss to follow-up making it difficult to follow. The casual pathways in the figure are also	We agree that figure-1 is a little bit difficult to follow. So, we have	

difficult to understand. The diagram suggests a casual pathway between ADE and family support or ADE and poor counselling.	removed figure-1 from the manuscript.	
Discussion:<br>• "Our findings revealed that approaching the health facility for diagnosis and treatment initiation (step-III) and enrolment/follow-up (step-IV) are very crucial for treatment adherence among TB patients (Fig-I)". This statement is not supported by the results.	Figure-1 and statement is deleted.	Under 'Discussion' ,1st para, last sentence
• **Study Limitations:** Qualitative studies do not usually discuss their strengths and weaknesses in the way that quantitative studies do.	The study limitations are modified.	Under 'Discussion' , last para
Reviewer #4:		
This study among the tribal population is a worthy addition to the knowledge in this area. However, this appears to be a lost opportunity as some very important factors were not explored in detail to observe the link and the direction of relationship, leading to a weak conclusion without appropriate recommendations coming from the study. other comments are as follows.	We thank the reviewer for very useful comments. We have tried our best to improve the manuscript as per the comments.	
1. Factors for LTFU would be different for the drug-sensitive and drug resistant TB. They should be presented in a stratified manner. Combining them would lead to loss of information for both type of cases.	The analysis of Drug-sensitive and drug-resistant TB are separated.	Table 2 and 3.
2. The type of analysis, whether content, framework or grounded theory, is not mentioned.	A thematic framework was developed to examine the relationship between themes and	Under 'Methods'

	understand the various reasons for loss to follow-up.<br><br>The details are added in the methodology section.	
3. The topic is well researched and most factors are already known. As authors approach the analysis with a knowledge of these factors, they may actually end up doing a deductive coding instead of indicative coding as stated.	The analysis approach was based on the literature review and assessment of collected qualitative data, hence many codes and themes are based on previous studies.	Under 'Methods'
4. In the methods, it is mentioned as semi-structured whereas results mention the interviews as in-depth. Both are not same.	The interview guide includes some semi-structured questions related to background characteristics (gender, education, previous history of TB, family history of TB, DR/DS), the rest all the questions related to LTFU were open-ended.	Under 'Methods'
5. Whether the interviewers were trained in conduct of qualitative interviews?	Yes, all interviewers were trained in qualitative data collection.	Under 'Methods'
6. Responses must have been transcribed in the local language and then translated into English.	All responses were first translated into Hindi and later these transcriptions were translated into English by two researchers.	Under 'Methods'

7. The web diagram does not seem to be derived from the responses of participants rather from the investigators' prior knowledge of the relationships as there is no linking of the factors in the results section. The links only shows up in the diagram.	As suggested by the reviewers, we have deleted figure 1.	
8. Steps 1 and 2 in the web diagram are not relevant as there are no factors acting at those levels.	Figure 1 is deleted	
9. In the discussion section, the web diagram is not discussed. Each factor has been dealt independently as in quantitative studies.	Now, the web diagram is deleted	
10. Overall language correction is needed.	Thanks for the suggestion. We have tried to improve the language throughout the manuscript<br><br>.	Throughout the text
