# Supplementary material for: Reasons for loss to follow-up (LTFU) of pulmonary TB (PTB) patients: A qualitative study among Saharia, a particularly vulnerable tribal group of Madhya Pradesh, India
Source: PLoS One. 2021 Dec 23;16(12):e0261152. doi: 10.1371/journal.pone.0261152 (PMC8699669; doi:10.1371/journal.pone.0261152)
Supplement: Attachment — Submitted filename: Comments for the paper entitled_LTFU_Saharia.docx [file pone.0261152.s004.docx]

**Comments for the paper** entitled “Factors leading to loss to follow up (LTFU) of pulmonary TB (PTB) patients: A study among Saharia a particularly vulnerable tribal group of Madhya Pradesh, India”

**Overall** comments

This manuscript focused on an important topic to identify the Factors leading to loss to follow up of Saharia a particularly vulnerable tribal pulmonary TB patients. Overall this research address important issue and well written paper I recommended for publication with minor revisions and worth emphasising the following points.

**Specific comments**

**Abstract:** Need to add details on what is the design of the study**,** who collected data, what are the information collected.

**Introduction**

The authors should strengthen the introduction by providing more information on Rationale and novelty of the study in the introduction as they quote this is the first qualitative study undertaken among the Saharia Tribe. It was mentioned that several other studies conducted earlier in different settings have also identified many factors responsible for LTFU such as illiteracy, poverty, long duration of treatment, large number of medicines, access to health services, work related issues, smoking & alcoholism, beliefs and practices etc. However, limited studies on the factors responsible for LTFU among TB patients are available in India. Why, the authors expecting any different finding from this area, if so whether they identified any new factors responsible for loss to follow-up this population.

**Methods**

- Drug sensitive (DS) TB patients are provided medicine through DOT providers and linked to the nearest TB DOT center, whereas, drug resistant (DR) TB patients are treated at district TB center (DTC) initially during pre-treatment evaluation period and later receive treatment from the nearest TB DOT center. But the analysis is combined with DS and DR. Why this was done separately, because the treatment itself different.
- Status of loss to follow-up patients need explanation. Reasons for five deaths, is it due to loss to follow-up or any other reasons.
- Authors included all the sample, why it called as a purposive sample – if so on what criteria?

**Results**

- Age of the patient was 25-70 is wide range, is it	OK
- Table 2 need explanation
- How drug reaction was measured, any definition used or any medical officer defined. Who assesse this problem is related to drugs

**Discussion**

- What is the loss to follow-up in other areas, is it different from other areas, if so what is the possible reasons to be disused
- Provide reference for higher ADE with under nutrition.
- How counselling will help in reducing ADE
- The findings are consistent with previous investigations is it in Saharia tribe or other, if so then there is no difference between Saharia and other population.
- The recommendations are not relevant to reduction of ADE	, how better awareness, motivational counseling of patients and family members, and involvement of community in TB diagnosis and treatment will help to reduce ADE.
- “Social and culturally acceptable interventions along with patient centric health facilities are essential to improve treatment compliance in high endemic areas/community” this is not from this study findings, need to be modified.
