# Supplementary material for: Reasons for loss to follow-up (LTFU) of pulmonary TB (PTB) patients: A qualitative study among Saharia, a particularly vulnerable tribal group of Madhya Pradesh, India
Source: PLoS One. 2021 Dec 23;16(12):e0261152. doi: 10.1371/journal.pone.0261152 (PMC8699669; doi:10.1371/journal.pone.0261152)
Supplement: Attachment — Submitted filename: Reply_to_editor-revievers_comments_Final_250621.doc [file pone.0261152.s006.doc]

**PLOS ONE**

**Ref.:  Ms. No.** PONE-D-20-21794
**Factors leading to loss to follow-up (LTFU) of pulmonary TB (PTB) patients: A Study among Saharia, a particularly vulnerable tribal group of Madhya Pradesh, India**


We sincerely thank reviewer(s) for their valuable observations and suggestions. We have tried our best to include all suggestions of reviewers. Point-wise reply to the reviewers' comments are given below-

| **Editor comments** | **Reply to Editor/Reviewer** | **Reference** |
| --- | --- | --- |
| 1. Please ensure that your manuscript meets PLOS ONE's style requirements, including those for file naming. The PLOS ONE style templates can be found at  [https://journals.plos.org/plosone/s/file?id=wjVg/PLOSOne_formatting_sample_main_body.pdf](https://www.rediffmail.com/cgi-bin/red.cgi?red=https%3A%2F%2Fjournals.plos.org%2Fplosone%2Fs%2Ffile%3Fid%3DwjVg%2FPLOSOne_formatting_sample_main_body.pdf&isImage=0&BlockImage=0&rediffng=0&rogue=2b018caecb5fea31b935044a5cf048fe4f8b792f&rdf=VGUEdANyVzUCLQYzUGw=) and  [https://journals.plos.org/plosone/s/file?id=ba62/PLOSOne_formatting_sample_title_authors_affiliations.pdf](https://www.rediffmail.com/cgi-bin/red.cgi?red=https%3A%2F%2Fjournals.plos.org%2Fplosone%2Fs%2Ffile%3Fid%3Dba62%2FPLOSOne_formatting_sample_title_authors_affiliations.pdf&isImage=0&BlockImage=0&rediffng=0&rogue=11b759a709ab2012f136033c04aa0b75306d8519&rdf=BzYAcABxB2VUe1ZjCjY=) | Many thanks for the suggestions. The required changes have been made as per PLOS ONE Submission Guidelines | Throughout the manuscript |
| 2. Please include the study type in the title (i.e. qualitative study). | The study type: ‘qualitative study’ is included in the title. | Title |
| 3. Please include additional information regarding the interview guide used in the study and ensure that you have provided sufficient details that others could replicate the analyses. For instance, if you developed the guide as part of this study and it is not under a copyright more restrictive than CC-BY, please include a copy, in both the original language and English, as Supporting Information. | A copy of the interview guide is provided in supporting information. |  |
| 4. Please discuss any land access permits or Saharia tribal permissions obtained for this research. | ICMR-NIRTH is under Ministry of Health and Family Welfare, Government of India. The main study was approved and supported by the department of health and the department of tribal welfare, Government of Madhya Pradesh.  Hence, no separate permission was required. | Mentioned under ‘Ethical considerations’ |
| 5. We note that you have indicated that data from this study are available upon request. PLOS only allows data to be available upon request if there are legal or ethical restrictions on sharing data publicly. For information on unacceptable data access restrictions, please see [http://journals.plos.org/plosone/s/data-availability#loc-unacceptable-data-access-restrictions](https://www.rediffmail.com/cgi-bin/red.cgi?red=http%3A%2F%2Fjournals.plos.org%2Fplosone%2Fs%2Fdata-availability%23loc-unacceptable-data-access-restrictions&isImage=0&BlockImage=0&rediffng=0&rogue=afc98a1f10677ab2e17e06d24e27ef450e62eb7b&rdf=UmNTIwR1UDICLQM2Bjo=). | A formal administrative and ethical clearances are required for sharing raw data/information". |  |
| In your revised cover letter, please address the following prompts:    a) If there are ethical or legal restrictions on sharing a de-identified data set, please explain them in detail (e.g., data contain potentially identifying or sensitive patient information) and who has imposed them (e.g., an ethics committee). Please also provide contact information for a data access committee, ethics committee, or other institutional body to which data requests may be sent.    b) If there are no restrictions, please upload the minimal anonymized data set necessary to replicate your study findings as either Supporting Information files or to a stable, public repository and provide us with the relevant URLs, DOIs, or accession numbers. Please see [http://www.bmj.com/content/340/bmj.c181.long](https://www.rediffmail.com/cgi-bin/red.cgi?red=http%3A%2F%2Fwww.bmj.com%2Fcontent%2F340%2Fbmj.c181.long&isImage=0&BlockImage=0&rediffng=0&rogue=57f85eb071bf4c36a1b778bf546a1a8bd2c7de69&rdf=U2JUJAV0UjABLgA1BDg=) for guidelines on how to de-identify and prepare clinical data for publication. For a list of acceptable repositories, please see [http://journals.plos.org/plosone/s/data-availability#loc-recommended-repositories](https://www.rediffmail.com/cgi-bin/red.cgi?red=http%3A%2F%2Fjournals.plos.org%2Fplosone%2Fs%2Fdata-availability%23loc-recommended-repositories&isImage=0&BlockImage=0&rediffng=0&rogue=79c3f2494e8f7d5dfa15a5ab0db3a0ae75b65085&rdf=X24Hdwd2UDJRfldiBTk=).  We will update your Data Availability statement on your behalf to reflect the information you provide. |  |  |
| **Reviewers comments** |  |  |
| **Reviewer 1:** |  |  |
| **Overall comments** This manuscript focused on an important topic to identify the Factors leading to loss to follow up of Saharia a particularly vulnerable tribal pulmonary TB patients. Overall this research address important issue and well written paper. I recommended for publication with minor revisions and worth emphasiing the following points. | We thank the reviewer for his encouraging comments. We have tried to improve the manuscript as per the suggestions. |  |
| **Specific comments**  **Abstract:** Need to add details on what is the design of the study, who collected data, what are the information collected. | The details as suggested have been added. | Added in Abstract under methods. |
| **Introduction** The authors should strengthen the introduction by providing more information on Rationale and novelty of the study in the introduction as they quote this is the first qualitative study undertaken among the Saharia Tribe. It was mentioned that several other studies conducted earlier in different settings have also identified many factors responsible for LTFU such as illiteracy, poverty, long duration of treatment, large number of medicines, access to health services, work related issues, smoking & alcoholism, beliefs and practices etc. However, limited studies on the factors responsible for LTFU among TB patients are available in India. Why, the authors expecting any different finding from this area, if so whether they identified any new factors responsible for loss to follow-up this population. | As suggested, we have added details to strengthen the introduction. | Introduction- last para |
| **Methods** |  |  |
| • Drug sensitive (DS) TB patients are provided medicine through DOT providers and linked to the nearest TB DOT center, whereas, drug resistant (DR) TB patients are treated at district TB center (DTC) initially during pre-treatment evaluation period and later receive treatment from the nearest TB DOT center. But the analysis is combined with DS and DR. Why this was done separately, because the treatment itself different. | We agree with the reviewer. As suggested, the analysis of Drug sensitive (DS) and drug resistant (DR) is given separately in Table 3. | Table 3 |
| • Status of loss to follow-up patients need explanation. Reasons for five deaths, is it due to loss to follow-up or any other reasons. | During the visit to all the recorded LTFU patients, the survey team found five dead. Though these deaths were associated with TB, the cause of death could not be ascertained as no verbal autopsy was done. |  |
| • Authors included all the sample, why it called as a purposive sample – if so on what criteria? | In case of patients, all LTFU patients were included in the study. However, purposive sampling was adopted for the selection of family members of patient and treatment supporters. |  |
| **Results** |  |  |
| • Age of the patient was 25-70 is wide range, is it OK | All the LTFU patients were included and the age range of these patients was 25-70 years. |  |
| • Table 2 need explanation | The major findings in Table 2 are mentioned in results. | Results- Under ‘Socio-demographic profile and living conditions of participants’-  Below Table 1 |
| • How drug reaction was measured, any definition used or any medical officer defined. Who assesse this problem is related to drugs | The responses on adverse drug reactions are based on the perceptions of patients and hence subjective. We have mentioned their responses during the interviews which they consider responsible for discontinuation of the treatment. |  |
| **Discussion** |  |  |
| • What is the loss to follow-up in other areas, is it different from other areas, if so what is the possible reasons to be disused | It is now discussed, as suggested. | Discussion- 1^st^ para |
| • Provide reference for higher ADE with under nutrition. | Appropriate reference is now provided. | Discussion- 2^nd^ para |
| •How counseling will help in reducing ADE | Counseling will not directly reduce ADE. It will provide support to the patients and in turn reduce the possibility of LTFU.  We have mentioned “The poor awareness of the drug’s possible side effects and the absence of counseling before initiating medications contribute to the LTFU” in discussion. | Discussion- Line 553-554 |
| • The findings are consistent with previous investigations is it in Saharia tribe or other, if so then there is no difference between Saharia and other population. | In view of our finding that the Saharia patients were not fully aware of the possible ADE, we have mentioned the importance of proper counseling and referred studies reporting similar findings. | Discussion- Line 551-572 |
| • The recommendations are not relevant to reduction of ADE , how better awareness, motivational counseling of patients and family members, and involvement of community in TB diagnosis and treatment will help to reduce ADE. | We have mentioned that better awareness, motivational counseling of patients and family members, and community involvement in TB diagnosis and treatment are important in reducing LTFU.  We agree with the reviewer that these will not reduce ADE. | Discussion- Last para; Above ‘Limitations’ |
| • “Social and culturally acceptable interventions along with patient centric health facilities are essential to improve treatment compliance in high endemic areas/community” this is not from this study findings, need to be modified. | As suggested, we have modified the text. | Discussion- Last para; Above ‘Limitations’ |
| **Reviewer 2:** |  |  |
| Manuscript needs major corrections | The manuscript is revised, as suggested. | Throughout the text |
| The manuscript needs vast grammar corrections | We have attempted appropriate corrections in the manuscript. | Throughout the text |
| All the highlighted sentences are needed to be revised before to submit | All highlighted sentences are revised, as suggested. | Throughout the text |
| Results parts are to be revised and give briefly | As suggested, the result section is revised and updated. | Results |
| **Reviewer 3:** |  |  |
| **General Comments:** An important paper that deals with a marginalized population with high incidence of TB. They are some typographical and grammatical errors that should be corrected. | We thank the reviewer for very useful comments. We have tried to incorporate the suggestions in the revised manuscript. | Throughout the text |
| • The title however could be adjusted to better reflect the content of the study. Instead of factors associated with (which implies a quantitative study), perhaps: Reasons for ……. | As suggested, the title is modified. | Title |
| • The introduction should better describe the TB treatment program, regimens and duration for MDR TB and DS TB. How often patients visit the hospitals, any community-based adherence treatment support available to patients? any social risk mitigation available to the patients? | Many thanks for this valid suggestion. We have now described the TB program, treatment regimens and treatment support in this area. | Introduction – 1^st^ para |
| • More information on the TB burden among the Saharia tribe (both DS TB and MDR TB) and on magnitude of loss to follow-up among the Saharia tribe in India ((both DS TB and MDR TB). Are most patients with MDR TB in this region previously treated patients? | As suggested, the information on TB burden, LTFU and MDR TB is now included in the introduction.. | Introduction – last para |
| **Methods:**The methods have been described however more detailed description of certain elements of the study would improve its strength e.g. • The study population is mixed (patients with MDRTB and DS TB). Identification of patient quotes should indicate if which type of TB patient had. It is possible that some findings e.g. use of many pills and injectables may be specific to patients with MDR TB. | Thanks for the suggestions. We have separated the findings of DS and DR patients. Table 1 provides details about DS and DR patients. Type of TB patient is mentioned along with patient quotes in results. | Table 1 |
| • More detailed description of study design including methodological orientation of the study (theoretical framework), duration of interviews, a note on data saturation. | As suggested, the methodology section is updated | Under ‘Methods’ |
| • **Data analysis:** how many coders analyzed the data, analysis approach (were themes identified in advance or derived from the data), software used to analyze the data-if any. | Three researchers with PG qualifications and experience working with tribal communities read the transcripts independently. Codes and analyses were done manually. These transcripts were thematically analyzed to finalize the themes. The analysis was based on the literature review and analysis of collected data.  The methodology section is updated accordingly. | Under ‘Methods’ |
| • **Study findings:** a discussion of minor themes or diverse cases | The minor themes are presented in table 2. | Table 2. |
| • Service provider related reasons mentioned in Table 2 are not represented in the results. | Thanks for the suggestions, now, the revised result section also includes the responses of family members and treatment providers. | Under ‘Results’ |
| • Figure 1 combines the pathways of care with the reasons for loss to follow-up making it difficult to follow. The casual pathways in the figure are also difficult to understand. The diagram suggests a casual pathway between ADE and family support or ADE and poor counselling. | We agree that figure-1 is a little bit difficult to follow. So, we have removed figure-1 from the manuscript. |  |
| Discussion: • “Our findings revealed that approaching the health facility for diagnosis and treatment initiation (step-III) and enrolment/follow-up (step-IV) are very crucial for treatment adherence among TB patients (Fig-I)”. This statement is not supported by the results. | Figure-1 and statement is deleted. | Under ‘Discussion’ ,1^st^ para, last sentence |
| • **Study Limitations:** Qualitative studies do not usually discuss their strengths and weaknesses in the way that quantitative studies do. | The study limitations are modified. | Under ‘Discussion’ , last para |
| Reviewer #4: |  |  |
| This study among the tribal population is a worthy addition to the knowledge in this area. However, this appears to be a lost opportunity as some very important factors were not explored in detail to observe the link and the direction of relationship, leading to a weak conclusion without appropriate recommendations coming from the study. other comments are as follows. | We thank the reviewer for very useful comments. We have tried our best to improve the manuscript as per the comments. |  |
| 1. Factors for LTFU would be different for the drug-sensitive and drug resistant TB. They should be presented in a stratified manner. Combining them would lead to loss of information for both type of cases. | The analysis of Drug-sensitive and drug-resistant TB are separated. | Table 2 and 3. |
| 2. The type of analysis, whether content, framework or grounded theory, is not mentioned. | A thematic framework was developed to examine the relationship between themes and understand the various reasons for loss to follow-up.  The details are added in the methodology section. | Under ‘Methods’ |
| 3. The topic is well researched and most factors are already known. As authors approach the analysis with a knowledge of these factors, they may actually end up doing a deductive coding instead of indicative coding as stated. | The analysis approach was based on the literature review and assessment of collected qualitative data, hence many codes and themes are based on previous studies. | Under ‘Methods’ |
| 4. In the methods, it is mentioned as semi-structured whereas results mention the interviews as in-depth. Both are not same. | The interview guide includes some semi-structured questions related to background characteristics (gender, education, previous history of TB, family history of TB, DR/DS), the rest all the questions related to LTFU were open-ended. | Under ‘Methods’ |
| 5. Whether the interviewers were trained in conduct of qualitative interviews? | Yes, all interviewers were trained in qualitative data collection. | Under ‘Methods’ |
| 6. Responses must have been transcribed in the local language and then translated into English. | All responses were first translated into Hindi and later these transcriptions were translated into English by two researchers. | Under ‘Methods’ |
| 7. The web diagram does not seem to be derived from the responses of participants rather from the investigators’ prior knowledge of the relationships as there is no linking of the factors in the results section. The links only shows up in the diagram. | As suggested by the reviewers, we have deleted figure 1. |  |
| 8. Steps 1 and 2 in the web diagram are not relevant as there are no factors acting at those levels. | Figure 1 is deleted |  |
| 9. In the discussion section, the web diagram is not discussed. Each factor has been dealt independently as in quantitative studies. | Now, the web diagram is deleted |  |
| 10. Overall language correction is needed. | Thanks for the suggestion. We have tried to improve the language throughout the manuscript  . | Throughout the text |
