# Supplementary material for: Reasons for loss to follow-up (LTFU) of pulmonary TB (PTB) patients: A qualitative study among Saharia, a particularly vulnerable tribal group of Madhya Pradesh, India
Source: PLoS One. 2021 Dec 23;16(12):e0261152. doi: 10.1371/journal.pone.0261152 (PMC8699669; doi:10.1371/journal.pone.0261152)
Supplement: Attachment — Submitted filename: Reply_Reviewers_PlosOne_191021.doc [file pone.0261152.s008.doc]

**Reasons for loss to follow-up (LTFU) of pulmonary TB (PTB) patients: A qualitative study among Saharia, a particularly vulnerable tribal group of Madhya Pradesh, India**

**PONE-D-20-21794R1**

| **Comments** | **Action Taken** | **Reference in the text** |
| --- | --- | --- |
| Reviewer #2: Minor corrections required Minor grammar mistakes are to be corrected and unwanted, has to be removed where it does not required | Many thanks for the suggestions. We have now tried to improve the text. | Throughout the text. |
| Reviewer #3: I thank the authors for taking the time to respond to initial comments raised. I have a few minor additional comments to bring to the authors' attention | Thanks for the valuable suggestions. Necessary corrections have been made in the manuscript. | Throughout the text. |
| General comments: The authors have made significant effort to improve the manuscript. | We sincerely thank the reviewer for very useful comments and suggestions. These really helped us in improving the manuscript. | No action required. |
| Other comments  a) Under Study setting: Please indicated the lost to follow-up rates in this region from your project data | The lost to follow-up rate from our earlier study in this population is mentioned in the discussion. | Discussion first para, Line 6. |
| b) Under Study Design: Please state the theoretical framework underpinning this study | An analysis system based on a review of the literature and a preliminary evaluation of the qualitative data acquired in the context of the research question was developed. | Under Methods in Data Analysis. |
| Line 66: Write DOTs in full the first time it is used | Directly Observed Treatment, Short- course (DOTs) added in the text. | Introduction, Line 69. |
| Line 135: The sentence construction needs improvement | As suggested, the sentence is re-constructed. | Methods under Study design and sampling, Line nos. 151-152. |
| Line 155: add the length of time taken by each in depth interview | The length of time for each in-depth interview is mentioned. | Methods under Data Collection Tool and technique, Line No. 172-173. |
| Line 225: LTUF is misspelt | Corrected | Line 245 |
| Line 422: states "our study showed that most respondents were illiterate". Respondents literacy levels are not presented anywhere in the study results | It is mentioned in the results. | Results under Socio-demographic profile and living conditions of participants, Line 219 |
| Table 1: Summarize baseline characteristics instead of listing them for each participant | Table 1 was a summary table in the first draft. Participant wise information was added as per the suggestion of reviewer -1. | ---- |
| Table 3 is not necessary as it is a repetition of what is presented in the text. | Partially agree, but table 3 provides a comparative information of DR and DS patients. Further table-3 was created as per the suggestion of other reviewer. | ---- |
