# Supplementary material for: Reasons for loss to follow-up (LTFU) of pulmonary TB (PTB) patients: A qualitative study among Saharia, a particularly vulnerable tribal group of Madhya Pradesh, India
Source: PLoS One. 2021 Dec 23;16(12):e0261152. doi: 10.1371/journal.pone.0261152 (PMC8699669; doi:10.1371/journal.pone.0261152)
Supplement: Attachment — Submitted filename: Reply_Reviewers_PlosOne_121121.doc [file pone.0261152.s010.doc]

**Reasons for loss to follow-up (LTFU) of pulmonary TB (PTB) patients: A qualitative study among Saharia, a particularly vulnerable tribal group of Madhya Pradesh, India**

**PONE-D-20-21794R2**

| **Comments** | **Action Taken** | **Reference in the text** |
| --- | --- | --- |
| Reviewer #2: All the points are carefully addressed by author however manuscripts needs Minor corrections required | Many thanks for these suggestions. We have made the corrections | Throughout the text. |
| Reviewer #3: We thank the authors for taking the time to response to reviewers' comments and improve the manuscript. All my previous comments have been addressed. | We sincerely thank the reviewer for very useful comments and suggestions. These really helped us in improving the manuscript. | No action required. |
